# MMSpa is a deep learning-based tool that enhances the identification of spatial domains in spatial transcriptomics studies

**DOI:** 10.1371/journal.pbio.3003580

**Published:** 2026-01-05

**Authors:** Yi Liu, Yixiao Zhai, Pinglu Zhang, Quan Zou, Ximei Luo

**Affiliations:** 1 Institute of Fundamental and Frontier Sciences, University of Electronic Science and Technology of China, Chengdu, China; 2 Zhongguancun Academy, Beijing, China; 3 Macao Polytechnic University, Macau, China; University of New South Wales - Kensington Campus: University of New South Wales, AUSTRALIA

## Abstract

Spatial transcriptome (ST) technologies have transformed the study of tissue structure by retaining the spatial distribution of gene expression. One major challenge in accurately identifying spatial domains is to extract domain-related information from spatial locations and gene expression. Here, we propose MMSpa, a masked graph attention autoencoder framework specifically designed to improve spatial domain identification. MMSpa incorporates an edge-removal strategy to construct an enhanced spatial graph to fundamentally address cross-domain interference and characterize clearer domain boundaries. By focusing on masked gene expression reconstruction, MMSpa learns stable latent representations that capture core biological features, facilitating the identification of similar spatial subdomains and detecting domain differences across biological samples at the same developmental stage. Comparative analyses using ST datasets from multiple ST technologies and platforms demonstrated that MMSpa outperforms existing methods across various accuracy assessments. Notably, MMSpa excels in challenging scenarios involving highly heterogeneous and complex tissues, and can reveal finer-grained functional tissue domains obscured by other methods. This superior capability positions MMSpa as a powerful tool for uncovering new biological insights and compensating for the lack of spatial annotation in histopathology.

## Abstract 2

Accurately extracting domain-related information from spatial locations and gene expression is a challenge in spatial transcriptomics analysis. This study develops a masked graph attention autoencoder framework called MMSpa that enhances spatial domain identification in spatial transcriptomics by reducing cross-domain interference and clarifying tissue boundaries.

## Introduction

Spatial transcriptome (ST) sequencing technologies (e.g., 10× [1], Stereo-Seq [2], STARmap [3], osmFISH [4], MERFISH [5], etc.) enable access to gene expression at different spatial locations within tissues [6]. The additional spatial location information provided by ST data allows tissues to be segmented into specific spatial regions, representing higher-order tissue structures or spatial domains with similar gene expression patterns and spatial coherence [7–10]. Identifying spatial domains has become a fundamental initial step in ST data analysis [11–15] and is crucial for downstream analysis [8], such as visualizing tissue structures [16], discovering domain-specific marker genes [17], and exploring spatial features in development and disease [18,19].

Recently, the spatial methods based on graph neural networks (GNNs) have gained attention, such as SpaceFlow [20], conST [21], GraphST [22], SEDR [23], STAGATE [24], MAEST [25], SpaMask [26], and stCMGAE [27]. They begin by constructing a spatial graph that incorporates spatial information, and then designing different GNN modules to learn low-dimensional latent representations that integrate both spatial information and gene expression. These representations are subsequently utilized for domain identification and downstream analyses. However, these GNN-based methods struggle to simultaneously consider the following key points for domain identification: (1) constructing a spatial graph with well-defined neighborhoods for each spot. Most existing methods rely solely on the spatial proximity principle, which may not be well-suited for the specific requirements of the domain identification task and can limit the ability to accurately characterize domain boundaries, especially in highly heterogeneous or highly complex tissues, (2) ensuring the stability of latent representations, as a good representation is expected to stable with more core biological information (See “Discussion”), and (3) adaptively learning feature similarities among neighboring spots, capturing deeper biological features embedded in highly sparse ST datasets for identifying fine-grained sub-domains. Overall, accurately identifying spatial domains remains a significant challenge.

To this end, we proposed MMSpa, a graph attention (GAT) autoencoder framework featuring two masking strategies: masked feature reconstruction and re-mask decoding. The GAT module in MMSpa enables adaptive learning of local spatial neighbors, while the two masking strategies enhance model robustness, resulting in stable latent representations that capture more core biological information. Additionally, MMSpa adopts an edge removal strategy to construct an enhanced spatial graph, which can also be considered the noisy edges masking strategy, making the spatial graph more specific for the domain identification task and facilitating clearer delineation of domain boundaries. By employing mask strategies and integrating gene expression data with the enhanced spatial graph, MMSpa learns stable latent representations that improve spatial domain identification and downstream analyses, such as tissue structure visualization, Uniform Manifold Approximation and Projection (UMAP) visualization, spatial trajectory inference, pseudotime analysis, and discovery of domain-specific marker genes. We benchmarked MMSpa against nine advanced methods using 21 ST datasets generated by 10× Visium [1], Stereo-seq [2], STARmap [3], osmFISH [4], and MERFISH [5] platforms with different spatial resolutions. MMSpa consistently outperforms nine existing methods across various accuracy assessment metrics. When applied to human breast cancer datasets, MMSpa successfully distinguishes between tumor and healthy regions, revealing a similarity in the biological state between the biological states of the tumor edge and the surrounding healthy tissue. More importantly, we demonstrated MMSpa’s superior ability in uniquely identifying functional regions obscured in other methods at finer scales, whether applied to healthy or diseased tissues, which directly enhances MMSpa’s potential for uncovering new biological insights.

## Results

### Overview of MMSpa

**Fig 1:**
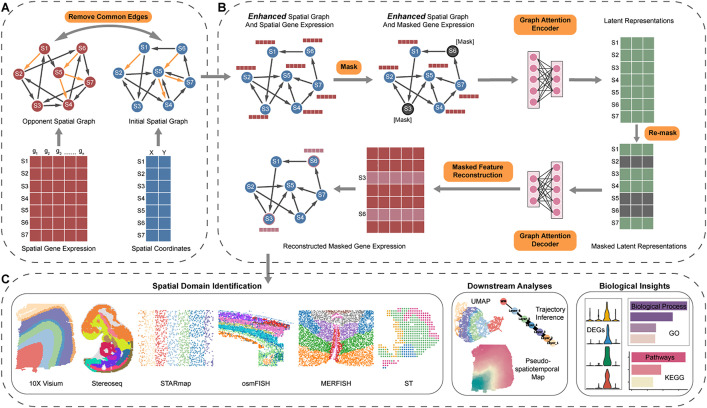
Overview of MMSpa. **(A)** MMSpa begins with the construction of the enhanced spatial graph. The initial spatial graph is constructed based on spatial coordinates. Simultaneously, the opponent spatial graph is constructed based on spatial gene expression. By removing the common edges on the initial and opponent spatial graphs from the initial spatial graph, an enhanced spatial graph can be obtained. This enhanced spatial graph serves as the input for subsequent steps. **(B)** The enhanced spatial graph and spatial gene expression are used as inputs. Initially, a masking strategy is applied to obtain masked spatial gene expression. MMSpa then feeds the enhanced spatial graph and masked spatial gene expression into a graph attention encoder. Subsequently, a re-masking strategy is employed to obtain masked latent representations. Finally, MMSpa reconstruct the gene expressions of the initially masked spots. **(C)** The obtained latent representations can be applied to spatial domain identification and other downstream analyses. MMSpa also has unique potential for uncovering new biological insights.

MMSpa first adopts an edge removal strategy to construct an enhanced spatial graph by using spatial coordinates and the spatial gene expression matrix. Specifically, MMSpa constructs an initial spatial graph based on spatial coordinates, connecting spots that are physically close to each other. Simultaneously, MMSpa constructs an opponent spatial graph, based on the distance calculated by the spatial gene expression matrix, and spots furthest from the center spot are connected to the center (Fig 1A). Then, the common edges between the initial spatial graph and the opponent spatial graph are considered noisy edges. MMSpa removes these noisy edges from the initial spatial graph to obtain the final enhanced spatial graph (Fig 1A and 1B) (see “Methods”). The edge removal strategy is specifically designed to better characterize domain boundaries, as boundary spots and their physical neighbors may not necessarily belong to the same domain. By removing noisy edges from the initial spatial graph, the enhanced spatial graph can be more specific for the domain identification task and help characterize clearer boundaries (See “Discussion”).

Subsequently, MMSpa randomly chooses a proportion of spots and masks their gene expression. The enhanced spatial graph and the masked gene expressions are then fed into a GAT encoder to generate latent representations. Then, MMSpa re-masks a proportion of spots’ latent representations. Based on these masked latent representations and a GAT decoder, MMSpa reconstructs the gene expressions of the initially masked spots (Fig 1B). Unlike existing methods, this masked feature reconstruction strategy enables MMSpa to learn stable latent representations and capture essential biological information embedded in dependencies and features among spots (See “Discussion”). Furthermore, the re-mask strategy enhances MMSpa’s learning capability (See “Discussion”). Utilizing GAT modules, MMSpa adaptively learns the varying importance between spots with their neighbors based on local spatial context. Ultimately, the obtained latent representations can be used for domain identification, UMAP visualization, trajectory inference, and Pseudo-Spatiotemporal Map (pSM) analysis (Fig 1C). The uniquely identified functional domains by MMSpa directly enhance its potential for discovering new biological insights, including finding differentially expressed genes that other methods miss, and discovering new enriched biological processes and pathways.

### Benchmarking MMSpa with nine existing methods

We conducted a benchmarking analysis to compare the performance of MMSpa with 9 existing methods (SpaceFlow [20], conST [21], GraphST [22], SEDR [23], STAGATE [24], stCMGAE [27], SpaMask [26], SpaDo [28], and MAEST [25]) by using the classical human dorsolateral prefrontal cortex (DLPFC) dataset [10] (S1 Table). The DLPFC dataset has a total of 12 slices, each slice has clear boundaries and has been previously annotated into four or six cortical layers along with white matter (WM) [10].

**Fig 2:**
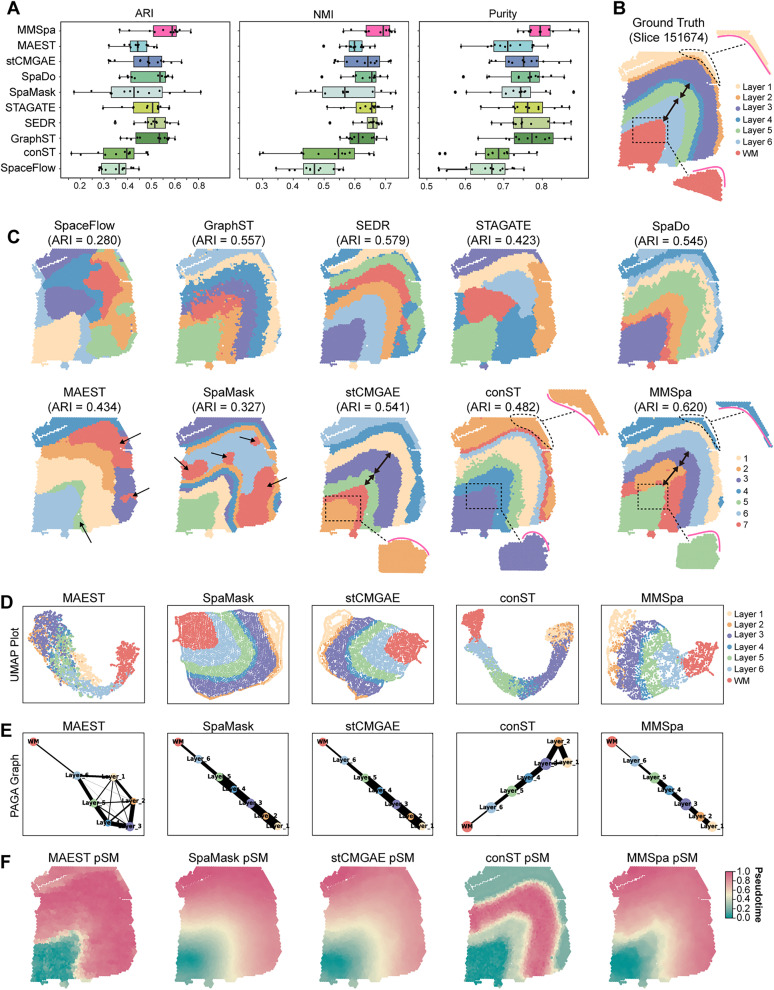
Benchmarking MMSpa with nine existing methods in the human DLPFC tissues. **(A)** Boxplots show the quantitative performance of MMSpa compared to the nine methods in domain identification accuracy across all 12 DLPFC tissue slices. The x-axis of each boxplot displays the ARI, NMI, and Purity scores, respectively. **(B)** Visualization of manual annotations for slice 151674. **(C)** Domain identification results for slice 151674 obtained by MMSpa and the other nine methods. **(D)**, **(E)**, and **(F)** Show UMAP visualization, PAGA trajectory graph, and Pseudo-Spatiotemporal Map (pSM) for slice 151674 based on the latent representations from MAEST, SpaMask, stCMGAE, conST, and MMSpa. Other methods’ visualizations are provided in S1B Fig. The underlying data for this figure can be found at https://doi.org/10.5281/zenodo.17451775.

First, we quantitatively assessed the accuracy of MMSpa and nine other methods in identifying spatial domains. Considering the annotations as ground truth, we compared domain identification results to the ground truth using three accuracy assessment metrics, including the adjusted rand index (ARI), normalized mutual information (NMI), and Purity (See “Methods”). These metrics evaluate the similarity between the domain identification results and the expected annotations, with higher scores indicating greater accuracy. Across all 12 slices, MMSpa achieved higher median scores than all nine compared methods for all three metrics (Fig 2A).

To gain more details about the domain identification results, we examined the DLPFC slice 151674 with six layers and the WM (Fig 2B and 2C). We found that SpaceFlow and STAGATE only identified WM, and failed to accurately characterize the remaining layers. SEDR and GraphST were roughly close to the expected annotation layer shapes, but each had limitations: SEDR did not correctly recover Layer 1 to Layer 3, and GraphST exhibited serrated domain boundaries. Similarly, the boundaries between layers obtained by SpaDo also exhibit a jagged pattern. We further focused on the results of three other methods that also utilize the “masking” technique in GNNs. We observed that MAEST and SpaMask had different degrees of irregular patchy contamination between layers (such as Cluster 5 and Cluster 7 in MAEST, Cluster 7 in SpaMask), resulting in unclear shapes and indistinct boundaries for each layer. In contrast, only stCMGAE and MMSpa were able to clearly delineate the cortical layers according to expected shapes, with each layer being accurately aligned in the tissue slice, especially for Layers 1–3. However, MMSpa demonstrated superior performance in predicting finer details of biological structures. Precise measurement of cortical layer thickness is crucial for diagnosing and studying various neurodegenerative and psychiatric conditions [29]. In the WM layer, which features a sharp inflection point, MMSpa accurately recovers this feature, while stCMGAE only predicts part of the WM region (Cluster 2) and the inflection point appears rounded. When combining different clusters, both stCMGAE and MMSpa can accurately predict the WM region (stCMGAE: Cluster 2 + Cluster 7, MMSpa: Cluster 5), as well as Layer 4/5 (stCMGAE: Cluster 3, MMSpa: Cluster 3), and Layer 6 (stCMGAE: Cluster 5, MMSpa: Cluster 2 + Cluster 7). In this instance, MMSpa still provides more precise biological structure predictions, especially the thickness predictions across different layers. In fact, the Layer 6 region is thicker, while Layers 4/5 are thinner. Compared to stCMGAE, MMSpa predicts the thickness of Layer 6 and Layer 4/5 more accurately, aligning better with the original biological structure. As expected, MMSpa also achieves a higher accuracy score. Interestingly, although the visual appearance of conST seems superior at first glance, it receives a lower accuracy score. The key distinction is that ARI, NMI, Purity assess the overall consistency between the predicted results and ground truth across the entire tissue section, while human vision tends to focus on large, uniform regions of color. As a result, the domain predictions made by conST, with its “large color block” effect, may give the impression of a “clear structure”. However, the global metrics that rigorously evaluate the alignment of the entire structure, which may differ from the initial visual impression. The higher score of MMSpa indicates that it performs better in terms of maintaining average consistency across all spots in the tissue slice. This is particularly noticeable in areas that may be less visually distinct, where the MMSpa can better capture finer biological details. For example, MMSpa generates much sharper and more compact boundaries between layers, while conST shows diffuse and fragmented ones. Additionally, MMSpa can more accurately recover the laminar thickness, such as Layer 1 (conST: Cluster 2, MMSpa: Cluster 4) and Layer 3 (conST: Cluster 6, MMSpa: Cluster 6). MMSpa also demonstrates superior capability in capturing the precise morphological features of cortical layers, such as the inflection point in the WM layer and Layer 2. Overall, although conST may give a clearer visual impression of layered structures at first glance, this may be a visual deception effect. Detailed biological discussions showed that MMSpa has significant advantages in global quantitative metrics (with an ARI approximately 28.6% higher than conST) (S1A Fig) and reconstruction of fine biological structures (Fig 2B). We believe MMSpa provides a more comprehensive and biologically relevant domain identification. The results for all other slices are shown in S2–S4 Figs.

Then, we compared the latent representations obtained from different methods through different downstream analyses, including the UMAP, trajectory inference (PAGA) [30], and pSM analysis (see “Methods”). Here, we used slice 151674 as an example. Applying UMAP [31] to the latent representations, we generated the 2D visualization of all spots colored by the domain annotations (Figs 2D and S1B). The UMAP plots revealed that most existing methods could only distinguish spots in WM from those in other layers. Notably, STAGATE, SpaDo, and methods with the “masking” strategy achieved clearer separation of spots in different layers (except MAEST), consistent with the hierarchical structure of the cortical layers [32]. We found that they also correctly recovered the inside-out developmental sequence from Layer 1 to Layer 6 and accurately depicted the similarities between adjacent layers (Fig 2E). These findings align with the temporal order of cortical layer development and the characteristics of neighboring layers [32]. Subsequently, we used pSM to conduct a more detailed analysis of the cortex development (Figs 2F and S1B). We found that MMSpa, STAGATE, stCMGAE, and SpaMask presented a smoother color trend, better matching the chronological order of layer development (from WM, Layer 6 to Layer 1) (Fig 2F). However, neither stCMGAE nor SpaMask can accurately depict the shape of the transition from WM to layer 6, indicating the competitiveness of MMSpa when compared to other methods that also use masking strategies. Additionally, while STAGATE showed similar pSM patterns to MMSpa, it performed poorly in domain identification. In contrast, MMSpa excelled in both domain identification and other downstream analyses. Similar findings can be observed in slices 151672 and 151508 (S5–S7A Figs).

Finally, we evaluated the robustness of MMSpa in comparison to the top five competitive deep learning methods (STAGATE, SEDR, GraphST, conST, and SpaceFlow) by varying the hyperparameter for the number of nearest neighbors (*K*), which controls the extent of local spatial smoothing and significantly influences model performance. Our results showed that, as *K* varied, the ARI scores of all six methods exhibited slight fluctuations across the 12 DLPFC slices. Notably, regardless of *K*, the median ARI score of MMSpa consistently outperformed the other five methods (S7B Fig).

Overall, these comprehensive results underscore the superiority of MMSpa, it not only significantly enhances domain identification accuracy compared to existing methods but also better captures biological phenomena through its latent representations in downstream analyses.

### MMSpa characterizes complex anatomical regions obscured in existing methods

**Fig 3:**
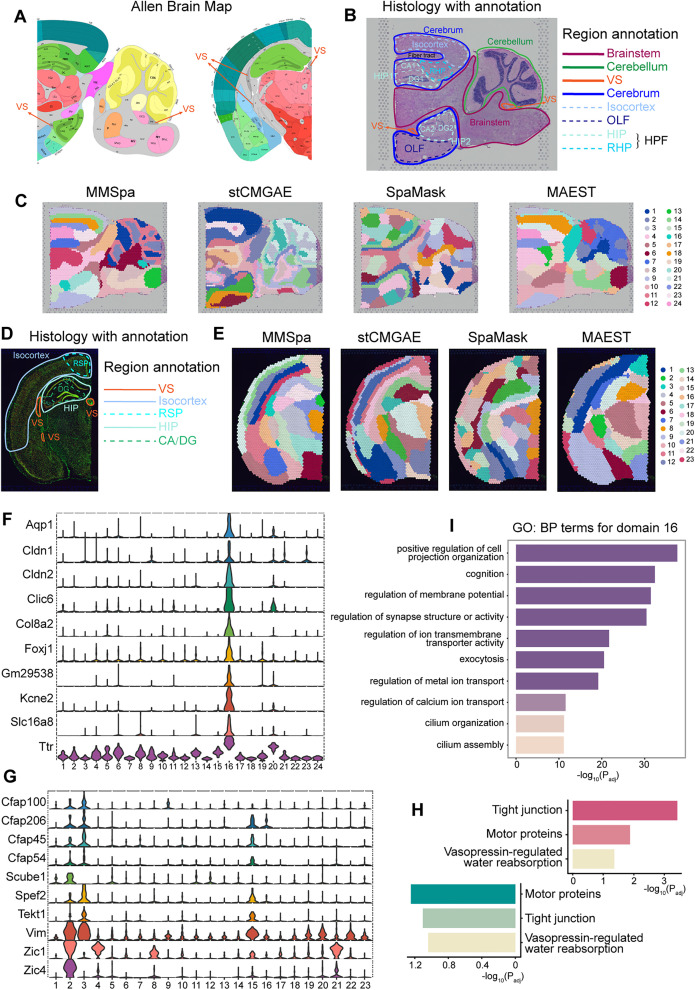
MMSpa enhances the identification of complex anatomical regions in the mouse brain tissues. **(A)** Allen brain map reference of the sagittal posterior (left) and the coronal (right) parts in the mouse brain. **(B)** Manual annotation of major regions in the sagittal posterior, as referenced from **(A)** (left), S8A Fig, and Yuan and colleagues [65]. Solid lines denote major regions, while dashed lines denote subregions. **(C)** Domain identification results for the sagittal posterior obtained by MMSpa, stCMGAE, SpaMask, and MAEST. The visualization results for other methods are shown in S8B Fig. **(D)** Manual annotation of major regions in the coronal, as referenced from **(A)** (right) and S8D Fig. **(E)** Domain identification results for the coronal using MMSpa, stCMGAE, SpaMask, and MAEST. The visualization results for other methods are shown in S8D Fig. **(F)** and **(G)** Differentially expressed genes identified in MMSpa’s domain 16 on the sagittal posterior, and domains 2 and 3 on the coronal, respectively. **(H)** The KEGG analyses for MMSpa’s domain 16 on the sagittal posterior (red, right) and domains 2 and 3 on the coronal (green, left). **(I)** The GO: BP terms for MMSpa’s domain 16 on the sagittal posterior. The underlying data for this figure can be found at https://doi.org/10.5281/zenodo.17451775.

In the previous section, we evaluated the performance of MMSpa against existing methods on the dataset featuring simple tissue structures. Here, we extend this comparison to more complex tissue structures by utilizing mouse brain datasets (S1 Table). For this comparison, we referred to the annotations in the Allen Brain map [33] (Fig 3A) and the paired histological images (Fig 3B).

First, we examined the anatomical regions of the sagittal posterior of the brain (S8A Fig). The sagittal posterior comprises three main regions: the cerebellum, brainstem, and cerebrum. The overall visualization revealed that although most methods were able to delineate the outlines of the three main brain regions, they produced scattered, cluttered, noisy, and fragmented fine-granularity domains (Figs 3C and S8B). In contrast, MMSpa demonstrated a more continuous division with sharper boundaries, which facilitates easier differentiation between adjacent domains and characterizes more complete and cleaner fine-granularity domains. Particularly, for methods which also employ masking strategies, SpaMask exhibited coarse, block-like domain results, while stCMGAE and MAEST showed notable limitations in delineating key anatomical boundaries (Fig 3C). More importantly, they lacked the ability to identify certain distinct features. For instance, the curved structure of the cerebellum was only captured by stCMGAE and MMSpa. Furthermore, among the three subregions of the cerebrum that can be divided, the isocortex exhibits a characteristic multilayered structure, which is a typical feature of the cerebral cortex. However, these methods failed to accurately capture this feature: stCMGAE did not predicted the hierarchical structure (domain 1), MAEST’s results were inaccurate (such as domain 3 and 20), and SpaMask presented patchy rather than layered structures. In contrast, MMSpa achieved accurate and clear delineations for each cortical layer (domain 3, 14, 22), offering a more detailed and biologically meaningful segmentation.

We subsequently evaluated these compared methods’ ability to characterize fine-granularity structures in the more complex HPF area. The HPF includes one retrohippocampal area (RHP) and two hippocampal areas (HIPs), with the HIPs distributed as HIP1 above and HIP2 below. The RHP connects HIP1 and the isocortex. Each HIP contains Ammon’s horn area (CA) and the dentate gyrus area (DG). Additionally, there is a small region, the fiber tract, surrounded by HIP1, RHP, and isocortex (Fig 3B). We first focused on the three masking strategies-used methods (Fig 3C). We found that SpaMask was unable to distinguish the fine-grained structures of HPF, with cross-domain mixing with different color blocks. While stCMGAE and MAEST performed better overall than SpaMask, they performed less well in fine-grained structures of complex areas like HPF. For example, stCMGAE erroneously combined CA1, CA2, and RHP (domain: 2), as well as DG1 and CA2 (domain: 11), while MAEST mixed DG2 and OLF (domain: 10). In contrast, MMSpa clearly identified the CA1, CA2, RHP, DG1, and DG2 (CA1: domain 24, CA2: domain 20, RHP: domain 23, DG1: domain 7, DG2: domain 21). This indicates that MMSpa is more competitive within masking methods, achieving superior results and revealing biological findings not captured by them. We then examined STAGATE, SEDR, and SpaceFlow (S8B Fig). Although they produced domain identification results similar to MMSpa overall, none of them were able to clearly delineate the CA and DG regions within HIP1 and HIP2. In addition, only MMSpa effectively distinguished the fiber tract (domain 22) from surrounding areas and accurately separated RHP (domain 23) from HIP1 and the isocortex, achieving high concordance with anatomical annotations (Fig 3C).

Notably, we identified a small region (domain 16) (Fig 3C) in the sagittal posterior labeled as the ventricular system (VS) by the Allen Brain Map (Fig 3A), which was corroborated by histological images showing distinct morphological features compared to surrounding regions (Figs 3B and S8A). The versus region was detectable only by MMSpa (domain 16) and GraphST (domain 5) (Figs 3C and S8B). However, GraphST’s overall visualization was noisy, leading to poorer performance in identifying other regions. Importantly, other methods failed to capture the versus region and may therefore lack the capability to identify the marker genes of the versus region. In contrast, MMSpa’s domain 16 showed differential gene expression (S8C Fig) consistent with the versus location in both the histological image and the Allen Brain Map (Fig 3A and 3B). We also verified their expression in another replicate sagittal posterior section ST dataset (S8C Fig).

Considering the complex structure of the brain, we examined an additional mouse brain dataset, focusing on a coronal section (S8D Fig). Referring to the Allen brain map (Fig 3A), several regions in the coronal are similar to the sagittal posterior, such as the isocortex, the HIP with CA1, CA2/3, and DG, and the easily overlooked versus (Fig 3D). Differently, there is a subregion named the retrosplenial area (RSP) at the top of the isocortex. We first examined the three methods that employed the masking strategies (Fig 3E). We found that SpaMask continued to struggle with capturing the multilayered features of the isocortex and did not properly differentiate between the CA and DG regions within HIP. Only stCMGAE and MMSpa were able to distinguish CA1, CA2/3, and DG. Furthermore, while stCMGAE provided a generally reasonable depiction of the coronal structure, stCMGAE failed to delineate RSP. We further analyzed the other methods and observed that SpaDo, GraphST, and conST produced scattered and noisy regions with unclear boundaries (S8D Fig). SpaceFlow failed to divide CA into subregions, and SEDR’s subregions were incorrect. Only MMSpa and STAGATE correctly delineated HIP, including DG, CA1, and CA2/3. Notably, only MMSpa successfully identified the versus region (domains 2 and 3), which was overlooked by all other methods (Fig 3E).

To ensure fairness in our comparison, we examined the domain identification results from competing methods by adjusting their clustering parameters between 20 and 25 (S9 and S10 Figs). Notably, even with different clustering parameters, the competing methods failed to detect the versus region in both brain datasets. This suggests that the limitation lies in their inability to resolve smaller spatial regions, rather than the selection of the clustering parameter. MMSpa provide an inherent advantage in detecting large-scale complex anatomical regions and their functionally distinct subdomains.

Since these two datasets lack explicit manual annotations, external consistency metrics (ARI, NMI, and Purity) cannot be used for evaluation. Therefore, we use the Silhouette Coefficient (SC) [34] and Davies–Bouldin (DB) [35] index as internal metrics to assess cluster separability and quantify the extent to which MMSpa clearly defines anatomical regions compared to other existing methods. Specifically, a higher SC and a lower DB indicate better clustering performance. Our results showed that MMSpa achieves the highest SC and the lowest DB index in both datasets, outperforming all other methods (S8E Fig). Additionally, we quantitatively evaluated the performance of each method on the two datasets by scoring each region as follows: one point was assigned for each correctly identified region, and zero points for each region that was not identified. The total score was computed for each method across both datasets. Our analysis indicated that MMSpa achieved the highest total score, outperforming all other methods (S8F and S8G Fig).

MMSpa demonstrated a clear and comprehensive delineation of all regions in both brain datasets, which remains a challenge for other methods. This highlights MMSpa’s unique ability to resolve complex anatomical structures, accurately detect subtle transitional zones between regions, and identify obscured areas. Such performance advantages directly contribute to its enhanced potential for biological discoveries. As a crucial structure in the brain, the versus is primarily responsible for the production of cerebrospinal fluid (CSF), which is then distributed throughout the cranial cavity [36,37]. The versus domains identified by MMSpa revealed DEGs that are biologically relevant to versus function and showed high concordance with known marker genes of the mouse brain versus Specifically, we found that the DEGs identified in domain 16 of the sagittal posterior mouse brain dataset exhibited strong biological relevance to CSF (Fig 3F), including genes like Ttr, abundant in CSF [38–40], as well as Cldn1, Cldn2, and Clic6, which are involved in the regulation of CSF synthesis and secretion [41]. Additional genes identified, such as Slc16a8 (involved in nutrient transport for CSF [42]) and Foxj1 (crucial for differentiation of ventricular epithelial cells [43,44]), and Aqp1 and Kcne2 (essential for maintaining ventricular homeostasis [45,46]), further emphasize the relevance of domain 16 to CSF biology. Similar results were observed in the coronal mouse brain dataset (Fig 3G), where the DEGs identified in domains 2 and 3 included known markers for the third ventricle such as Vim, Zic1, and Zic4 [47,48], as well as the Cfap family genes, which are widely expressed in the lateral ventricles [49]. Furthermore, we also identified genes that show significant and specific expression in domains 2 and 3 and are critical for ciliary function in the VS, including Spef2 [50], Tekt1 [51], and Scube1 [52].

Based on the identified DEGs, we performed KEGG [53] and Gene Ontology: Biological Process (GO: BP) enrichment [54] analyses for domain 16 of the sagittal posterior dataset and domains 2 and 3 of the coronal dataset. The KEGG analysis results from both datasets revealed high concordance with pathways related to CSF flow and the blood–CSF barrier (Fig 3H), including the Tight Junction pathway that is the fundamental for maintaining the blood–brain and blood–CSF barriers [55,56], the Motor Protein pathway that is crucial for CSF circulation [57], and the Vasopressin-Regulated Water Reabsorption pathway that is associated with Aqp1 expression in the choroid plexus and involved in regulating CSF secretion [45]. The GO: BP enrichment results from both datasets further supported these findings, exhibiting high biologically relevant to the physiology of the VS and CSF dynamics (Fig 3I). For example, in domain 16, the enriched biological processes related to exocytosis and positive regulation of cell projection organization are directly associated with CSF secretion, and the regulation of membrane potential has been shown to play a role in controlling CSF formation to regulate intracranial pressure [58]. Other enriched biological processes related to ion transport are crucial for the choroid plexus’s role in CSF secretion [59,60], and ciliogenesis is essential for regulating the flow and distribution of CSF in the ventricles [61]. Notably, we observed unexpected enrichment in synapse-related processes, aligning with recent findings that CSF-transported synaptic proteins may serve as biomarkers for cognitive disorders [62–64], suggesting that MMSpa can detect subtle neuropathological signals associated with disease. Similar GO enrichment results were also observed in domains 2 and 3 of the coronal dataset (S8H Fig).

Finally, we evaluated MMSpa and the nine other methods using an anterior mouse brain dataset (S11A Fig), which was manually annotated by Yahui Long and colleagues [22] with 52 specific domain labels (S11B Fig). Using these annotated labels as ground truth, we visualized and quantitatively assessed MMSpa with nine other methods for their ability to accurately identify complex anatomical regions (S11C Fig). MMSpa achieved the highest accuracy in identifying complex anatomical regions (an ARI score of 0.452, an NMI score of 0.745, and a Purity score of 0.653), outperforming all other methods (S11D Fig).

Together, MMSpa demonstrated superior identification capabilities on datasets with complex tissue structures compared to existing methods. Specifically, MMSpa not only excelled in characterizing small anatomical regions that were obscured in other methods but also effectively delineated complex fine-granularity subregions. Since the other methods failed to identify the complete regions, naturally, the DEGs and pathways of the missed regions might not be identifiable either. Furthermore, in evaluations involving a larger number of domain labels, MMSpa outperformed all other methods across all metrics.

### MMSpa dissects cancer heterogeneity and reveals the potential cancer region

**Fig 4:**
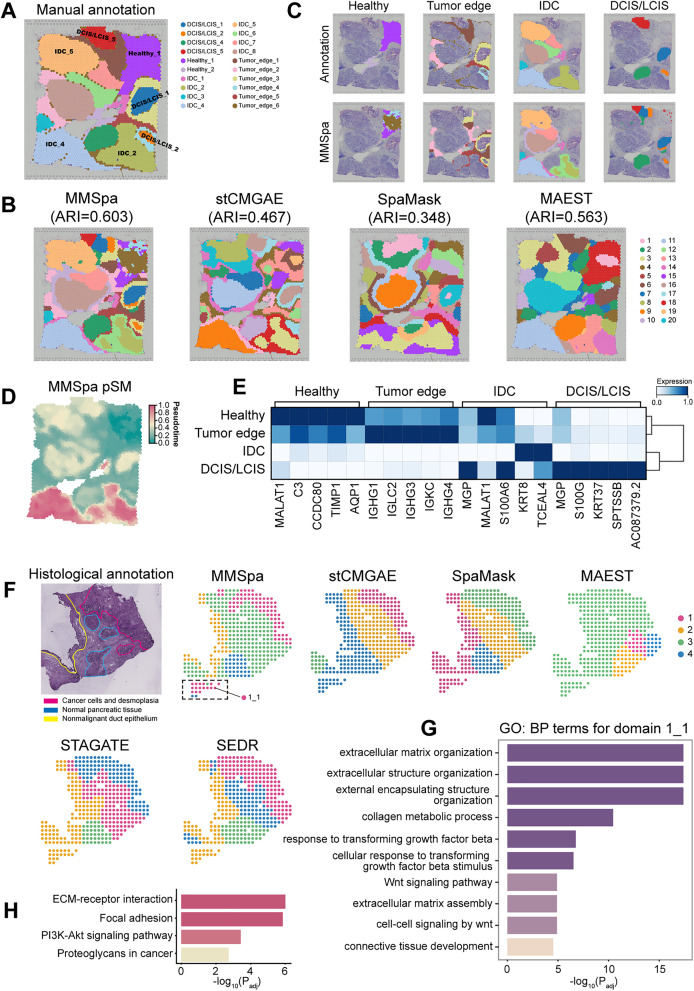
Application of MMSpa in the human breast cancer (A–E) and the PDAC dataset (F–H). **(A)** Visualization of manual annotations. **(B)** Domain identification results using MMSpa, stCMGAE, SpaMask, and MAEST. The visualization results for other methods are shown in S12C Fig. **(C)** MMSpa identifies the four distinct morphotypes annotated in **(A)**. The legends for the annotations and MMSpa results correspond to those in **(A)** and **(B)**, respectively. **(D)** Pseudo-Spatiotemporal Map (pSM) based on the latent representations from MMSpa. **(E)** Heatmaps of top differential gene expressions associated with each annotation morphotype, with hierarchical clustering of the four morphotypes based on these genes. **(F)** The PDAC hematoxylin and eosin staining with annotation from the original study [71], and spatial domains detected by existing methods on the PDAC dataset. The visualization results for other methods are shown in S12G Fig. (G) and (H) are the GO: BP terms and KEGG analysis for domain 1_1, positioned at the lower-left part of the PDAC slice. The underlying data for this figure can be found at https://doi.org/10.5281/zenodo.17451775.

Characterizing tissue structure becomes increasingly challenging when domain identification methods are applied to cancer tissues due to their inherent heterogeneity. Unlike normal tissues, cancer tissues often lack well-defined morphological regions, and areas with similar morphology may be more dispersed. Here, we applied MMSpa and nine compared methods to a dataset from human breast cancer (S1 Table and S12A Fig), a typically highly heterogeneous cancer type [66], to evaluate the methods’ efficacy in distinguishing heterogeneous cancer regions. This human breast cancer dataset was manually annotated by Hang Xu and colleagues [23] and categorized into 20 distinct regions and 4 morphotypes: healthy tissue(Healthy), ductal carcinoma in situ or lobular carcinoma in situ (DCIS/LCIS), invasive ductal carcinoma (IDC), and tumor surrounding regions with low malignancy features (Tumor edge) (Fig 4A).

We quantitatively assessed and visualized the alignment of domain identification results from each method with manual annotations (S12B Fig). MMSpa achieved the highest mean score across all three metrics, indicating superior performance in comparison to other evaluated methods. Notably, MMSpa attained the highest ARI score of 0.603, surpassing MAEST, which achieved the second-highest ARI score of 0.563. The remaining methods recorded ARI scores below 0.510. Although MAEST achieved the second-highest ARI score, its visualizations lacked clear structural detail, particularly in delineating tumor edges. For example, it failed to capture the typical tumor edge features surrounding the tumors in DCIS/LCIS_1 and DCIS/LCIS_2 (Fig 4A). These critical tumor edge features were missed entirely by MAEST (Fig 4B). We further examined other masking-based methods. While both SpaMask and stCMGAE identified four major morphotypes, their overall results were over-smoothing: exhibiting notable limitations in distinguishing regions within these morphotypes (Fig 4B). Specifically, SpaMask failed to distinguish between histologically distinct but spatially adjacent large significant tumor regions, such as IDC_5 and DCIS/LCIS_5 in the upper left of the slice, as well as IDC_4 and IDC_2 in the lower part of the slice (Fig 4A). Additionally, SpaMask exhibited poor boundary resolution, notably missing the tumor edges around DCIS/LCIS_2 (Fig 4A). Similarly, stCMGAE showed significant confusion between tumor edges and the healthy region located in the upper right of the slice. Additionally, conST and GraphST exhibit decentralized and noisy delineations (S12C Fig). SpaceFlow, SEDR, and STAGATE struggled with detail delineation, particularly in distinguishing the Tumor edge from surrounding areas. In contrast, MMSpa provided precise spatial delineation of all morphotypes and their subregions, accurately capturing fine details that other methods missed. This demonstrates its superior performance in capturing morphological details in highly heterogeneous tissues (Fig 4C). MMSpa’s enhanced performance allowed it to integrate different domains into distinct morphotypes more effectively, aligning more closely with manual annotations.

Cancer tissues exhibit not only high spatial heterogeneity but also spatial continuity. We subsequently conducted downstream analyses of different morphotypes. Initially, we examined the cell types within cancer regions (DCIS/LCIS and IDC) identified by MMSpa (domain 2, 7–14, 16, 18–20) (Fig 4C). Using the CARD [67] deconvolution algorithm with a single-cell RNA sequencing dataset [22,68], we observed a significant concentration of luminal cells in these cancer regions (S12D Fig). Luminal cell type is prevalent in breast cancer [69], and luminal tumors are known to be a common form of human breast cancer [70]. Subsequently, we computed the pSM for each spot based on latent representations, by setting the healthy region as the root. The pSM values reflected the duration of the lesions, with larger values indicating longer durations. MMSpa demonstrated superior capability in distinguishing heterogeneous regions compared to other methods (Figs 4D and S12E). Specifically, MMSpa’s pSM clearly delineated the cancer regions, a finding that was corroborated by histological images (Figs 4D and S12A). The pSM also effectively distinguished between paracancerous regions (Tumor edge and Healthy) and cancer regions (DCIS/LCIS and IDC), as well as identifying Tumor edge regions. Due to the high heterogeneity within breast cancer, the Tumor edge represents an intermediate cellular state that is neither fully healthy nor entirely tumorigenic. Our observations revealed a significant color distinction between cancer regions and Tumor edge regions in MMSpa’s pSM (Fig 4D). Notably, the pSM values for Tumor edge regions were closer to the Healthy region than the cancer regions. We conjectured that the cellular state in Tumor edge regions might share greater similarity with the Healthy regions instead of the cancer regions.

We then analyzed differential expression genes (DEGs) to further explore the cellular state relationship between Tumor edge regions and Healthy regions. First, we examined DEGs in Healthy regions identified by MMSpa (domains 4, 15, and 17) and compared them with the DEGs in annotated Healthy regions. We found that MALAT1 was among the top-ranked genes in both analyses. Importantly, MALAT1 was highly expressed not only in Healthy regions but also in Tumor edge regions (S12F Fig), reinforcing our conjecture. Based on the annotation regions, further analysis revealed that the expression patterns of the top 5 DEGs of Healthy regions were highly similar to those in Tumor edge regions, with these genes showing high expression levels in both morphotype regions (Fig 4E). Likewise, although the top 5 DEGs of Tumor edge regions also showed expression patterns closer to Healthy regions, they differed significantly from those in cancer regions (Fig 4E). A similar pattern was observed when analyzing the DEGs of cancer regions, with consistent expression trends between the two cancer regions, but exhibiting contrasting trends in the paracancerous regions (Fig 4E).

By combining the results of pSM and DEG analyses, we observed significant differences between cancer regions and paracancerous regions. Notably, the cellular state of the Tumor edge regions was found to be more similar to Healthy regions within this breast cancer dataset. These findings underscore the effectiveness of MMSpa in analyzing tissue domains with heterogeneous cell types, suggesting that the latent representations integrated by MMSpa capture more detailed biological information. Importantly, the results also demonstrate that MMSpa is not limited to datasets with high expression similarity, and it performs equally well in tissue domains with heterogeneous cell types, highlighting its broad utility. Furthermore, the downstream analysis results from MMSpa provide a novel perspective for advancing breast cancer research.

To further demonstrate the biological value of MMSpa in diseased tissue, we expanded its application to a human pancreatic ductal adenocarcinoma (PDAC) dataset [71]. The original study [71] annotated the ST section by distinguishing four main regions based on histological features: cancer cells and desmoplasia, nonmalignant duct epithelium, stroma, and normal acini-rich pancreatic tissue (Fig 4F). We first examined masking-based methods. We found that only MMSpa and SpaMask were able to accurately delineate all four main regions of the PDAC slice. In contrast, MAEST erroneously combined cancer regions with normal tissue, while stCMGAE only identified the cancer region, failing to distinguish between non-cancerous regions and the stroma. Notably, MMSpa exhibited exceptional accuracy in identifying the nonmalignant duct epithelium region. MMSpa perfectly reconstructed both the location and thickness of this region, precisely matching the original slice annotations. In comparison, stCMGAE and MAEST failed to identify this region, and although SpaMask detected it, the delineation was imprecise. These results underscore MMSpa’s competitive advantage in handling heterogeneous cancer tissues, particularly compared with masking-based methods. We further evaluated non-masking-based methods. The results showed that only conST could identify the nonmalignant duct epithelium region located at the left edge of the slice (Figs 4F and S12G). However, while conST correctly identified the position of the nonmalignant duct epithelium region, compared to MMSpa, its segmentation was thicker than the pathological appearance in the slice, leading to discrepancies in region delineation. Notably, although other methods successfully identified the primary cancer region (upper-right corner of the slice) (Figs 4F and S12G), MMSpa uniquely detected an additional region (lower-left of the slice, domain 1_1), associating it with the same spatial domain (domain 1) as the primary cancer area (Fig 4F). This region was not emphasized in the original study’s histology-based annotations, as the original study divided regions based on hematoxylin and eosin staining and brightfield imaging, with the cancerous phenotype primarily observed in the upper-right region. We supposed that the lower-left region identified by MMSpa represents a potential cancerous area. To explore this assumption further, we performed GO: BP enrichment and KEGG pathway analyses for the additional suspected cancer area (lower-left region).

The GO: BP analysis revealed molecular hallmarks of early malignancy in this region (Fig 4G). Particularly, enriched biological processes included those related to the extracellular matrix (ECM), a major tumor component involved in tumor cell proliferation, migration, invasion, and angiogenesis [72–75]. The GO results also highlighted WNT and TGF signaling pathways, both implicated in PDAC initiation and progression [76], as well as collagen metabolic processes, which are commonly overexpressed during PDAC progression [77]. Furthermore, we observed significant enrichment in connective tissue development, a hallmark of PDAC’s fibrotic tumor microenvironment [78] (Fig 4G). These findings strongly suggest that the lower-left region may be enriched with cancer cells. The KEGG analysis further validated this assumption (Fig 4H), identifying pathways associated with the tumor microenvironment, such as ECM-related pathways and the focal adhesion pathway [79], both activated in the suspected cancer area. Additionally, the PI3K-Akt signaling pathway, a hallmark of PDAC [80,81], was significantly activated, as well as the activated proteoglycan-related pathway, which plays a critical role in cancer invasion and metastasis [82,83].

These results provide compelling evidence that the lower-left region likely represents an overlooked cancerous area, which was not clearly identified in previous studies due to its less prominent histopathological features. This conclusion highlights that MMSpa, compared to other methods, has greater potential to identify previously unrecognized cancer regions and showcase the unique biological discoveries.

### MMSpa works well on Stereo-seq data to identify finer-grained mouse embryo tissue structures

The datasets used in the previous sections were generated from the 10× Visium [1] platform. To illustrate the broader applicability of MMSpa, we extended its application to two E9.5 mouse embryo (Slice #E9.5_E1_S1 and Slice #E9.5_E2_S3) datasets generated by Stereo-seq technology (S1 Table). Stereo-seq technology enables spatial transcriptomics with a large field of view and cellular resolution [2].

**Fig 5:**
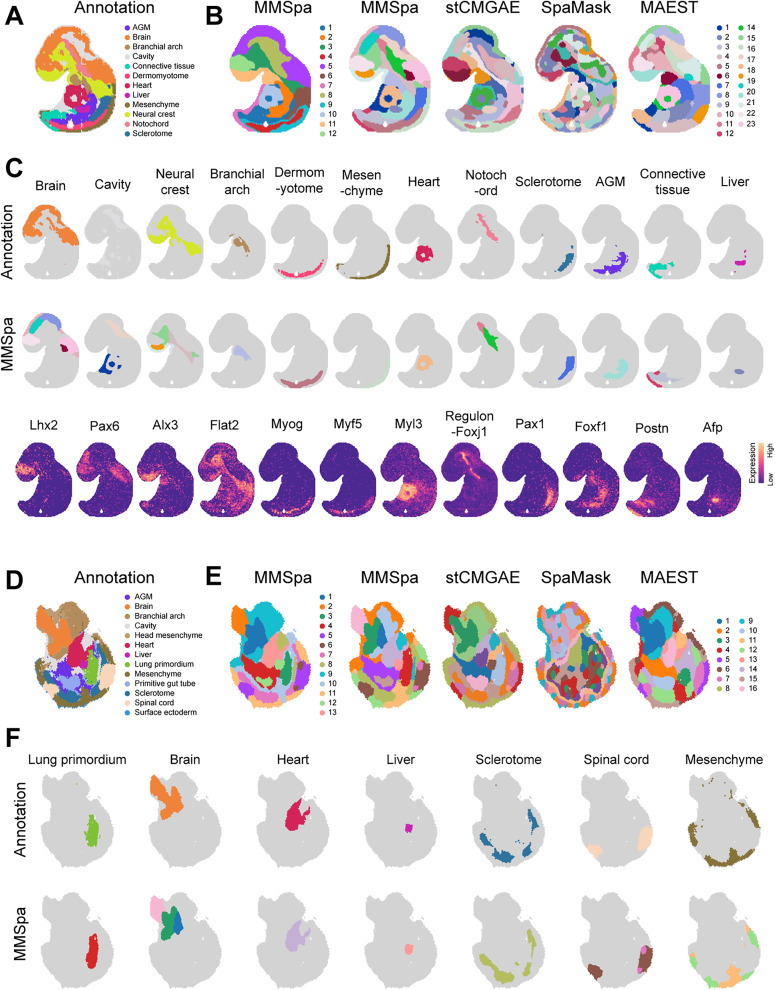
MMSpa accurately delineates finer-grained tissue structures in Stereo-seq mouse embryo datasets. **(A)** Annotations for the first applied E9.5 embryo dataset (Slice #E9.5_E1_S1). **(B)** Domain identification results of the Slice #E9.5_E1_S1 using MMSpa with 12 domains (left), and using MMSpa, stCMGAE, SpaMask, and MAEST with 23 domains (right). **(C)** Each annotated spatial region identified by MMSpa (with 23 domains) and their corresponding marker genes. **(D)** Annotations for the second applied E9.5 embryo dataset (Slice #E9.5_E2_S3). **(E)** Domain identification results of the Slice #E9.5_E2_S3 using MMSpa with 13 domains (left), and using MMSpa, stCMGAE, SpaMask, and MAEST with 16 domains (right). **(F)** The annotated spatial regions identified by MMSpa (with 16 domains). The underlying data for this figure can be found at https://doi.org/10.5281/zenodo.17451775.

The first employed E9.5 embryo dataset (Slice #E9.5_E1_S1) comprised 5,913 spots, 25,568 genes, and 12 annotated regions (Fig 5A). We initially set the cluster number to 12 to visualize and quantitatively assess the accuracy of different methods relative to the annotations (S13A and S13B Fig). MMSpa attained the highest mean score across three metrics. In particular, MMSpa achieved an ARI score of 0.405, surpassing all other methods, which scored below 0.330 (S13B Fig). Additionally, MMSpa effectively identified key anatomical regions of the embryo (Figs 5B and S13C). For example, MMSpa successfully identified the Brain (domains 12 and 5) and delineated the Telencephalon region (domain 12), a subregion of the Brain, as depicted in the original article [2]. DEG analysis for domain 12 highlighted Lhx2 as the most prominent gene, a known regulator of the Telencephalon [84]. The spatial expression of Lhx2 corresponded precisely with the spatial location of domain 12 (Fig 5C). MMSpa also segmented the Neural crest region into domain 11 and domain 3 (Fig 5A and 5B). Alx3 was the DEG marker for domain 11, while Flt2 marked domain 3. Alx3 has been reported to have a specific expression in the cranial neural crest [85], particularly in mouse embryos after E8.0 [86], whereas Flat2 has been reported to be predominantly expressed in neural crest-derived mesenchyme [87] (Fig 5C). The DEG results suggested that domain 11 represents the cranial neural crest and domain 3 corresponds to neural crest-derived mesenchyme (S13C Fig). Furthermore, MMSpa identified the Dermomyotome region (domain 4) (Fig 5A and 5B), which all other methods failed to detect (S13A Fig). The Dermomyotome region is associated with muscle tissue development [2], while domain 4 was marked by Myog, a known Dermomyotome marker [2] (Fig 5B and 5C). Similar results were observed in the identification of the Heart region (S13C Fig).

We further refined our analysis by increasing the number of clusters to 23 to capture more fine-grained structures within mouse embryos (Figs 5B and S13D). Despite this enhancement, existing methods still faced challenges in detailed structure identification. For instance, the Sclerotome, Dermomyotome, and Mesenchyme regions exhibit unique arc-shaped and linear features, and these three regions are adjacent to each other, arranged in a spatially hierarchical manner from top to bottom. We first focused on masking-based methods: stCMGAE failed to accurately capture the arc-shaped, linear features of the Dermomyotome and Mesenchyme regions. SpaMask’s results were fragmented, and even after merging the fragmented clustering results, it did not reveal the expected spatial structures or their proper arrangement. MAEST identified these three regions as large, patch-like areas. In contrast, MMSpa not only accurately captured the shape features of these three regions but also restored their spatial arrangement (Sclerotome: domain 7, Dermomyotome: domain 5, and Mesenchyme: domain 16) (Fig 5B and 5C). We also examined non-masking-based methods, and found that only SpaDo achieved the expected results. STAGATE, SpaceFlow, and SEDR failed to recognize the Dermomyotome region, while conST and GraphST overlooked the Sclerotome region (S13D Fig). Additionally, the cavity regions are primarily found in two main areas: within and near the Heart region, and enveloped by the Brain. In the results of masking-based methods, SpaMask failed to identify the cavity regions, while stCMGAE and MAEST only detected portions of the cavity regions. Specifically, stCMGAE only identified cavity regions near the Heart but missed those surrounding the Brain. Although MAEST identified both regions, it incorrectly merged some of the Heart-adjacent cavity regions with the Connective tissue. Only MMSpa accurately identified the cavity regions distributed across different areas. For other structures, MMSpa also precisely reconstructed their morphology and spatial distribution, aligning these structures with their corresponding marker gene expression patterns (Fig 5C).

Subsequently, we examined another E9.5 mouse embryo dataset (Slice #E9.5_E2_S3) containing 5,059 spots, 24,238 genes, and 13 distinct annotated regions (Figs 5D and S14A). By setting the cluster number to 13, we performed a quantitative comparison. MMSpa achieved the highest ARI score of 0.499 and the highest mean score across all three metrics (S14B Fig). Additionally, MMSpa effectively identified a majority of fine-grained annotation regions and accurately aligned them with their corresponding marker gene expression, including the Brain (domain 1 and 2), Lung primordium (domain 3), Mesenchyme (domain 11), Cavity (domain 8), Heart (domain 13), Sclerotome (domain 7), and Branchial arch (domain 9) (Fig 5D and 5E). In particular, MMSpa accurately identified the Telencephalon region (domain 1) within the Brain (Fig 5E). This finding is consistent with previous analysis of the E9.5 dataset (Slice #E9.5_E1_S1). Notably, the Telencephalon region was only successfully captured by MMSpa (Figs 5E and S14A). Moreover, by setting the cluster number to 16, we obtained more detailed, finer-grained structure results (Figs 5E and S14C). However, despite this increase in the number of clusters, other methods still exhibited unsatisfactory performance in fine-grained identification. For instance, the results from SpaMask were still fragmented, with the same clusters dispersed throughout the tissue. MAEST and stCMGAE failed to accurately identify fine-grained structures. In particular, although both methods identified most of the Brain region, they incorrectly assigned a part of the Brain region, located in the top-left corner of the slice, to the Spinal cord region (Fig 5E). Additionally, MAEST erroneously merged the Liver with the Lung region, and stCMGAE incorrectly combined the Branchial arch with the Mesenchyme region. In contrast, MMSpa, which is also based on masking strategies, successfully identified important fine-grained structures and matched them with the annotated structures (Figs 5F and S14D). Specifically, MMSpa accurately distinguished between the nearby Heart and Liver regions, as well as correctly identifying a clean and intact Brain and Spinal Cord region. These results demonstrate that MMSpa outperforms existing masking-based methods in terms of fine-grained structure prediction, providing a more comprehensive domain identification result that addresses the limitations of current approaches. Furthermore, MMSpa also shows a strong advantage over non-masking-based methods. For example, compared to MMSpa, conST obtained noisy results with unclear boundaries, while GraphST, and SpaceFlow failed to identify the Liver region. SEDR and STAGATE did not identify the Cavity region near the Lung primordium, with their Branchial arch results poorly matching the annotated structures (S14C Fig).

In conclusion, MMSpa showed continuous spatial domains and preserved tissue architecture, with clearly defined domain boundaries in both E9.5 datasets. These results also highlight the robustness of MMSpa. Despite the inherent biological variation between embryos, MMSpa not only consistently identifies similar spatial structures but also detects domain differences between biological samples at the same developmental stage (E9.5). For example, MMSpa successfully identified the Spinal cord in the #E9.5_E2S3s dataset, which was absent in the #E9.5_E1_S1 dataset (Fig 5A and 5F). These results demonstrated that MMSpa can generalize data applications and has significant advantages in complex structure fine-grained identification. This capability offers new opportunities for analyzing complex biological structures and understanding developmental processes.

### MMSpa improves spatial domain identification from high-resolution ST datasets across various spatial technologies

We further applied MMSpa to high-resolution ST datasets from different spatial platforms to demonstrate the scalability of MMSpa.

**Fig 6:**
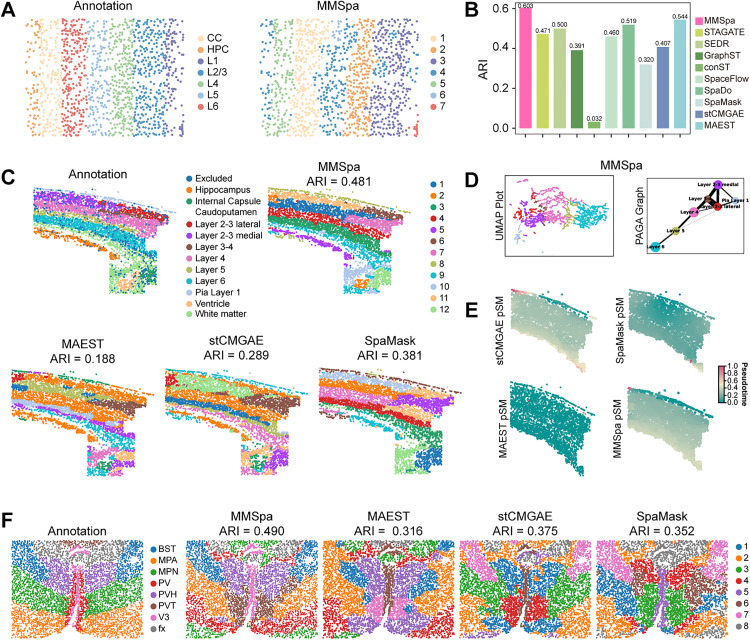
MMSpa improves spatial domain identification and downstream analysis on STARmap, osmFISH, and MERFISH ST datasets. **(A)** Visualization of manual annotations (left) and MMSpa’s domain identification results (right) for the STARmap dataset. **(B)** The bar plot shows the quantitative performance in the STARmap dataset. The y-axis represents the ARI score. **(C)** Visualization of manual annotations and domain identification results of MMSpa, MAEST, stCMGAE, and SpaMask for osmFISH dataset. **(D)** UMAP visualization and PAGA trajectory graph of MMSpa for osmFISH dataset. Results for other methods are provided in S15D Fig. **(E)** Pseudo-Spatiotemporal Map (pSM) of MMSpa, MAEST, stCMGAE, and SpaMask for osmFISH dataset. Results for other methods are provided in S15D Fig. **(F)** Visualization of manual annotations and domain identification results of MMSpa, MAEST, stCMGAE, and SpaMask for MERFISH ST dataset. The underlying data for this figure can be found at https://doi.org/10.5281/zenodo.17451775.

First, we tested whether MMSpa could be applied to a high-plex RNA imaging-based ST data from the STARmap platform [3] with single-cell resolution from mouse visual cortex tissue (S1 Table), which had been manually annotated into seven distinct structure layers (Fig 6A). Using these manual annotations as the ground truth, we compared the performance of MMSpa with seven other methods. Notably, MMSpa outperformed all other methods, achieving the highest domain identification accuracy with an ARI score of 0.603, an NMI score of 0.683, and a Purity score of 0.753 (Figs 6B and S15A). In contrast, the other methods recorded ARI scores below 0.544, with MAEST achieving the second-highest accuracy. Furthermore, we utilized the latent representations generated by these methods to explore the developmental trajectory of the mouse cortex, spanning from Layer 1 (L1) to Layer 6 (L6), using UMAP, PAGA analysis, and pSM computation (S15B Fig). We found that only SpaDo, and the masking-based methods (MMSpa, SpaMask, MAEST, and stCMGAE) exhibited clear separation of cells across different layers in the UMAP visualization, captured the developmental trajectory from L1 to L6 in PAGA analysis, and displayed a gradient of color becoming lighter from right to left in the pSM analysis, reflecting a continuous developmental progression. Next, we extended our analysis to another mouse cortex ST dataset, generated using the osmFISH technique [4], which provides coverage of 162 UMIs per bead. This mouse somatosensory cortex ST dataset was characterized by a typical multi-layered cortical structure and was manually annotated into 12 distinct spatial domains (Figs 6C and S15C). MMSpa reduced noise within each identified layer, with each cluster focused in the same location and distinct color blocks. In contrast, other masking-based methods MAEST, stCMGAE, and SpaMask misidentified the same clusters across different layers (Fig 6C). For instance, Cluster 2 from MAEST and stCMGAE, and Clusters 6 and 11 from SpaMask, were incorrectly placed across multiple layers. We further analyzed the latent representations obtained from existing methods, focusing on the spots corresponding to L1 to L6. We found that only MMSpa, stCMGAE, SpaMask, and SpaDo effectively separated the spots from L1 to L6, accurately capturing the continuous developmental trajectory across cortical layers (Figs 6D and S15D). However, only MMSpa’s pSM analysis revealed a smooth gradient of color, indicating a progressive transition between neighboring layers, while the other methods either showed one color dominating or an anomalous jump (Figs 6E and S15D).

Finally, we applied MMSpa to a mouse hypothalamus ST dataset generated using the MERFISH technique [5]. Remarkably, compared to other methods, MMSpa achieved a 19.1% higher clustering accuracy of ARI, a 12.2% higher NMI, and an 18.0% higher Purity, exhibiting significantly enhanced delineation of tissue structures (Figs 6F and S15E). In addition to the improved quantitative metrics, MMSpa demonstrated clear advantages in the recovery of biological structural details compared to other masking-based methods. Specifically, stCMGAR, SpaMask, and MAEST were unable to simultaneously identify the BST, MPN, PVH, and V3 regions. In contrast, MMSpa not only accurately restored the larger BST, MPN, and PVH regions but also successfully captured the shape and position of the smaller V3 region (Fig 6F).

Overall, the results above demonstrate the scalability of MMSpa across multiple spatial transcriptomics datasets from various spatial technologies. Both domain identification results and downstream analysis highlight MMSpa’s competitiveness over other methods, providing biologically relevant insights that align more closely with known biological developmental patterns.

### Extension application for MMSpa to identify spatial domains in multiple spatial slices

We conducted an extension application for MMSpa to identify spatial domains in multiple spatial slices. We performed additional preprocessing on the input data. Specifically, assume there are k slices to be processed for the multi-slice domain identification task. First, we denote the gene expression matrices for each slice as $X_{\text{1}}$ to $X_{k}$, and their spatial adjacency matrices as $A_{\text{1}}$ to $A_{k}$. We then concatenate the k gene expression matrices along the spot dimension to obtain a joint gene expression matrix X:


$$X=[\begin{array}{c} X_{\text{1}}\\ \vdots \\ X_{k} \end{array}]$$

Similarly, we create a block-diagonal adjacency matrix A from the k spatial adjacency matrices:


$$A=[\begin{array}{ccc} A_{\text{1}} & \cdots & \text{0}\\ \vdots & \ddots & \vdots \\ \text{0} & \cdots & A_{k} \end{array}]$$

Finally, we use X and A as inputs for MMSpa to perform domain identification across the multi-slice dataset.

We applied MMSpa for multi-slice domain identification on the DLPFC dataset. The DLPFC dataset consists of 12 slices from 3 adult samples, with 4 adjacent slices per sample:

- Sample1 with #151673, #151674, #151675, and # 151676.
- Sample2 with #151669, #151670 #151671, and # 151672.
- Sample3 with #151507, #151508 #151509, and # 151510.

We performed multi-slice domain identification for each of the 3 sample groups. Among the methods we compared for single-slice domain identification, considering that SpaDo was initially designed for multi-slice domain identification, and GraphST, STAGATE, and SpaMask have also mentioned that they can be extended for multi-slice domain identification. We compared the performance of MMSpa in multi-slice domain identification with these methods by calculating domain identification accuracy metrics: ARI, NMI, and Purity (S5 Table).

As shown in the results, MMSpa demonstrates the ability to identify spatial domains in multiple spatial slices. Specifically, for Sample 1, MMSpa outperforms the other methods in all three metrics. In the application of Sample 2, although MMSpa is lower than STAGATE by 2.7% in the NMI metric, it still outperforms in both ARI and Purity. For Sample 3, MMSpa maintains the highest accuracy in the Purity metric, with only a minor 0.5% difference in NMI compared to STAGATE. Notably, MMSpa and STAGATE show similar performance in the other two samples, with MMSpa performing slightly better. As for the ARI value, although the ARI value of MMSpa is slightly lower than SpaDo’s, it is worth noting that SpaDo’s better ARI performance in Sample 3 does not extend to the other samples. MMSpa outperforms SpaDo on all three metrics for the other two samples, with the largest accuracy difference reaching 11.7% (e.g., for Sample 1, MMSpa ARI = 0.590, SpaDo ARI = 0.473).

### Ablation studies

To verify the contributions of the main components in MMSpa, we conduct several ablation studies on six datasets with manual annotations from five platforms, including 10× Visium datasets (the DLPFC dataset with 12 slices and human breast cancer dataset), the Stereo-Seq dataset (E9.5 mouse embryo dataset from slice #E9.5_E2_S3), the STARmap mouse cortex dataset, the osmFISH mouse somatosensory cortex dataset, and the MERFISH mouse hypothalamus dataset.

As shown in S16 Fig, we first tested whether the performance of MMSpa benefited from the edge removal strategy (S1 Note). The results show that the enhanced spatial graph provides a significant advantage over the initial spatial graph in each platform dataset application, with an absolute improvement of 3.6%–8.5% in ARI, 1.7%–5.8% in NMI, and 1.8%–3.6% in Purity of the six datasets. Considering that the DLPFC dataset includes 12 slices, we conducted a slice-wise analysis and observed that each slice’s three metrics improved to varying degrees (S2 Table). These improvements demonstrated the importance of the edge removal strategy in each platform dataset application, which plays a crucial role in enhancing the specificity of the spatial graph for the domain identification task. Particularly, in the application to the human breast cancer dataset, the median scores of the three metrics (ARI, NMI, and Purity) increased by 8.0%, 2.1%, and 3.4%, respectively, demonstrating the utility of this edge removal strategy in the identification of tissue domains with heterogeneous cell types (S16 Fig).

We then examined the influence of the masking strategies (S1 Note). MMSpa employs two masking strategies: one that masks the gene expression matrix before the encoder (masked feature reconstruction), and another that re-masks the encoder’s output before the decoder. Removing the masking of the gene expression matrix before the encoder means that the model reconstructs the entire gene expression matrix. Our results showed that the accuracy of the six datasets from five platforms dropped by 6.0%–14.4% in ARI, 2.1%–13.4% in NMI, and 2.7%–8.5% in Purity when the gene expression matrix was not masked before the encoder (S16 Fig). This suggests that the denoising (masked) graph autoencoder framework with masked feature reconstruction significantly enhances domain identification performance in ST datasets with different resolutions. The improvement in domain identification accuracy for each 12 slices of the DLPFC dataset further supports this conclusion (S2 Table). For the re-mask strategy, the removal of this strategy caused various degrees of decrease in accuracy in each dataset from the five platforms. In particular, in the application of imaging-based platforms (STARmap, osmFISH, and MERFISH) ST datasets, the ARI, NMI, and Purity decreased by 2.0% to 10.9%, 1.0%–9.4%, and 2.6%–4.6%, respectively (S16 Fig). A similar trend could be found in each 12 slices of the DLPFC dataset (S2 Table), causing the median ARI, NMI, Purity scores of the DLPFC dataset decreased by 10.0%, 7.4%, and 4.9%, respectively (S16 Fig). The re-mask strategy can be regarded as a form of regulation, which can further encourage the encoder to learn the latent representations.

Moreover, we investigated the impact of the edge removal strategy and masked feature reconstruction on the downstream analysis (UMAP, trajectory inference, and pSM analysis) of latent representations. We conducted the ablation studies on slices 151674, 151672, and 151508 of the DLPFC dataset. Compared to the edge removal strategy (S17A Fig), the masked feature reconstruction strategy had a more substantial impact on downstream analyses (S17B Fig). After removing the masked feature reconstruction strategy, all three downstream analyses were affected to varying degrees, leading to a reduction in the ability of latent representations to capture biosignatures, with trajectory inference and pSM being the most impacted (S17B Fig). This suggests that stable latent representations encompass more biometric features, which not only improve domain identification performance but also significantly contribute to downstream analyses, while the masked feature reconstruction strategy is essential for extracting more stable and core information from ST data.

### Selection of parameters

We conducted sensitivity analyses for five parameters (S2 Note and S18A–S18D Fig), where three parameters (masking ratio, re-masking ratio, and lambda for SCE loss) for model framework, and two parameters (the number of neighbor spots for initial spatial graph construction, and the number of neighbor spots for opponent spatial graph construction) for spatial graph construction. Through parameter sensitivity analysis, we demonstrated the impact of different parameter values on the performance of the model:

*Masking ratio*: In most cases, when the masking ratio was set with a low value (e.g., 0.1 or 0.2), the domain identification accuracy was suboptimal. This could be related to the model not being challenging enough to capture useful features when the masking ratio is too low. Additionally, in most datasets, performance also decreased when the masking ratio exceeded 0.3. This might be related to the excessive information loss.

*Re-masking ratio*: The results for all three datasets show that the model performance is more stable when the re-masking ratio is below 0.3. It can be seen that the model performs best when the re-masking ratio is 0.1 in most cases.

*Lambda*: As lambda increases, it brings benefits to the model performance on complex biological structures. Furthermore, in general cases, a lambda set to 1 is sufficient to ensure the reconstruction loss. If lambda is set too large, it may cause the model to miss the optimal solution during training.

*The k_cutoff*: For spot resolution ST data, MMSpa performs optimally when k_cutoff is set to 6, while for single-cell resolution ST data, MMSpa demonstrates greater robustness to variations in k_cutoff values. This difference is likely due to the larger microsphere diameter in spot resolution sequencing.

*The exp_cutoff*: As the exp_cutoff value increases, the actual number of single cells in the spot resolution data increases significantly more than in the single-cell resolution data, leading to a broader range of performance fluctuations in the model under spot resolution.

Overall, we recommend setting the masking ratio to 0.3, the re-masking ratio to 0.1 or below 0.3, the lambda to 1 in general cases and lambda >1 in complex biological cases, the k_cutoff to 6 for ST datasets with spot resolution and to 6–12 that can be adjusted for other single-cell resolution ST datasets, and the exp_cutoff value to 300 as a general guideline, with a range of 300–450 that can be adjusted based on specific data characteristics.

Additionally, we showed the robustness of MMSpa (S3 Note and S18E and S19 Figs) and demonstrated the effectiveness of SCE loss in improving the domain identification accuracy of MMSpa (S2 Note and S18F Fig).

## Discussion

Traditional approaches for delineating tissue structures rely on manual annotation, which requires substantial human and material resources and introduces subjectivity. Developing computational methods for domain identification based on ST data provides an unbiased way to delineate tissue structures [10, 88]. Accurate identification of spatial domains is crucial for revealing spatial landscapes and exploring biological functions within complex tissues [18,71,89], such as visualizing anatomical structures [16], revealing spatial features of diseases and development [90,91], detecting domain-specific marker genes [17,92].

Domain identification, as a fundamental step in ST data analysis, directly affects downstream biological discovery, especially marker genes and pathways in specific domains or microenvironments that are closely related to their spatial localization [93]. Among the existing spatial domain identification methods, MMSpa simultaneously offers three key advantages: significantly improves domain identification accuracy, learns stable latent representations that capture essential biological details, and resolves previously obscured biological features across diverse tissue contexts. First, we have demonstrated that MMSpa achieved significant improvement in domain identification accuracy across 21 datasets from five different platforms. Even when all comparison methods employed the same clustering algorithm, MMSpa achieved the best domain identification performance in separating continuous layers, providing clear boundaries with less noise, and identifying the annotated layers, with the highest accuracy (S4 Note, S3 Table, and S20 Fig). Then, we have shown that MMSpa can learn stable latent representations with key biological details, thus enhancing the significance of downstream analyses, such as understanding the tissue biology of cancer [94]. By utilizing the latent representations from MMSpa, we were able to investigate the spatial continuity of changes from healthy regions to tumor edges and tumor areas. This type of analysis is clinically relevant, as strict boundary definitions are often absent in cancer tissues. Understanding such spatial continuity is vital for advancing cancer research and diagnostics. Finally, whether applied to healthy or diseased tissues, MMSpa uniquely identified functional regions that other methods overlook at finer scales. This capability directly enhances MMSpa’s potential for uncovering new biological insights, highlighting MMSpa’s biological value in practical applications. Particularly, MMSpa uniquely identified the important brain region VS associated with CSF secretion, and detected synaptic-related biological processes consistent with recent research findings. MMSpa also uniquely revealed a potential cancerous region that had not been highlighted in the original study due to its less prominent histopathological features and being consistently misclassified as normal tissue by other methods.

The key to the superior performance of MMSpa is attributed to four aspects. First, MMSpa constructs an enhanced spatial graph using the graph edge removal strategy. Constructing a spatial graph is a crucial and common step in most ST dataset analysis algorithms, particularly in domain identification, as it essentially provides the model with some prior information about the spatial domain. Traditional methods construct spatial graphs based solely on spatial proximity. However, it is difficult to ensure that the spots in the neighborhood obtained by the spatial proximity principle are all in the same domain, leading to traditional spatial graphs with noise, particularly around domain boundaries. MMSpa improves upon the traditional spatial graph approach by the edge removal strategy to mitigate such noise (S5 Note), which makes the enhanced spatial graph more targeted for the domain identification task. In fact, cells within the same domain always perform similar biological functions, which are inherently linked to gene expression. In other words, spots in the same domain are more likely to exhibit similar gene expression patterns. Although the degree of this similarity may not be precisely defined, we can be sure that when the expression of two spots differs sufficiently, they are more likely to be in different domains (S21 Fig). By utilizing gene expression distance to construct an opposed spatial graph, MMSpa enhances the characterization of spatial domain information at the input level (Figs 1 and S16). Second, MMSpa is a denoising (masked) graph autoencoder-based method that employs a masked feature reconstruction strategy. The key to existing graph autoencoder-based spatial domain identification methods is to obtain a good latent representation. However, a “good” representation is expected to capture stable structures and should be recoverable even from partial observation [95]. A simpler understanding is that humans are able to recognize partially masked or corrupted images based on some core information [22,95]. Denoising autoencoders, which corrupt partial input data and then attempt to reconstruct it, have been shown to make learned representations more robust [95]. Denoising autoencoders have found widespread applications in natural language processing [96,97] and computer vision [98], and could also be applicable to graph-based autoencoders [99–101]. MMSpa leverages the denoising (masked) graph autoencoder to emphasize core information inherent in the input ST dataset, enabling the learned representations to better reflect key biological features while reducing noise and improving performance in domain identification and downstream analysis tasks (S16 and S17 Figs). In contrast, existing graph autoencoder-based spatial domain identification methods typically employ the vanilla architecture, where the entire gene expression is reconstructed during training. As a result, their latent representations often lack crucial biological information. Third, MMSpa utilizes a re-mask strategy before feeding the encoder’s output embeddings into the decoder. This can be regarded as a form of regularization, similar to “dropout”, enabling MMSpa to tackle more training challenges and encouraging the encoder to learn the latent representations (S16 Fig). Finally, MMSpa is built upon the GAT autoencoder model [102], which can adaptively learn similarities between different spots by considering their local neighborhood context. Notably, the GAT layer, which only computes the weight of spots in the neighborhood, can also be considered as a form of mask attention strategy [103]. However, we acknowledge that, similar to other spatial domain identification GNN-based methods, MMSpa currently faces a limitation in accurately determining the number of clusters without prior knowledge. To address this, we recommend selecting the number of clusters by identifying the maximum score within a range of potential values, using metrics such as the Silhouette score [34] or other clustering evaluation measures. Additionally, it is worth noting that recent advances in the field of statistics, nonparametric Bayesian [104], such as the nonparametric Potts prior, offer promising alternatives for inferring spatial domains in a fully data-driven manner [105]. Future work could explore integrating these adaptive priors into our framework to further enhance MMSpa’s generalizability.

While there are similarities in the GAT autoencoder framework between MMSpa and STAGATE, we highlight several differences between the methods. As mentioned above, MMSpa is a denoising (masked) GAT autoencoder framework that employs masked gene expression reconstruction, whereas STAGATE reconstructs all gene expressions. MMSpa not only adapts to learn the weights from each neighboring spot but also focuses on learning more stable hidden representations that capture core biological information. MMSpa also differs from STAGATE in that it incorporates the spatial graph edge removal strategy (See “Methods” and S5 Note), which enhances its specificity for domain identification at the input level. Additionally, MMSpa utilizes a cosine error loss function instead of the mean square error (MSE) used by STAGATE. While MSE emphasizes absolute errors and being sensitive to dimensionality [106], it can be adversely affected by outliers and extreme values, leading to a total loss close to zero, but may not be enough for training. In contrast, the cosine error loss focuses on the degree of similarity between vectors rather than values [107] (See “Methods”), making MMSpa more suitable for high-dimensional and sparse ST gene expression datasets. Furthermore, although SEDR employs a similar masking strategy to MMSpa, it combines fully connected layers with variational autoencoders and performs dimensionality reduction twice during both the input and training process, which may lead to reduced interpretability and potential loss of information.

Lastly, for recently developed methods: stCMGAE [27], SpaMask [26], MAEST [25], and m2ST [108], which are based on the same masking strategies as MMSpa, we provided a comprehensive methodological comparison of MMSpa and them from technical differences, advantages, and disadvantages (S6 Note and S4 Table). MMSpa stands out in its biologically driven edge removal strategy for spatial graph enhancement, dynamic self-attention mechanism for fine-grained sub-domains identification, and streamlined but robust autoencoder frame. More importantly, MMSpa can solve the problem that in applications of highly heterogeneous or highly complex tissues, existing domain identification methods always have unclear domain boundary characterization and inadequate identification of fine-grained sub-domains. The edge removal strategy of MMSpa fundamentally addresses the issue of noisy edges in the spatial graph, significantly enhancing the clarity of domain boundary delineation in highly heterogeneous or highly complex tissues. The combination of the dynamic self-attention mechanism with the masking strategies enables MMSpa to better capture the deeper and core biological features embedded in highly sparse ST datasets, offering significant advantages in identifying fine-grained sub-domains in applications with high tissue heterogeneity and complexity. As demonstrated in higher accuracy, enhanced detection of complex biological structures, methodological innovation, and greater potential for biological discoveries, we believe that the improvements of MMSpa over existing masking-based methods are both clear and significant.

Both the spatial graph edge removal strategy and the masked feature reconstruction strategy offer novel perspectives on ST data analysis. Perhaps some reverse thinking and a subtractive approach to the model can yield unexpected results. While the present version of MMSpa focuses on identifying spatial domains from single-slice ST datasets, our preliminary investigations demonstrate its inherent adaptability to multi-slice analyses. Despite using the relatively simple data preprocessing in multi-slice domain identification, MMSpa can still identify spatial domains in multiple spatial slices, and it remains highly competitive in its performance. In our forthcoming research, we may focus on incorporating a robust ST slices integration module to enhance the scalability of MMSpa and further explore new biological discoveries. As ST datasets become increasingly prevalent, we anticipate that effective methods like MMSpa, which integrates multiple data types and extracts useful biological information, will serve as valuable tools for ST data analysis, offering significant potential for downstream analytical tasks.

## Methods

### Data collection and preprocessing

We applied MMSpa to 22 ST datasets generated from the 10× Visium [1], Stereo-seq [2], STARmap [3], osmFISH [4], and MERFISH [5] platforms. More details of all used datasets can be found in S1 Table. The raw gene expressions in all datasets were normalized by a scale factor (10,000 by default) and log-transformed. Finally, 3,000 highly variable genes were selected as inputs to MMSpa and used for spatial graph construction.

### Spatial graph construction with edge removal strategy

Given the gene expressions and spatial coordinates, the spatial graph is constructed in three steps.

First, construct the initial spatial graph. Calculate the Euclidean distance between spots by using their spatial coordinates, and select b neighbor spots for each spot through the spatial proximity principle. For spot i, if spot j is one of its neighbors, then there exists a directed edge from spot i to spot j on the initial spatial graph. Let B be the adjacency matrix of the initial spatial graph, that means $B_{ij}=\text{1}$, otherwise, $B_{ij}=\text{0}$.

Then, construct an opponent spatial graph. Calculate the Euclidean distance between spots by using their gene expressions. For each spot, select c spots that are furthest from it. For spot i, if spot j is one of the farthest spots from spot i, then, there exists a directed edge from spot i to spot j on the opponent spatial graph. Let C be the adjacency matrix of the opponent spatial graph, that means $C_{ij}=\text{1}$, otherwise, $C_{ij}=\text{0}$.

Finally, the edges in the initial spatial graph where the initial spatial graph coincides with the opponent spatial graph are considered noisy edges. Remove the noisy edges from the initial spatial graph to get the final enhanced spatial graph. Let A be the adjacency matrix of the final spatial graph, that means if $B_{ij}>\text{0}$, $A_{ij}=B_{ij}-C_{ij}$, otherwise, $A_{ij}=\text{0}$.

### The MMSpa framework for latent representation learning

Given the gene expression matrix X with n spots and m genes (here we default to select 3,000 highly variable genes for input and m defaults to 3,000). Let V be the set of all n spots, $A\in {\left\{\text{0},\text{1}\right\}}^{n\times n}$ be the adjacency matrix of the spatial graph. The MMSpa framework consists of four main parts: mask strategy before encoder, encoder, re-mask strategy before decoder, and decoder.

We begin by selecting a subset of spots through random sampling with uniform probability, without replacement. Let $V_{sub}$ be the sampled subset of spots, $V_{sub}\subset V$. Then, for each spot $i\in V_{sub}$, use a learnable vector $x_{\left[i\right]}\in R^{m}$ to mask its gene expressions $x_{i}\in R^{m}$. Thus, the masked gene expression matrix $\tilde{X}$ can be defined as:


$$\tilde{X}=\left\{ \begin{array}{cc} x_{i}, & if\;i\in V\;and\;i\notin V_{sub}\\ x_{\left[i\right]}, & if\;i\in V_{sub} \end{array},\right.\;$$ (1)

The encoder takes the masked gene expression matrix $\tilde{X}$ and the adjacency matrix A of the spatial graph as inputs. Specifically, we utilize a GAT as the encoder to learn a latent representation $H_{i}$ for spot i, since GAT can flexibly aggregate the information from spots’ neighborhoods. Let L be the number of layers of the encoder, $d_{l}$ be the number of l-th (l∈{1,2,…,L}) layer’s output dimension, and $N_{i}$ be the set of neighborhoods of spot i (including spot i itself) according to the adjacency matrix A. Formally, the l-th layer representation for spot i (∀i∈{1,2,…,n}) produced by the encoder can be expressed as:


$$H_{i}^{\left(l\right)}=\sigma \left(\sum {\mathrm{\backslash nolimits}}_{j\in N_{i}}\alpha_{ij}^{\left(l\right)}\left(W_{e}^{\left(l\right)}H_{i}^{\left(l-\text{1}\right)}\right)\right),$$ (2)

where $W_{e}^{\left(l\right)}$ denotes the trainable weight matrix, $W_{e}^{\left(l\right)}\in R^{d_{l}\times d_{l-\text{1}}}$, $d_{\text{0}}=m$, σ is the activation function, and $H^{\left(\text{0}\right)}=\tilde{X}$. The $\alpha_{ij}^{\left(l\right)}$ denotes the weight between spot i and spot j ($j\in N_{i}$) in the l-th layer by the self-attention mechanism [102,103]. We follow Velickovic and colleagues [103] and $\alpha_{ij}^{\left(l\right)}$ can be expressed as:


$$\alpha_{ij}^{\left(l\right)}={\text{Softmax}}_{\text{j}}\left(e_{ij}^{\left(l\right)}\right)=\frac{\text{exp}\left(e_{ij}^{\left(l\right)}\right)}{\sum_{t\in N_{i}}\text{exp}\left(e_{it}^{\left(l\right)}\right)},$$ (3)

$$e_{ij}^{\left(l\right)}=\text{Sigmoid}\left(r_{s}^{{\left(l\right)}^{T}}\left(W_{e}^{\left(l\right)}H_{i}^{\left(l-\text{1}\right)}\right)+r_{v}^{{\left(l\right)}^{T}}\left(W_{e}^{\left(l\right)}H_{j}^{\left(l-\text{1}\right)}\right)\right),$$ (4)

where $r_{s}^{{\left(l\right)}^{T}}$ and $r_{v}^{{\left(l\right)}^{T}}$ are the trainable weight vectors, if $H_{i}^{\left(l\right)}\in R^{d_{l}}$, $r_{v}^{\left(l\right)},r_{s}^{\left(l\right)}\in R^{d_{l}}$.

We denote ${H=H}^{\left(L\right)}$ as the final output of the encoder, where $h_{i}$ denotes the latent representation of spot i.

Before decoding, we employ a re-mask strategy to process H. Specifically, we again randomly sample a subset of spots $V_{sub}^{\prime }$ from V, $V_{sub}^{\prime }\subset V$. Then, for each spot $i\in V_{sub}^{\prime }$, we use a learnable vector $h_{\left[i\right]}\in R^{d_{L}}$ to replace $h_{i}\in R^{d_{L}}$ on the masked node indices of H. Thus, the masked encoder output $\tilde{H}$ can be defined as:


$$\tilde{H}=\left\{ \begin{array}{cc} h_{i}, & if\;i\in V\;and\;i\notin V_{sub}^{\prime }\\ h_{\left[i\right]}, & if\;i\in V_{sub}^{\prime } \end{array}\right.\;,$$ (5)

It should be noticed that MMSpa employs two independent masking operations, both based on all spots. For each masking, a corresponding number of spots is randomly selected for feature masking, determined by the masking and re-masking ratios. As a result, some spots may be masked twice, but this overlap does not impact model performance. The first masking operation aims to make the model more challenging in reconstructing gene expression, while the second re-masking strategy serves as a form of “dropout” before the decoder, acting as a regularization technique during model training.

After that, the masked encoder output $\tilde{H}$ is fed into the decoder to reconstruct each masked spot’s gene expressions. The decoder adopts a symmetric architecture with the encoder. Specifically, the number of layers of the decoder is the same as the encoder. If $d_{l}^{\prime }$ is the number of l-th (l∈{1,2,…,L}) layer’s output dimension in decoder, $d_{l}^{\prime }=d_{L-l}$. Then, the embedding of spot i in layer k by the decoder can be expressed as:


$$Z_{i}^{\left(l\right)}=\sigma \left(\sum {\mathrm{\backslash nolimits}}_{j\in N_{i}}\alpha_{ij}^{\prime (l)}\left(W_{d}^{\left(l\right)}Z_{i}^{\left(l-\text{1}\right)}\right)\right),$$ (6)

where $W_{d}^{\left(l\right)}$ denotes the trainable weight matrix, $W_{d}^{\left(l\right)}\in R^{d_{l}^{\prime }\times d_{l-\text{1}}^{\prime }}$, $d_{\text{0}}^{\prime }=d_{L}$, $Z^{\left(\text{0}\right)}=\tilde{H}$. Similarly, $\alpha_{ij}^{\prime (l)}$ can be expressed as:


$$\alpha_{ij}^{\prime (l)}={\text{Softmax}}_{\text{j}}\left(e_{ij}^{\prime (l)}\right)=\frac{\text{exp}\left(e_{ij}^{\prime (l)}\right)}{\sum_{t\in N_{i}}\text{exp}\left(e_{it}^{\prime (l)}\right)},$$ (7)

$$e_{ij}^{\prime (l)}=\text{Sigmoid}\left(r_{s}^{{\prime (l)}^{T}}\left(W_{d}^{\left(l\right)}Z_{i}^{\left(l-\text{1}\right)}\right)+r_{v}^{\prime {\left(l\right)}^{T}}\left(W_{d}^{\left(l\right)}Z_{j}^{\left(l-\text{1}\right)}\right)\right),$$ (8)

where ${r^{\prime }}_{s}^{{\left(l\right)}^{T}}$ and $r_{v}^{{\prime (l)}^{T}}$ are the trainable weight vectors. The ${Z=Z}^{\left(L\right)}$ is decoder’s final output, and $z_{i}$ denotes the embedding of spot i.

Finally, in the GAT decoder, a masked spot during the gene expression masking step is compelled to reconstruct its original gene expression using the unmasked neighboring latent representations (see “Discussion”). To assess the quality of this reconstruction, we employ the cosine error, and the loss function that MMSpa aims to minimize can be formulated as:


$$\frac{\text{1}}{\left|V_{sub}\right|}\sum {\mathrm{\backslash nolimits}}_{{i\in V}_{sub}}{\left(\text{1}-\frac{x_{i}^{T}z_{i}}{\left\|x_{i}\right\|\cdot \left\|z_{i}\right\|}\right)}^{\lambda },\;\;\;\;\lambda \geq \text{1}.$$ (9)

Here, we average the loss of all masked spots as the total loss and introduce a hyperparameter λ (λ≥1) to balance the degree of reconstruction for different spots. The hyperparameter λ acts as a scaling strategy to modulate the contribution of each spot based on reconstruction difficulty. Specifically, λ is employed to down-weight the impact of spots that are easily reconstructed, thus directing more focus towards those that are challenging to reconstruct. During training, some spots are inherently easier to reconstruct, characterized by the fact that their reconstructed features are closer to the true gene expression values. In fact, for these easy spots, their cosine similarity will be relatively closer to 1. When λ>1, it can lead to the reconstruction error of these easily reconstructed spots to decay faster to 0. Meanwhile, it can also increase the reconstruction error values for harder-to-reconstruct spots, amplifying their impact on the total reconstruction error. This type of error is widely used in object detection, known as focal loss [107].

The parameters, device models used, and the running time of MMSpa can be found in S6 and S7 Tables.

### Spatial domain identification and downstream analyses

Based on MMSpa latent representations, we employed different algorithms for domain identification and the following downstream analyses.

For domain identification, we utilized the mclust algorithm [109] from the R package mclust version 6.0.1. We set the cluster number for tissue slices with manual annotation to align with the ground truth. When there is no manual annotation, we test different cluster counts and select the count that gives the highest Silhouette score [34]. Specifically, for the application of two brain datasets (the sagittal posterior and coronal), we set the cluster numbers to range from 20 to 25. For each dataset, we performed clustering at different cluster numbers and computed the corresponding Silhouette scores for MMSpa clustering results. Finally, we selected the cluster number that yielded the highest Silhouette score for each dataset. In the sagittal posterior dataset, the cluster number corresponding to the highest Silhouette score was 24, while in the coronal dataset, the optimal cluster number was 23.

We used the *FindAllMarkers()* function from the R package Seurat version 5.0.1 [110] to identify DEGs within each domain. For other downstream analyses (UMAP, PAGA graph, and pSM computation), we employed the Python package SCANPY version 1.10.1 [111]. In the case of the DLPFC dataset, the root spot for the pSM was defined as the spot in the WM state. For the human breast cancer dataset, the root spot was defined as the spot in the Healthy state. For the mouse visual cortex STARmap dataset, the root spot was defined as the spot in the L1 state. For the mouse somatosensory cortex osmFISH data, the root spot was defined as the spot in the Pia Layer 1 state.

### Comparison of MMSpa with existing domain identification methods

First, to evaluate the accuracy of MMSpa for domain identification, we quantitatively compared its performance against nine existing methods using various ST datasets with annotated labels. The methods included SpaceFlow [20], STAGATE [24], conST [21], GraphST [22], SEDR [23], stCMGAE [27], SpaMask [26], SpaDo [28], and MAEST [25] each applied with default parameters (S7 Note). We utilized three quantitative access metrics: ARI [112], NMI [113], and Purity [114].

For mouse brain datasets lacking specific annotation labels, we downloaded publicly available annotated atlas images from the Allen Brain Atlas website [115] as a reference to compare domain identification results across different methods. A similar comparison approach was also adopted in the study by Yuan and colleagues [65], which we followed here. Additionally, we use the SC [34] and DB [35] index as internal metrics to assess cluster separability and quantify the extent to which MMSpa clearly defines anatomical regions compared to other existing methods. Specifically, for methods that provide low-dimensional representations, we calculate the SC and DB index based on their low-dimensional embeddings. For methods that do not output low-dimensional representations, we compute these indices based on the ST gene expressions.

Second, for downstream analyses, we used the latent embeddings from each deep learning method for UMAP visualization, cell trajectory analysis (PAGA), and pSM computation.

## Supporting information

S1 Fig Benchmarking MMSpa with existing methods in the human DLPFC dataset for slice 151674. **(A)** Bar plots show the quantitative performance of MMSpa and nine other methods in domain identification accuracy across all 12 DLPFC tissue slices. The y-axis of each bar plot represents the NMI and Purity scores, respectively. **(B)** shows UMAP visualization, PAGA trajectory graph, and Pseudo-Spatiotemporal Map (pSM) generated by GraphST, SEDR, STAGATE, SpaceFlow, and SpaDo. The underlying data for this figure can be found at https://doi.org/10.5281/zenodo.17451775.
(TIF)

S2 Fig The domain identification results of each method for slices 151507, 151509, and 151510 are visualized and compared with the manual annotation results. The underlying data for this figure can be found at https://doi.org/10.5281/zenodo.17451775.
(TIF)

S3 Fig The domain identification results of each method for slices 151669, 151670, and 151671 are visualized and compared with the manual annotation results. The underlying data for this figure can be found at https://doi.org/10.5281/zenodo.17451775.
(TIF)

S4 Fig The domain identification results of each method for slices 151673, 151675, and 151676 are visualized and compared with the manual annotation results. The underlying data for this figure can be found at https://doi.org/10.5281/zenodo.17451775.
(TIF)

S5 Fig Benchmarking MMSpa with existing methods in the human DLPFC dataset for slice 151672, including the visualization of domain identification, UMAP, PAGA trajectory graph, and Pseudo-Spatiotemporal Map (pSM). The underlying data for this figure can be found at https://doi.org/10.5281/zenodo.17451775.
(TIF)

S6 Fig Benchmarking MMSpa with existing methods in the human DLPFC dataset for slice 151508, including the visualization of domain identification, UMAP, PAGA trajectory graph, and Pseudo-Spatiotemporal Map (pSM). The underlying data for this figure can be found at https://doi.org/10.5281/zenodo.17451775.
(TIF)

S7 Fig Visualization of manual annotations for slices and robustness test of MMSpa and existing methods. **(A)** Visualization of manual annotations for slices 151508 and 151672. **(B)** ARI boxplot for 6 methods on DLPFC datasets with different *K* (number of nearest neighbors). The underlying data for this figure can be found at https://doi.org/10.5281/zenodo.17451775.
(TIF)

S8 Fig MMSpa demonstrated superior identification capabilities on the mouse brain sagittal posterior and coronal datasets. **(A)** Histological image of the mouse brain sagittal posterior ST dataset. **(B)** Visualization of domain identification results for the sagittal posterior using SpaDo, SpaceFlow, GraphST, SEDR, STAGATE, and conST. **(C)** Top marker genes of the identified VS region in MMSpa (domain 16) on the mouse brain sagittal posterior ST dataset (above) and another sagittal posterior replicate (below). **(D)** Histological image of the mouse brain coronal ST dataset, and visualization of domain identification results for the coronal using SpaDo, SpaceFlow, GraphST, SEDR, STAGATE, and conST. **(E)** Bar plots of the top-left panel, top-right panel, bottom-left panel, and bottom-right panel show the Silhouette Coefficient for the sagittal posterior, the Davies–Bouldin index for the sagittal posterior, the Silhouette Coefficient for the coronal section, and the Davies–Bouldin index for the coronal section, respectively. **(F)** Comparison of MMSpa and other methods in distinguishing different regions of the sagittal posterior. The x-axis represents different methods, while the y-axis corresponds to regions of the sagittal posterior as detailed in Fig 3B. Points on the plot indicate whether each method identified the corresponding region, with size reflecting the score (1 for detection, 0 for non-detection). **(G)** Comparison of MMSpa and other methods in distinguishing different regions of the coronal. The x-axis represents different methods, while the y-axis corresponds to regions of the coronal as detailed in Fig 3E. Points on the plot indicate whether each method identified the corresponding region, with size reflecting the score (1 for detection, 0 for non-detection). **(H)** The GO: BP terms for domains 2 and 3 of MMSpa on the mouse brain coronal ST dataset. The underlying data for this figure can be found at https://doi.org/10.5281/zenodo.17451775.
(TIF)

S9 Fig Domain identification results on the mouse brain sagittal posterior ST dataset from competing methods by adjusting their clustering parameters between 20 and 25. The underlying data for this figure can be found at https://doi.org/10.5281/zenodo.17451775.
(TIF)

S10 Fig Domain identification results on the mouse brain sagittal posterior ST dataset from competing methods by adjusting their clustering parameters between 20 and 25. The underlying data for this figure can be found at https://doi.org/10.5281/zenodo.17451775.
(TIF)

S11 Fig Comparison of MMSpa and other methods on the anterior mouse brain ST dataset. **(A)** Histological image of the anterior mouse brain ST dataset. **(B)** Visualization of manual annotations for the anterior mouse brain. **(C)** Visualization of domain identification results for the anterior mouse brain obtained by MMSpa and nine other methods. **(D)** Bar plots show the quantitative performance of MMSpa and nine other methods in domain identification accuracy. The y-axis of each bar plot represents the ARI, NMI, and Purity metrics, respectively. The underlying data for this figure can be found at https://doi.org/10.5281/zenodo.17451775.
(TIF)

S12 Fig Comparative analysis of MMSpa with other methods on the human breast cancer and PDAC ST datasets. **(A)** Histological image of the human breast cancer slice. **(B)** Performance comparison of MMSpa against nine other existing methods, with asterisks indicating the top-performing method for each metric. **(C)** Visualization of domain identification results for the human breast cancer dataset using existing methods. **(D)** Spatial distribution of the luminal cells mapped by the CARD algorithm. **(E)** Pseudo-Spatiotemporal Maps (pSMs) generated by the nine compared methods. **(F)** Expression of the MALAT1 gene across the human breast cancer slice data. **(G)** The visualization results for SpaceFlow, conST, SpaDo, and GraphST. The underlying data for this figure can be found at https://doi.org/10.5281/zenodo.17451775.
(TIF)

S13 Fig Comparative analysis of MMSpa with other methods on the first applied E9.5 embryo Stereo-seq dataset (Slice #E9.5_E1_S1). **(A)** Visualization of spatial domains identified by nine existing methods with 12 domains. **(B)** Performance comparison of MMSpa against nine other existing methods, with asterisks indicating the top-performing method for each metric. **(C)** Visualization of annotated spatial regions identified by MMSpa (with 12 domains). **(D)** Visualization of spatial domains identified by SpaDo, SpaceFlow, conST, GraphST, SEDR, and STAGATE, with 23 domains. The underlying data for this figure can be found at https://doi.org/10.5281/zenodo.17451775.
(TIF)

S14 Fig Comparative analysis of MMSpa with other methods on the second applied E9.5 embryo Stereo-seq dataset (Slice #E9.5_E2_S3). **(A)** Visualization of spatial domains identified by nine existing methods with 13 domains. **(B)** Performance comparison of MMSpa against nine other existing methods, with asterisks indicating the top-performing method for each metric. **(C)** Visualization of spatial domains identified by SpaDo, SpaceFlow, conST, GraphST, SEDR, and STAGATE, with 16 domains. **(D)** Visualization of annotated spatial regions identified by MMSpa (with 16 domains). The underlying data for this figure can be found at https://doi.org/10.5281/zenodo.17451775.
(TIF)

S15 Fig Visualization and performance comparison of domain identification methods on the datasets from different platforms. **(A)** and **(B)** Application in the STARmap dataset. The visualization of domain identification, UMAP, PAGA trajectory graph, and Pseudo-Spatiotemporal Map (pSM) obtained by MMSpa and the nine compared methods. **(C)** and **(D)** Application in the osmFISH dataset. The visualization of domain identification, UMAP, PAGA trajectory graph, and pSM obtained by MMSpa and the nine compared methods. **(E)** Application in the MERFISH dataset. The visualization of domain identification obtained by SpaDo, SpaceFlow, conST, GraphST, SEDR, and STAGATE. The underlying data for this figure can be found at https://doi.org/10.5281/zenodo.17451775.
(TIF)

S16 Fig Ablation study of MMSpa by quantitative comparison. The accuracy metrics (ARI, NMI, and Purity) scores of MMSpa on 10× Visium datasets (the DLPFC dataset with 12 slices and human breast cancer dataset), the Stereo-Seq dataset (E9.5 mouse embryo dataset from slice #E9.5_E2_S3), the STARmap mouse cortex dataset, the osmFISH mouse somatosensory cortex dataset, and the MERFISH mouse hypothalamus dataset, by excluding edge removal strategy and masking strategies. The y-axis represents different datasets’ ARI, NMI, and Purity scores. The underlying data for this figure can be found at https://doi.org/10.5281/zenodo.17451775.
(TIF)

S17 Fig Ablation study of MMSpa on human DLPFC dataset (Slices #151674, #151672, and #151508) by downstream analysis. **(A)** The downstream analysis performances of MMSpa on slices 151674 (left), 151672 (median), and 151508 (right) are shown after removing the edge removal strategy. **(B)** The downstream analysis performances of MMSpa on slices 151674 (left), 151672 (median), and 151508 (right) are shown after removing the masked feature reconstruction strategy. The underlying data for this figure can be found at https://doi.org/10.5281/zenodo.17451775.
(TIF)

S18 Fig Parameter sensitivity analyses and the effect of dropout and SCE loss. **(A)** Effect of masking and re-masking ratio. Line charts show the ARI values of the three datasets as the masking and re-masking ratios change from 0.1 to 0.9. **(B)** Effect of lambda value. Line charts show the ARI values of the three datasets as lambda values change from 1 to 6. **(C)** and **(D)** Effect of k_cutoff and exp_cutoff values. Line charts show the ARI values of the two datasets as k_cutoff values change from 6 to 12, and exp_cutoff values change from 300 to 450. **(E)** Performance of MMSpa with different dropout rates in the mouse somatosensory cortex osmFISH dataset. **(F)** Performance of MMSpa using SCE and MSE loss on the #E9.5_E2_S3 mouse embryo dataset, the mouse sagittal anterior dataset, and the mouse visual cortex STARmap dataset. The underlying data for this figure can be found at https://doi.org/10.5281/zenodo.17451775.
(TIF)

S19 Fig Downstream analyses on DLPFC sections from biological replicates. Manually annotated layer structures, UMAP visualization, and PAGA graph of ST section #151507 from Donor 3, #151674 from Donor 1, #151669 from Donor 2, #151670 from Donor 2, #151671 from Donor 2, and #151672 from Donor 2. All the sections are from the human postmortem DLPFC tissue, and #151669, #151670, #151671, and #151672 sections are biological replicates from Donor 2. The underlying data for this figure can be found at https://doi.org/10.5281/zenodo.17451775.
(TIF)

S20 Fig Comparison of domain identification accuracy when all methods used the mclust algorithm in the DLPFC dataset. **(A)** Visualization of manual annotations for slice 151674. **(B)** Visualization of domain identification results for slice 151674 obtained by MMSpa. **(C)** Visualization of domain identification results for slice 151674 obtained by conST, SpaceFlow, stCMGAE, and SpaMask. The underlying data for this figure can be found at https://doi.org/10.5281/zenodo.17451775.
(TIF)

S21 Fig A simple investigation shows that spots with the greatest gene expression differences are less likely to belong to the same domain. **(A)** The box plot illustrates the distribution of the proportions of neighboring spots within the same domain as the central spot for each group category. **(B)** The bar chart illustrates the average proportion of neighboring spots within the same domain as the central spot for each group category. The underlying data for this figure can be found at https://doi.org/10.5281/zenodo.17451775.
(TIF)

S1 Note Detailed descriptions of the ablation experience. (DOCX)

S2 Note Details in the parameters’ sensitivity analyses and selection. (DOCX)

S3 Note Robustness tests. (DOCX)

S4 Note Comparison experiment using the same clustering algorithm across different methods. (DOCX)

S5 Note Edge removal strategy for spatial graph construction. (DOCX)

S6 Note Comparison of MMSpa’s performance with recently developed methods (stCMGAE, SpaMask, MAEST, m2ST, and SpaDo). (DOCX)

S7 Note Comparison of the existing spatial domain identification methods. (DOCX)

S1 Table Description of all ST datasets used in this study. (XLSX)

S2 Table Ablation study of MMSpa on the 12 slices of the DLPFC dataset. (XLSX)

S3 Table The ARI scores in DLPFC, MERFISH, osmFISH, and STARmap datasets when methods employed mclust. (XLSX)

S4 Table Methodological comparison of masked graph autoencoder methods for spatial transcriptomics. (XLSX)

S5 Table The ARI, NMI, and Purity scores of different methods for multi-slice domain identification on DLPFC samples. (XLSX)

S6 Table Parameters used by MMSpa. (XLSX)

S7 Table Running time of MMSpa in different ST datasets with different numbers of spots based on the A800 GPU with 40GB VRAM. (XLSX)

## Abbreviations

ARI: adjusted rand index
CA: Ammon’s horn area
CSF: cerebrospinal fluid
DB: Davies–Bouldin
DCIS/LCIS: ductal carcinoma in situ or lobular carcinoma in situ
DEGs: differential expression genes
DG: dentate gyrus area
DLPFC: dorsolateral prefrontal cortex
ECM: extracellular matrix
GAT: graph attention
GNNs: graph neural networks
GO: BP: Gene Ontology: Biological Process
HIPs: hippocampal areas
IDC: invasive ductal carcinoma
MSE: mean square error
NMI: normalized mutual information
PDAC: pancreatic ductal adenocarcinoma
pSM: Pseudo-Spatiotemporal Map
RHP: retrohippocampal area
RSP: retrosplenial area
SC: Silhouette Coefficient
ST: spatial transcriptome
UMAP: Uniform Manifold Approximation and Projection
VS: ventricular system
WM: white matter
