# Supplementary figures and images for: MMSpa is a deep learning-based tool that enhances the identification of spatial domains in spatial transcriptomics studies
Source: PLoS Biol. 2026 Jan 5;24(1):e3003580. doi: 10.1371/journal.pbio.3003580 (PMC12768284; doi:10.1371/journal.pbio.3003580)

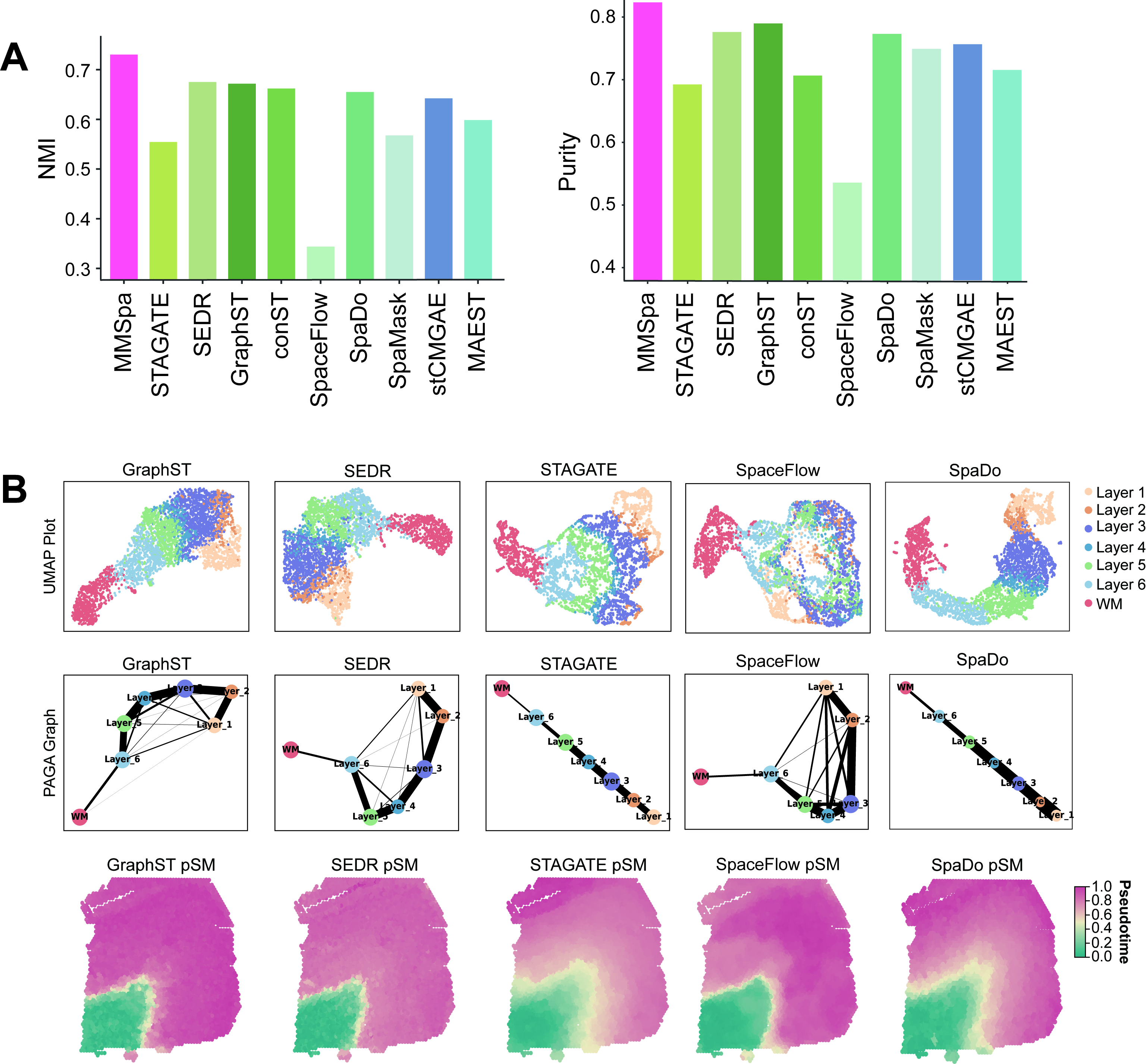

Supplement: S1 Fig — (A) Bar plots show the quantitative performance of MMSpa and nine other methods in domain identification accuracy across all 12 DLPFC tissue slices. The y-axis of each bar plot represents the NMI and Purity scores, respectively. (B) shows UMAP visualization, PAGA trajectory graph, and Pseudo-Spatiotemporal Map (pSM) generated by GraphST, SEDR, STAGATE, SpaceFlow, and SpaDo. The underlying data for this figure can be found at https://doi.org/10.5281/zenodo.17451775. (TIF) [file pbio.3003580.s001.tif]

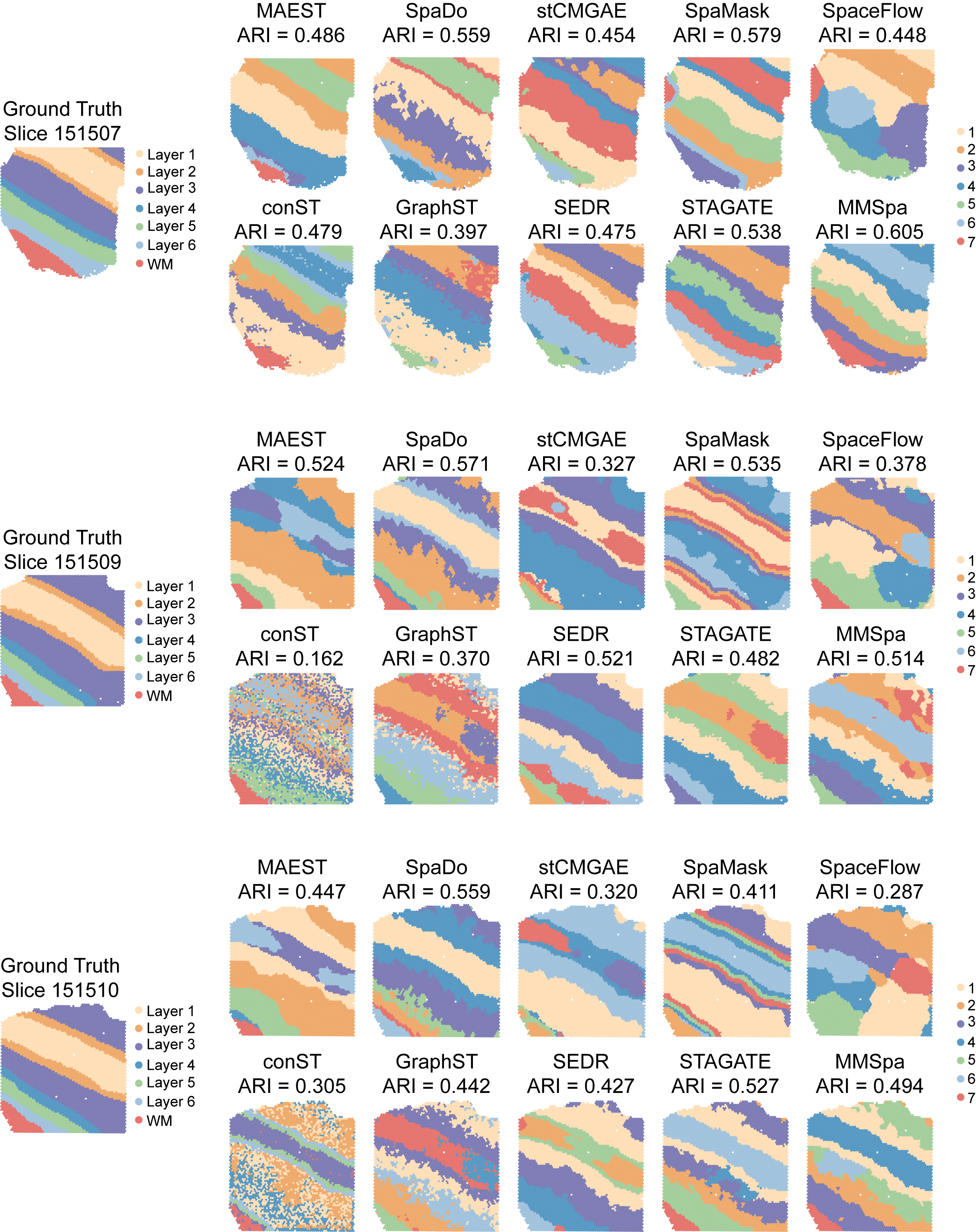

Supplement: S2 Fig — The underlying data for this figure can be found at https://doi.org/10.5281/zenodo.17451775. (TIF) [file pbio.3003580.s002.tif]

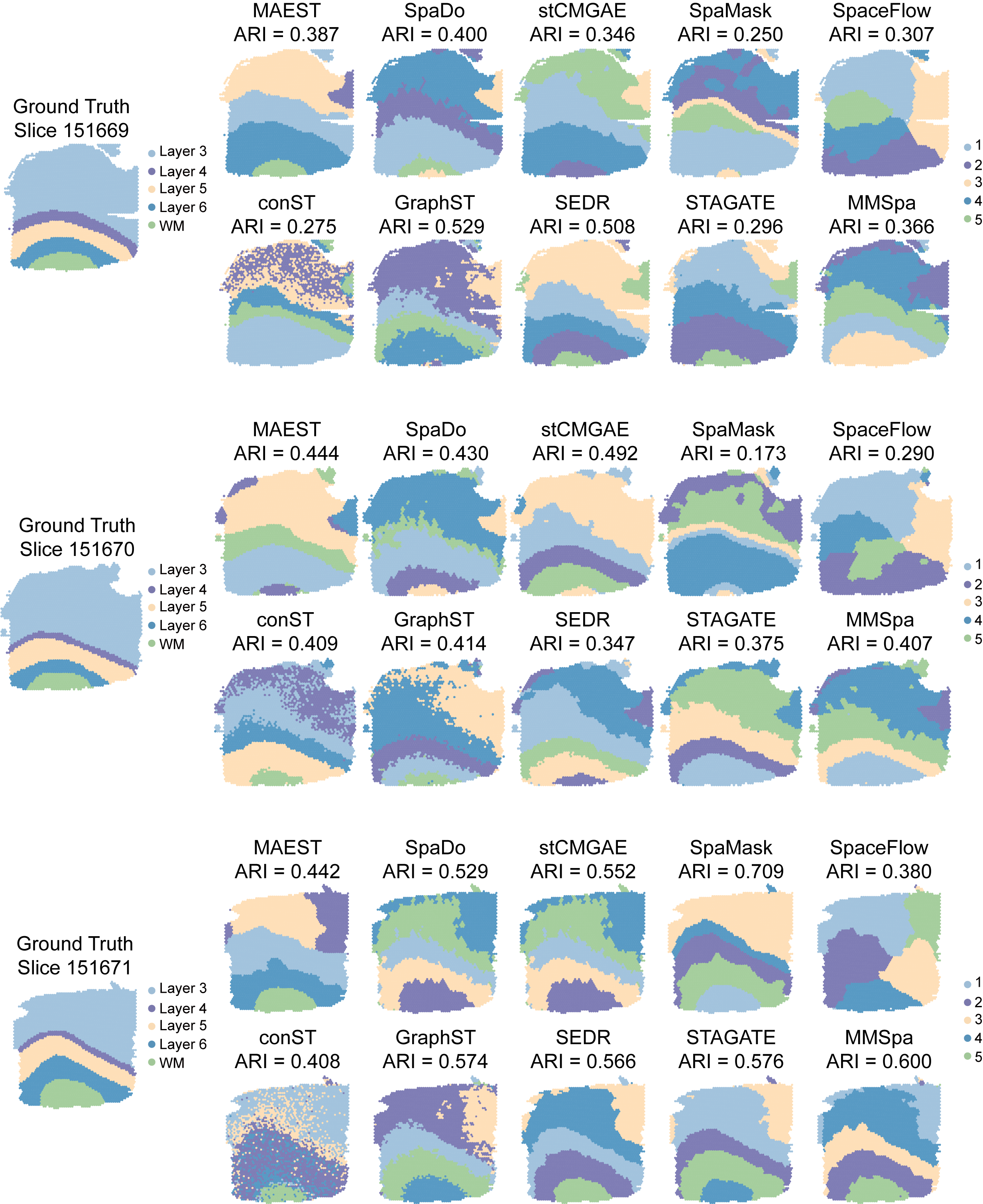

Supplement: S3 Fig — The underlying data for this figure can be found at https://doi.org/10.5281/zenodo.17451775. (TIF) [file pbio.3003580.s003.tif]

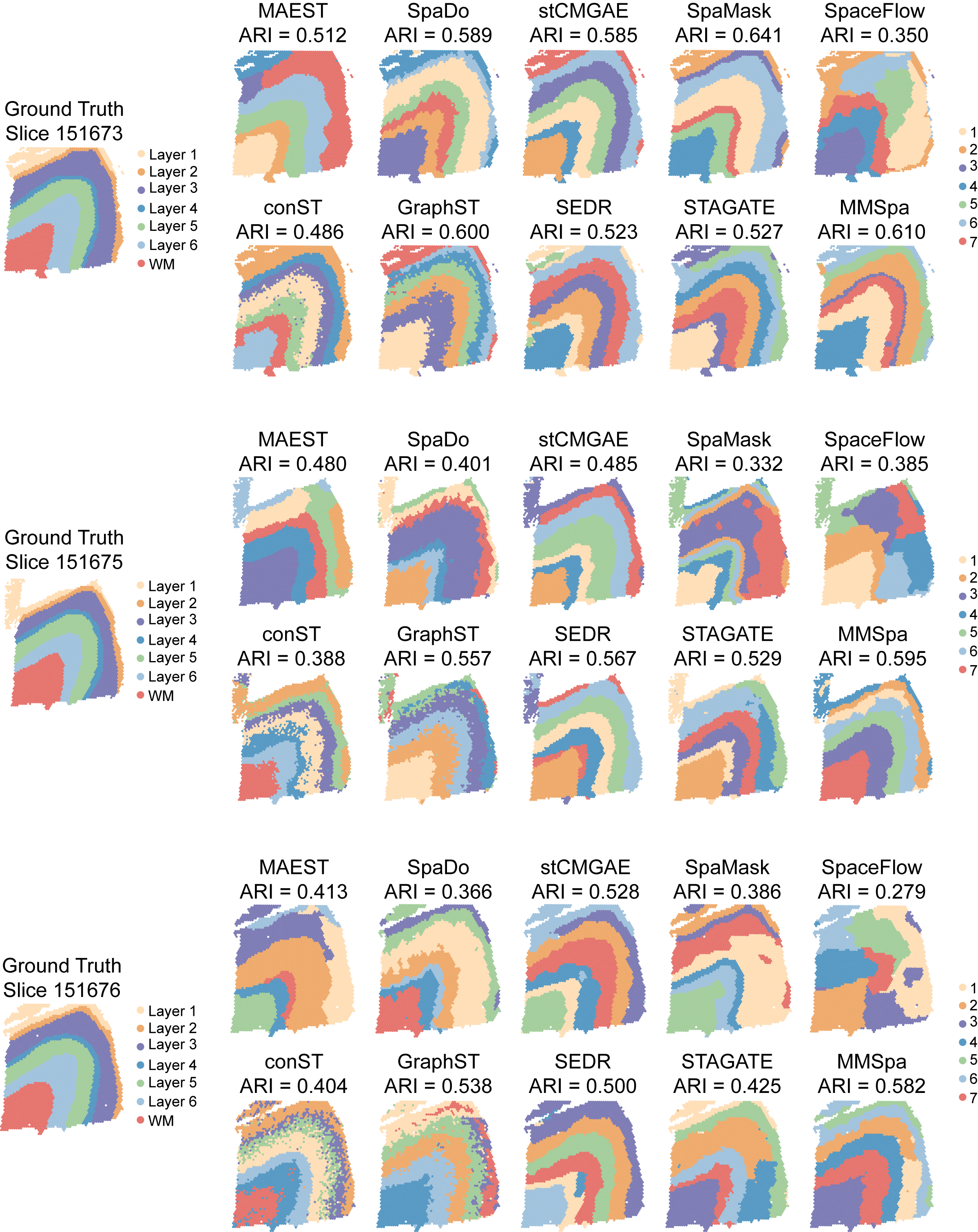

Supplement: S4 Fig — The underlying data for this figure can be found at https://doi.org/10.5281/zenodo.17451775. (TIF) [file pbio.3003580.s004.tif]

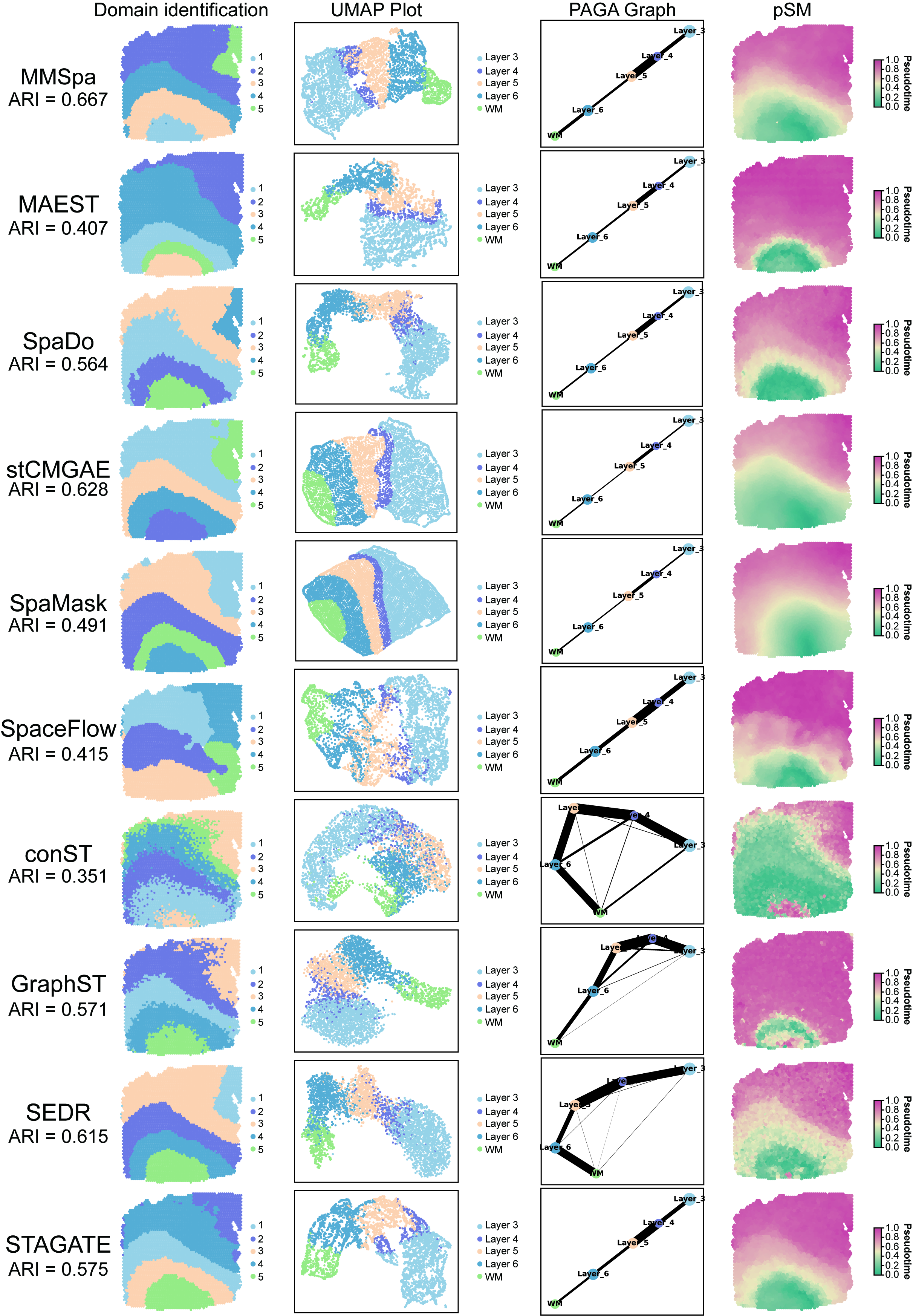

Supplement: S5 Fig — The underlying data for this figure can be found at https://doi.org/10.5281/zenodo.17451775. (TIF) [file pbio.3003580.s005.tif]

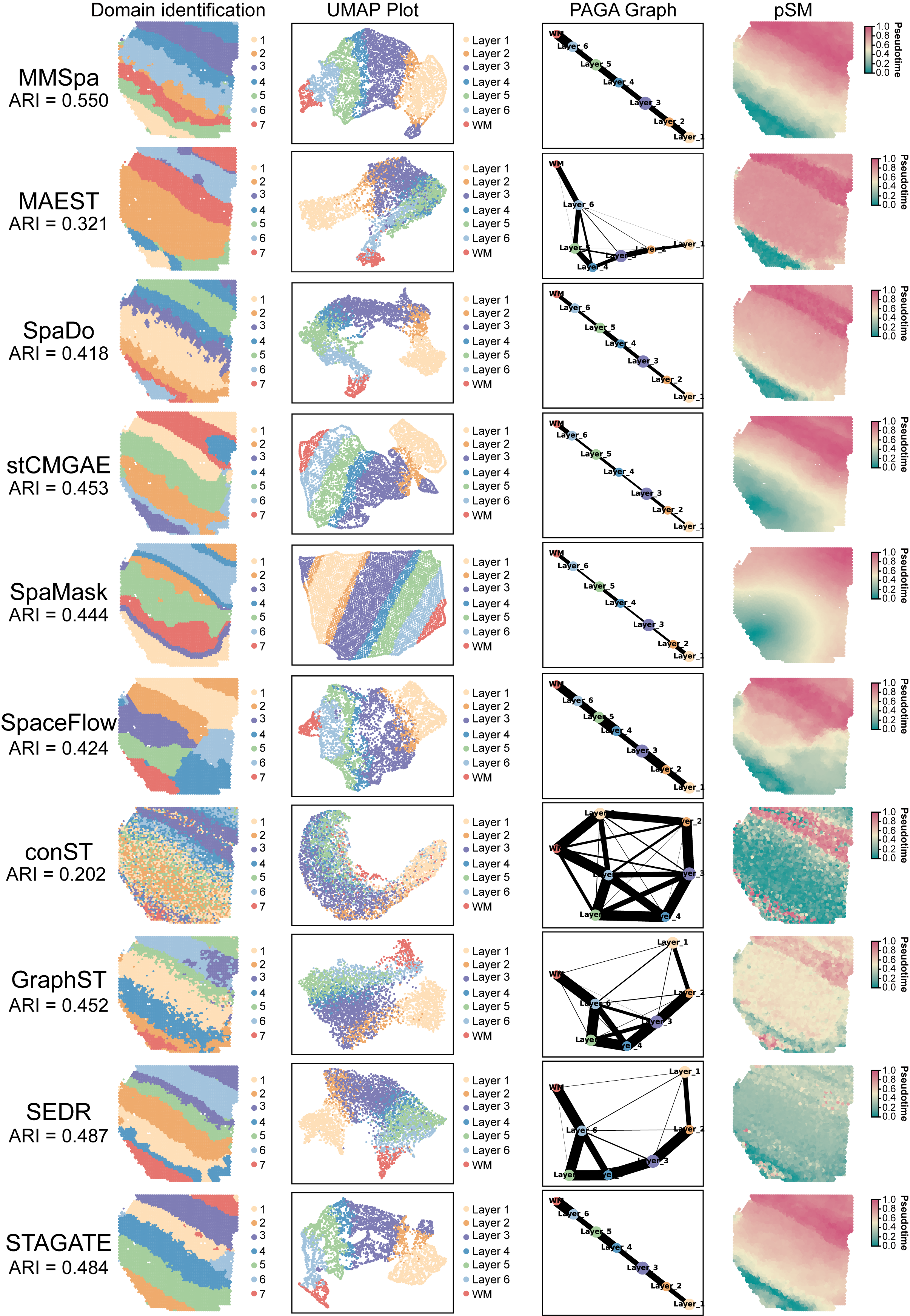

Supplement: S6 Fig — The underlying data for this figure can be found at https://doi.org/10.5281/zenodo.17451775. (TIF) [file pbio.3003580.s006.tif]

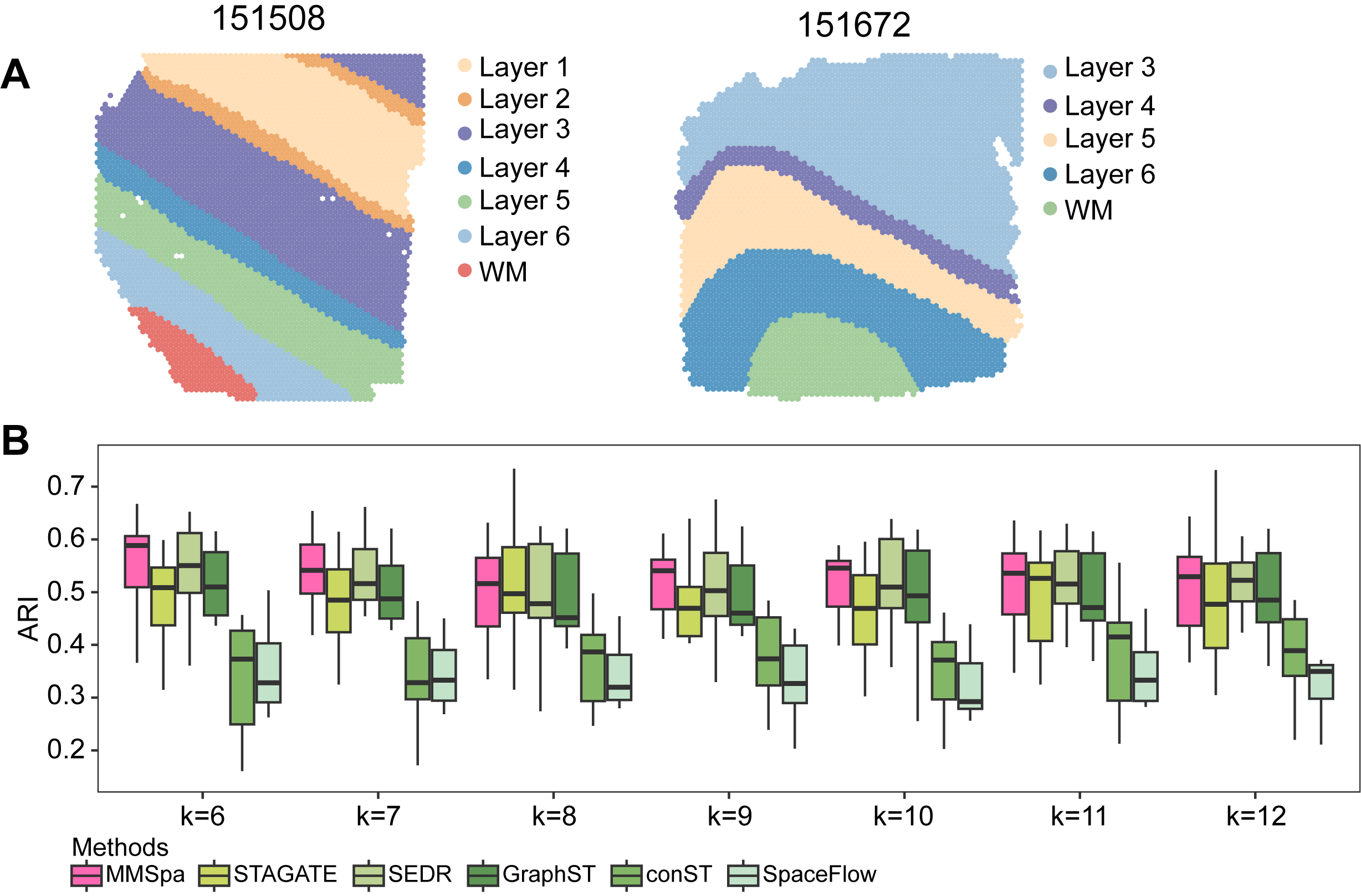

Supplement: S7 Fig — (A) Visualization of manual annotations for slices 151508 and 151672. (B) ARI boxplot for 6 methods on DLPFC datasets with different K (number of nearest neighbors). The underlying data for this figure can be found at https://doi.org/10.5281/zenodo.17451775. (TIF) [file pbio.3003580.s007.tif]

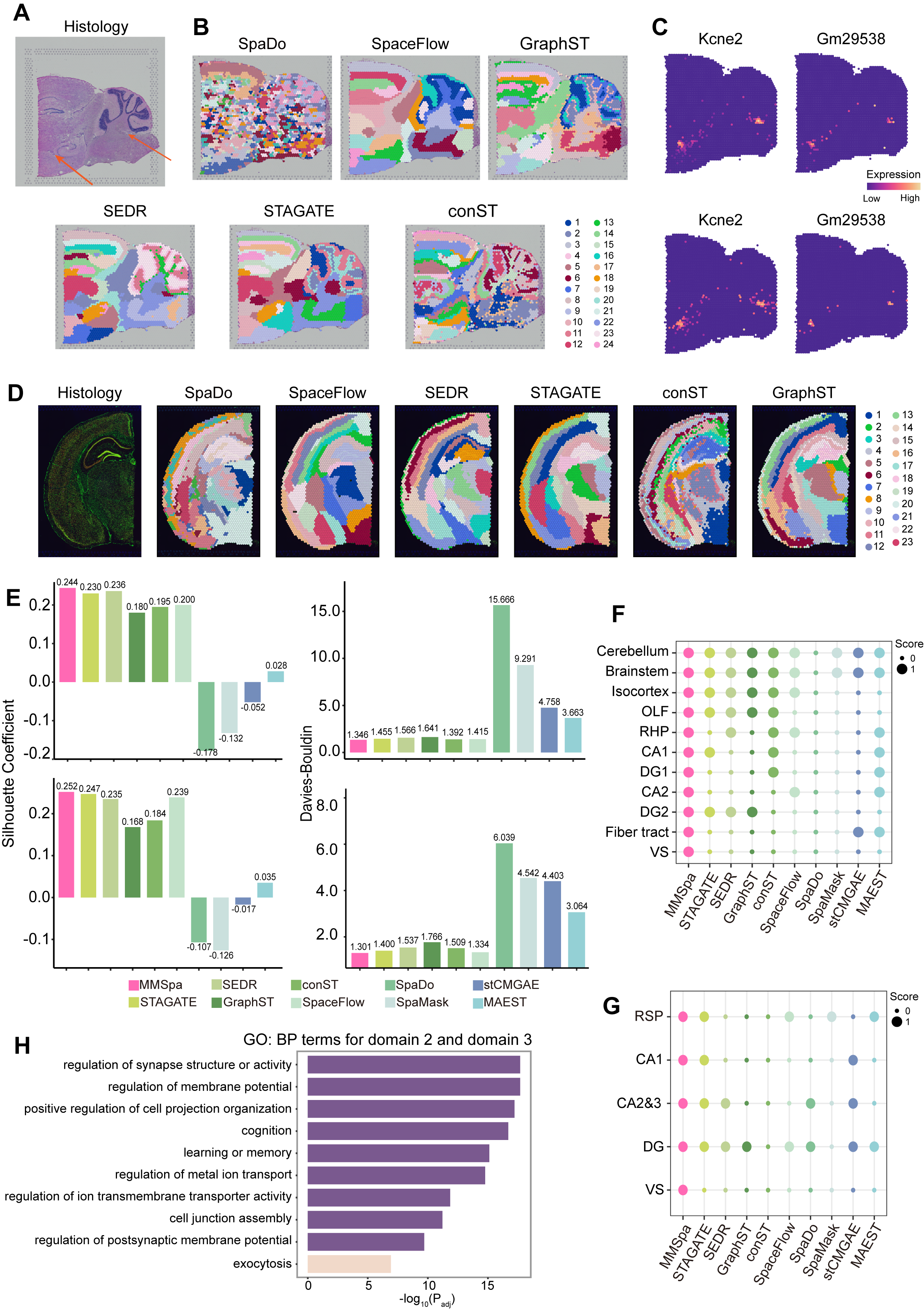

Supplement: S8 Fig — (A) Histological image of the mouse brain sagittal posterior ST dataset. (B) Visualization of domain identification results for the sagittal posterior using SpaDo, SpaceFlow, GraphST, SEDR, STAGATE, and conST. (C) Top marker genes of the identified VS region in MMSpa (domain 16) on the mouse brain sagittal posterior ST dataset (above) and another sagittal posterior replicate (below). (D) Histological image of the mouse brain coronal ST dataset, and visualization of domain identification results for the coronal using SpaDo, SpaceFlow, GraphST, SEDR, STAGATE, and conST. (E) Bar plots of the top-left panel, top-right panel, bottom-left panel, and bottom-right panel show the Silhouette Coefficient for the sagittal posterior, the Davies–Bouldin index for the sagittal posterior, the Silhouette Coefficient for the coronal section, and the Davies–Bouldin index for the coronal section, respectively. (F) Comparison of MMSpa and other methods in distinguishing different regions of the sagittal posterior. The x-axis represents different methods, while the y-axis corresponds to regions of the sagittal posterior as detailed in Fig 3B. Points on the plot indicate whether each method identified the corresponding region, with size reflecting the score (1 for detection, 0 for non-detection). (G) Comparison of MMSpa and other methods in distinguishing different regions of the coronal. The x-axis represents different methods, while the y-axis corresponds to regions of the coronal as detailed in Fig 3E. Points on the plot indicate whether each method identified the corresponding region, with size reflecting the score (1 for detection, 0 for non-detection). (H) The GO: BP terms for domains 2 and 3 of MMSpa on the mouse brain coronal ST dataset. The underlying data for this figure can be found at https://doi.org/10.5281/zenodo.17451775. (TIF) [file pbio.3003580.s008.tif]

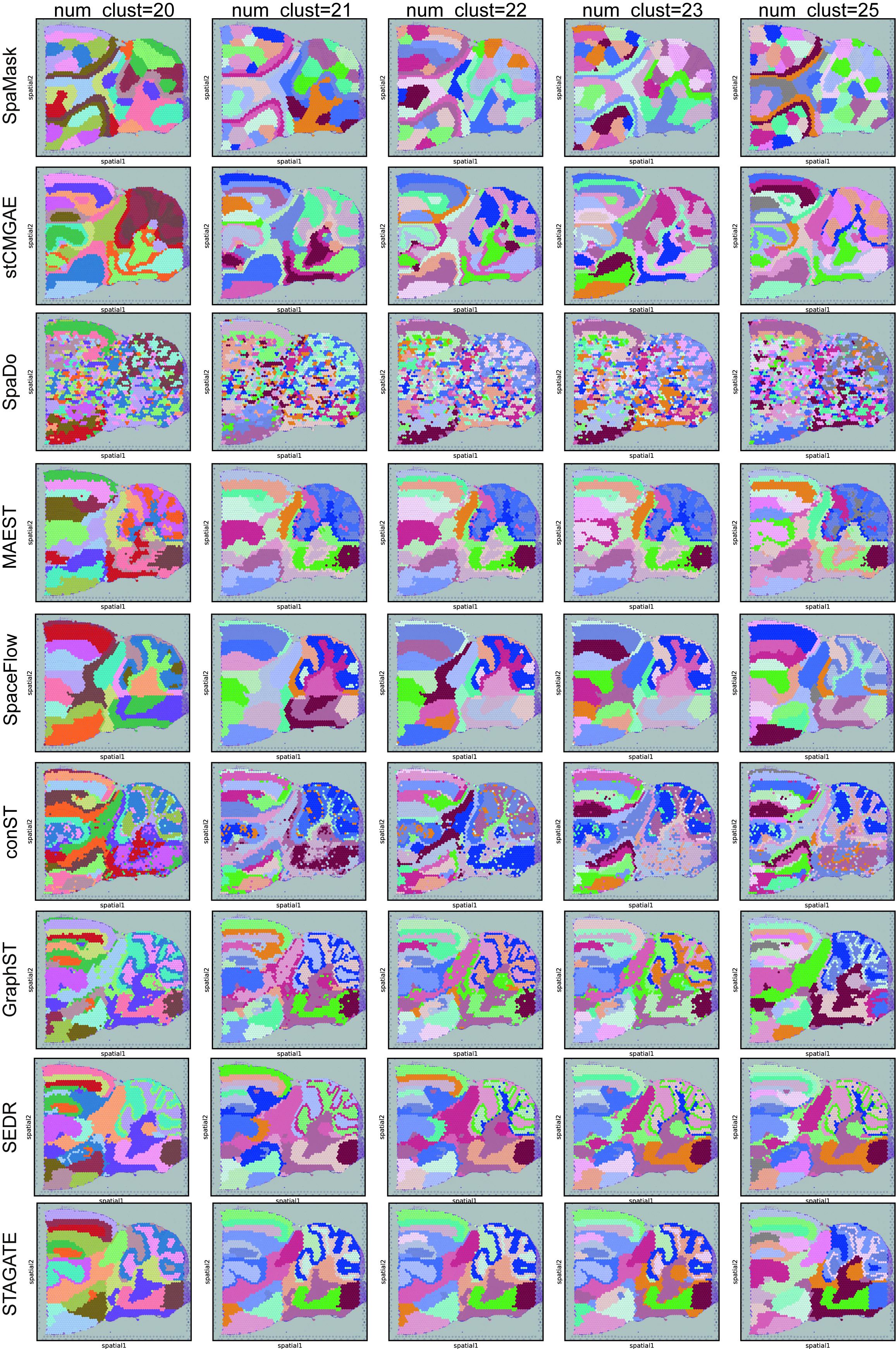

Supplement: S9 Fig — The underlying data for this figure can be found at https://doi.org/10.5281/zenodo.17451775. (TIF) [file pbio.3003580.s009.tif]

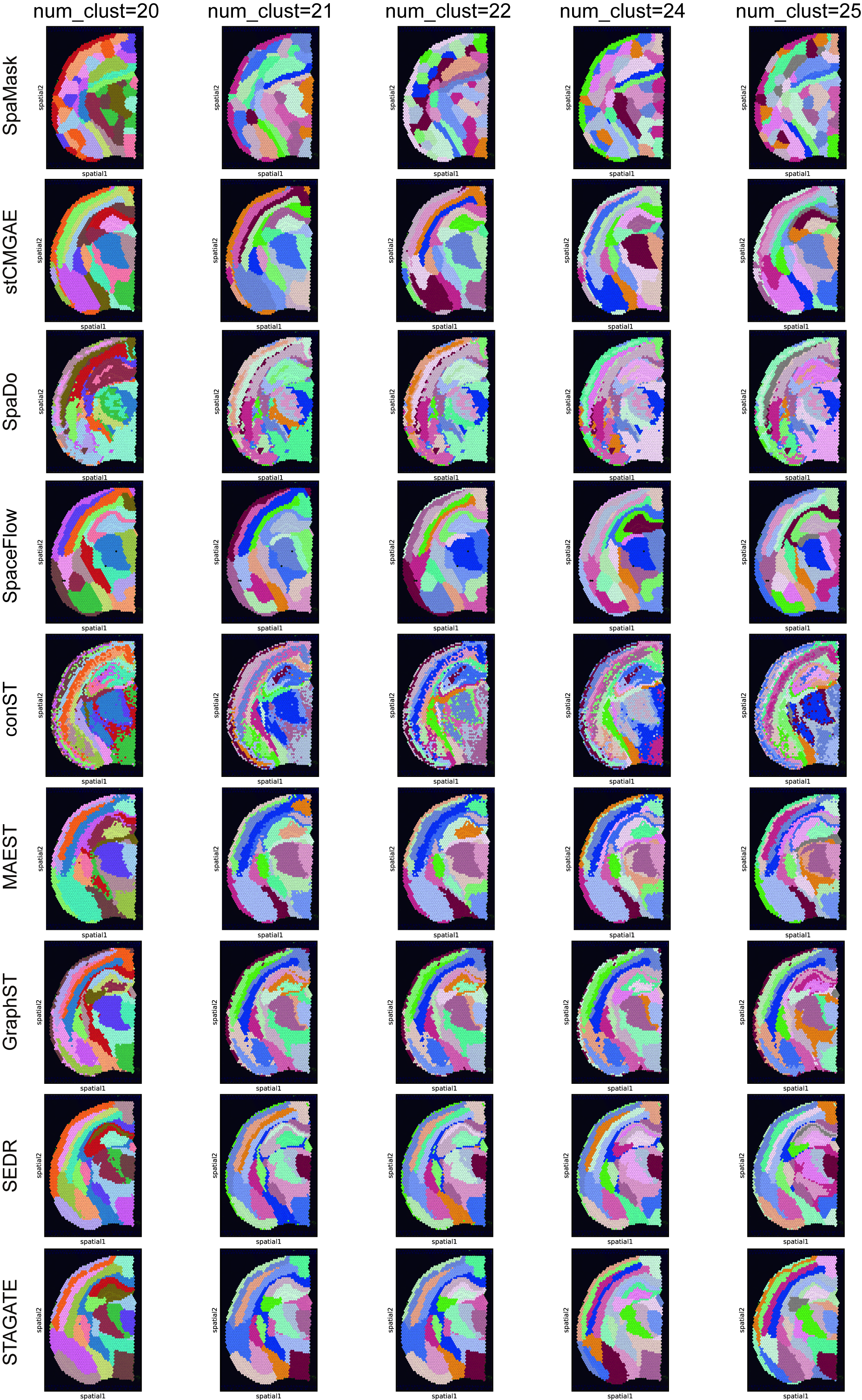

Supplement: S10 Fig — The underlying data for this figure can be found at https://doi.org/10.5281/zenodo.17451775. (TIF) [file pbio.3003580.s010.tif]

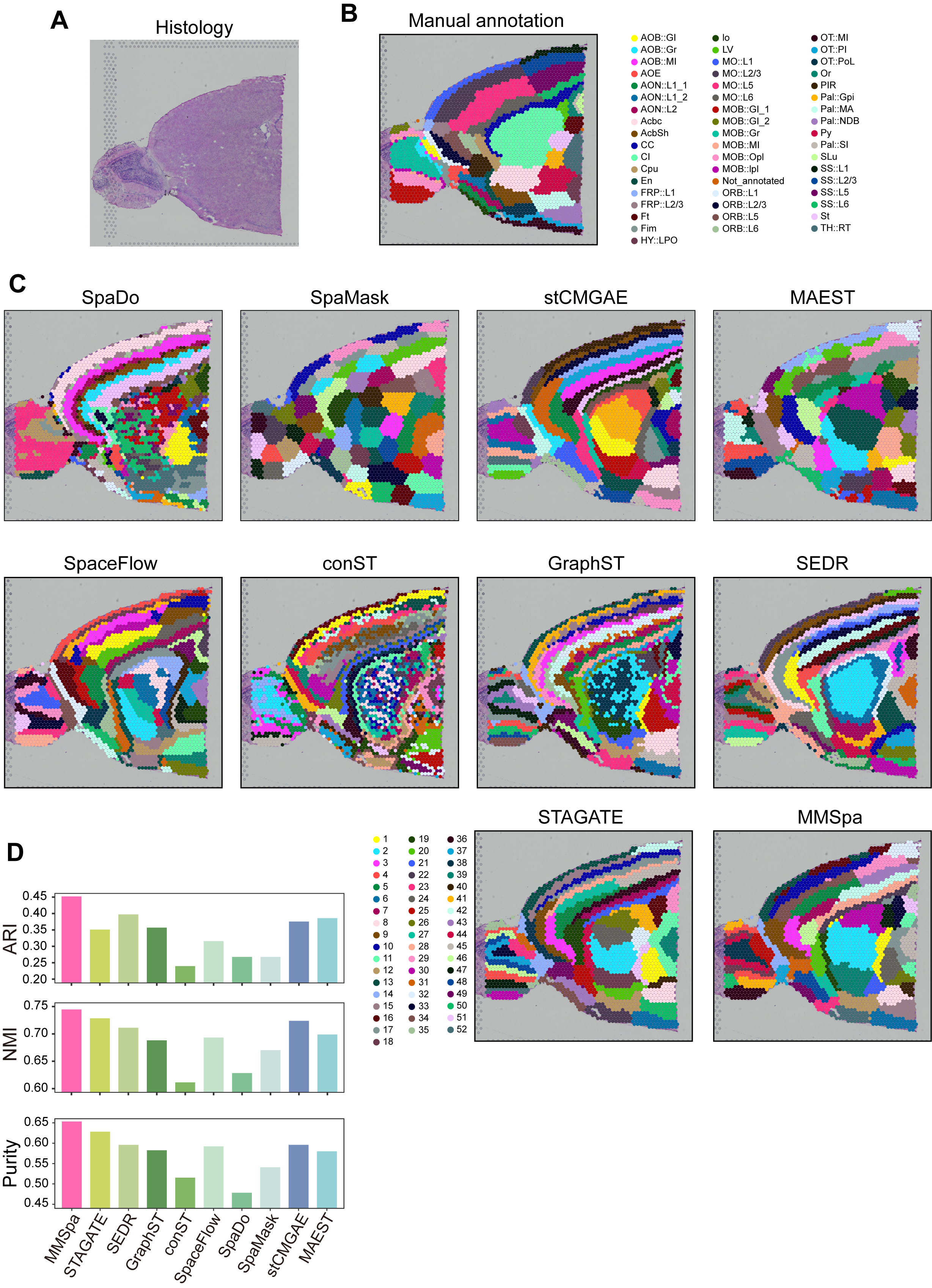

Supplement: S11 Fig — (A) Histological image of the anterior mouse brain ST dataset. (B) Visualization of manual annotations for the anterior mouse brain. (C) Visualization of domain identification results for the anterior mouse brain obtained by MMSpa and nine other methods. (D) Bar plots show the quantitative performance of MMSpa and nine other methods in domain identification accuracy. The y-axis of each bar plot represents the ARI, NMI, and Purity metrics, respectively. The underlying data for this figure can be found at https://doi.org/10.5281/zenodo.17451775. (TIF) [file pbio.3003580.s011.tif]

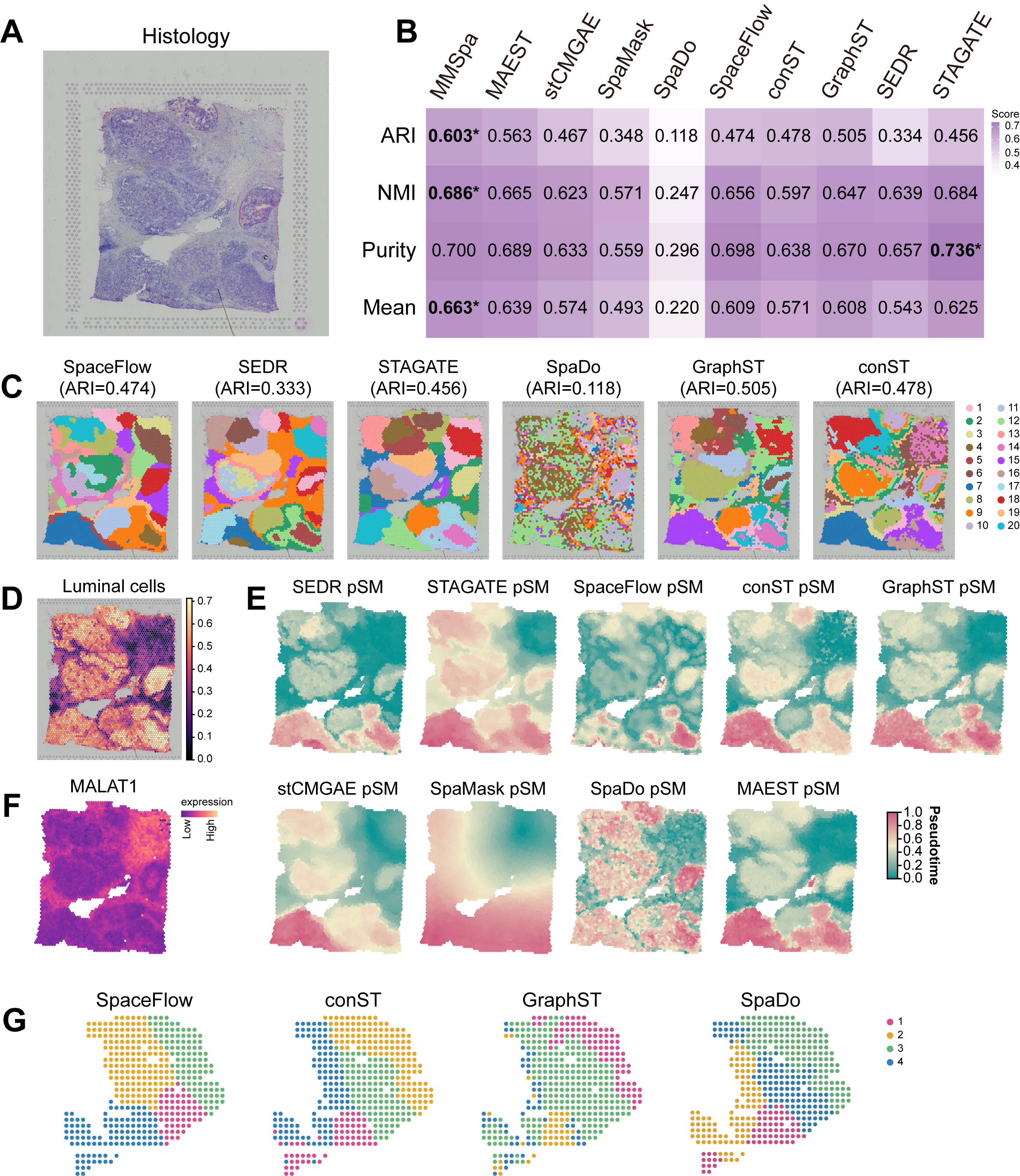

Supplement: S12 Fig — (A) Histological image of the human breast cancer slice. (B) Performance comparison of MMSpa against nine other existing methods, with asterisks indicating the top-performing method for each metric. (C) Visualization of domain identification results for the human breast cancer dataset using existing methods. (D) Spatial distribution of the luminal cells mapped by the CARD algorithm. (E) Pseudo-Spatiotemporal Maps (pSMs) generated by the nine compared methods. (F) Expression of the MALAT1 gene across the human breast cancer slice data. (G) The visualization results for SpaceFlow, conST, SpaDo, and GraphST. The underlying data for this figure can be found at https://doi.org/10.5281/zenodo.17451775. (TIF) [file pbio.3003580.s012.tif]

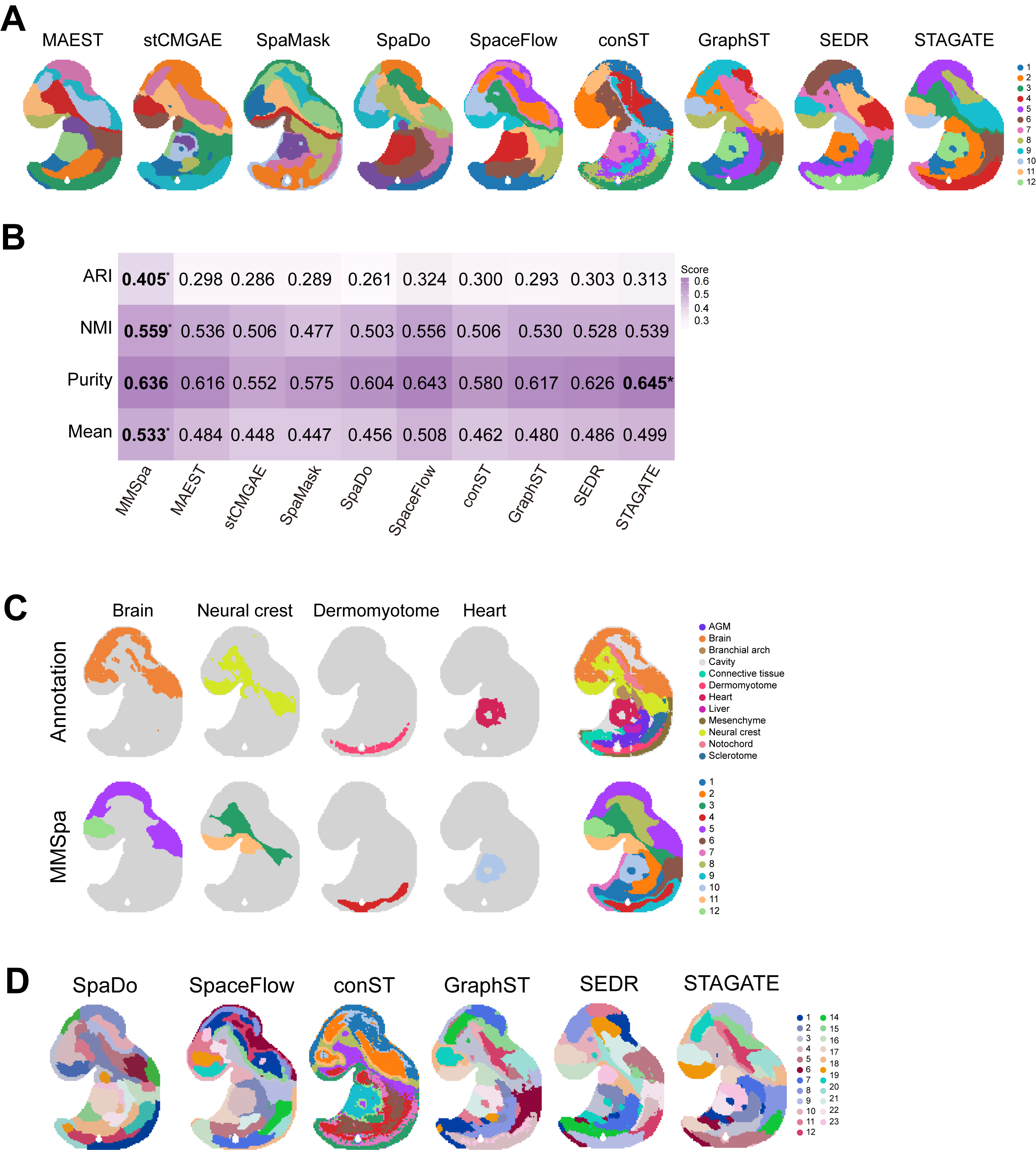

Supplement: S13 Fig — (A) Visualization of spatial domains identified by nine existing methods with 12 domains. (B) Performance comparison of MMSpa against nine other existing methods, with asterisks indicating the top-performing method for each metric. (C) Visualization of annotated spatial regions identified by MMSpa (with 12 domains). (D) Visualization of spatial domains identified by SpaDo, SpaceFlow, conST, GraphST, SEDR, and STAGATE, with 23 domains. The underlying data for this figure can be found at https://doi.org/10.5281/zenodo.17451775. (TIF) [file pbio.3003580.s013.tif]

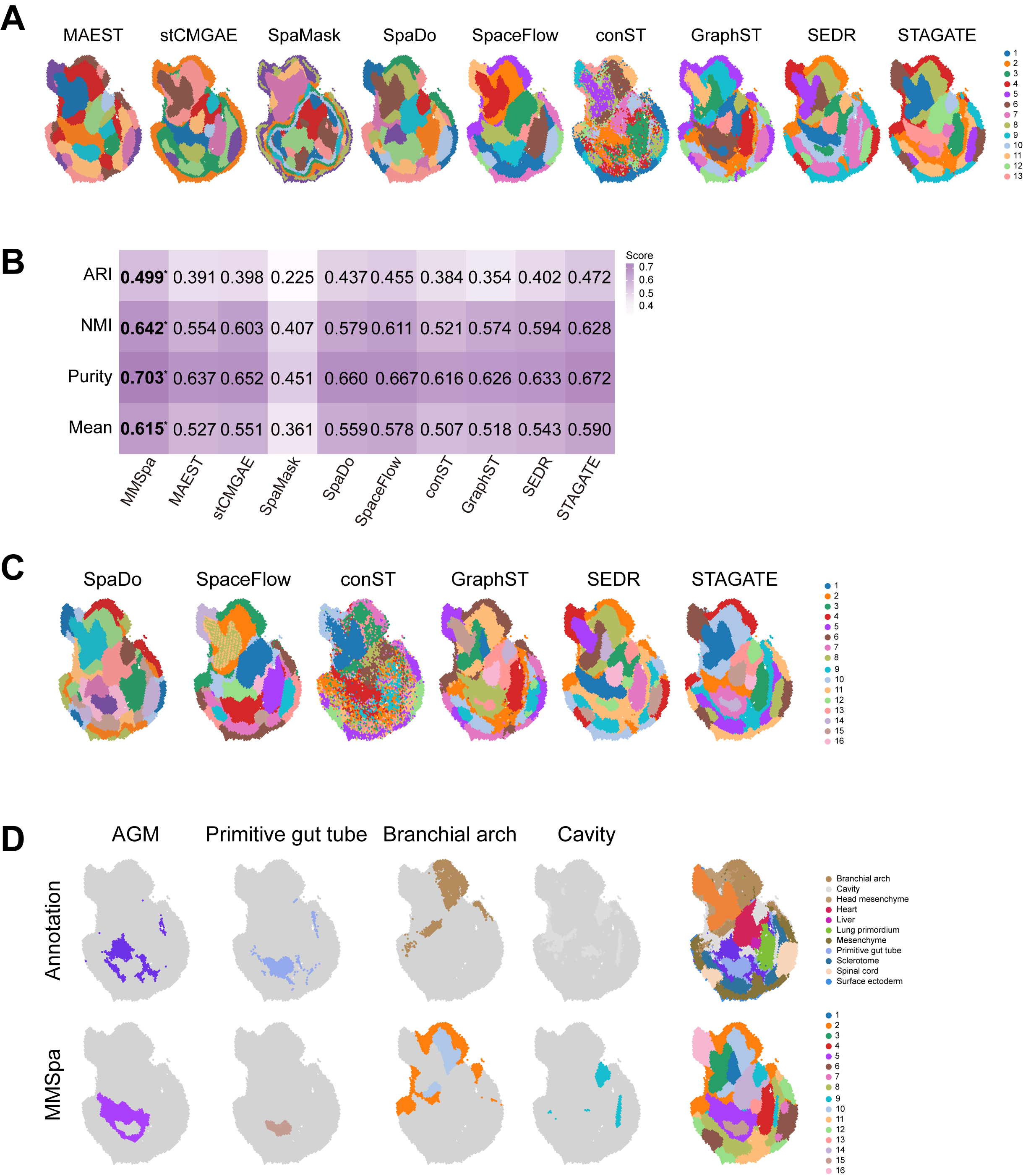

Supplement: S14 Fig — (A) Visualization of spatial domains identified by nine existing methods with 13 domains. (B) Performance comparison of MMSpa against nine other existing methods, with asterisks indicating the top-performing method for each metric. (C) Visualization of spatial domains identified by SpaDo, SpaceFlow, conST, GraphST, SEDR, and STAGATE, with 16 domains. (D) Visualization of annotated spatial regions identified by MMSpa (with 16 domains). The underlying data for this figure can be found at https://doi.org/10.5281/zenodo.17451775. (TIF) [file pbio.3003580.s014.tif]

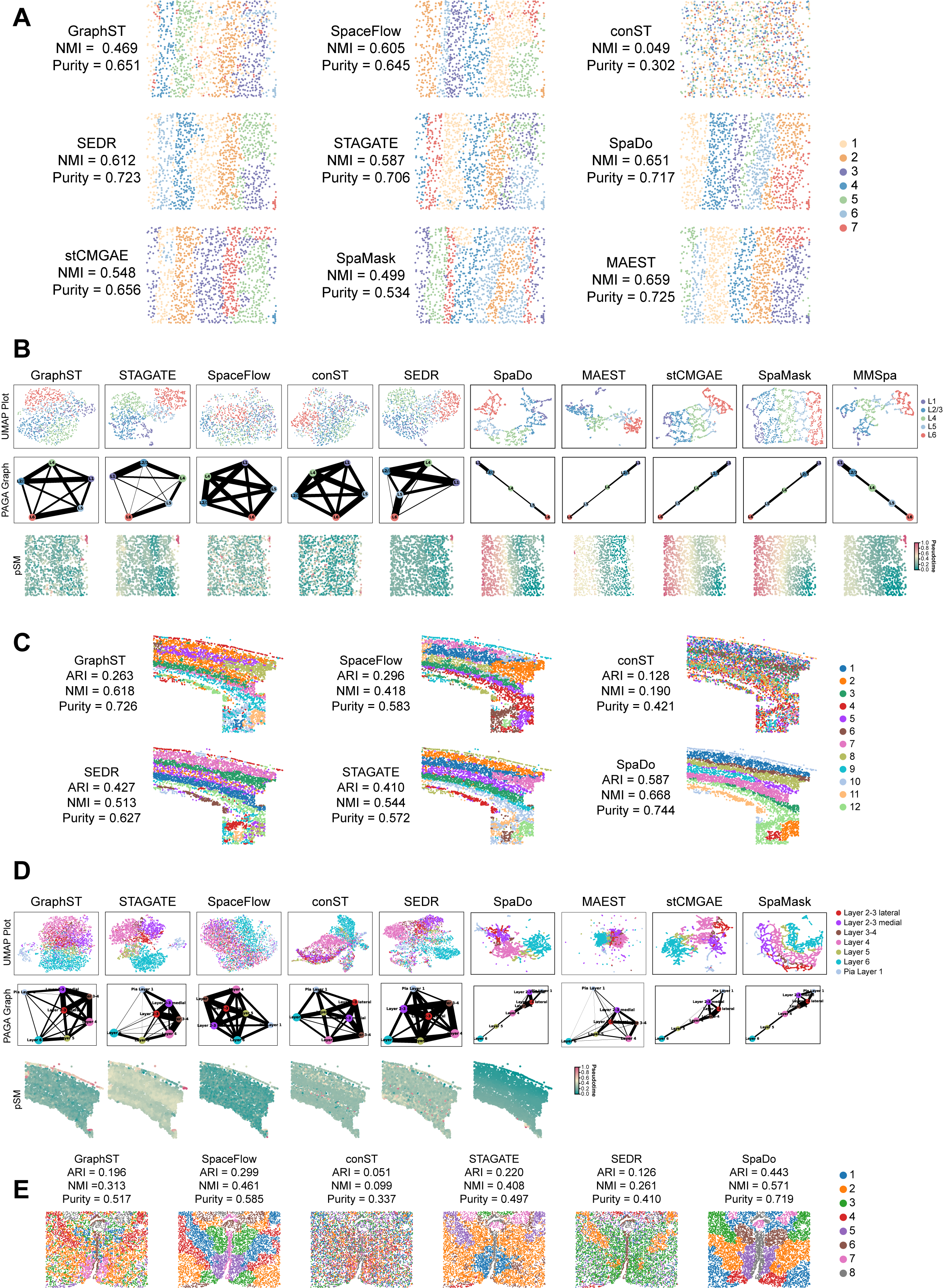

Supplement: S15 Fig — (A) and (B) Application in the STARmap dataset. The visualization of domain identification, UMAP, PAGA trajectory graph, and Pseudo-Spatiotemporal Map (pSM) obtained by MMSpa and the nine compared methods. (C) and (D) Application in the osmFISH dataset. The visualization of domain identification, UMAP, PAGA trajectory graph, and pSM obtained by MMSpa and the nine compared methods. (E) Application in the MERFISH dataset. The visualization of domain identification obtained by SpaDo, SpaceFlow, conST, GraphST, SEDR, and STAGATE. The underlying data for this figure can be found at https://doi.org/10.5281/zenodo.17451775. (TIF) [file pbio.3003580.s015.tif]

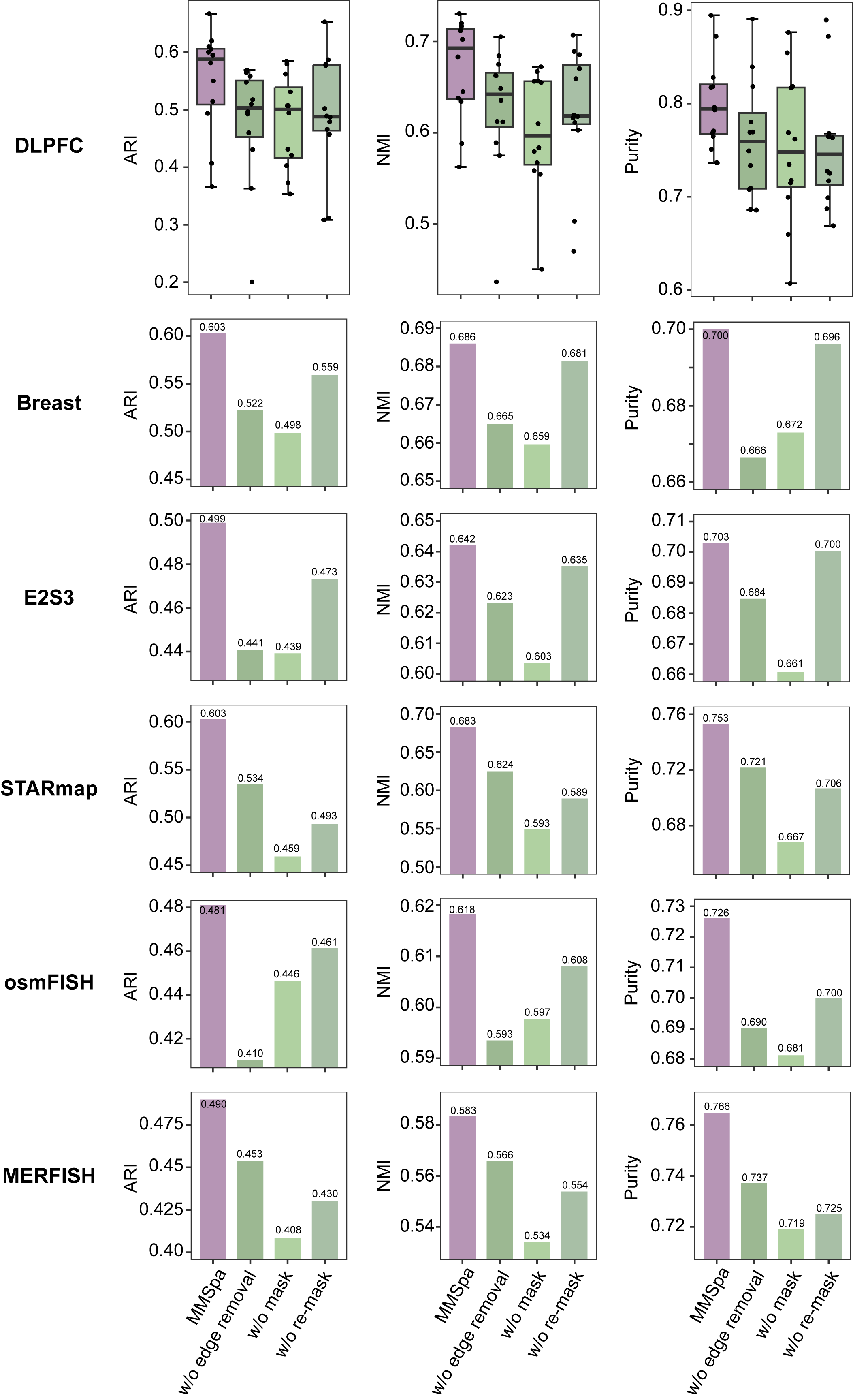

Supplement: S16 Fig — The accuracy metrics (ARI, NMI, and Purity) scores of MMSpa on 10× Visium datasets (the DLPFC dataset with 12 slices and human breast cancer dataset), the Stereo-Seq dataset (E9.5 mouse embryo dataset from slice #E9.5_E2_S3), the STARmap mouse cortex dataset, the osmFISH mouse somatosensory cortex dataset, and the MERFISH mouse hypothalamus dataset, by excluding edge removal strategy and masking strategies. The y-axis represents different datasets’ ARI, NMI, and Purity scores. The underlying data for this figure can be found at https://doi.org/10.5281/zenodo.17451775. (TIF) [file pbio.3003580.s016.tif]

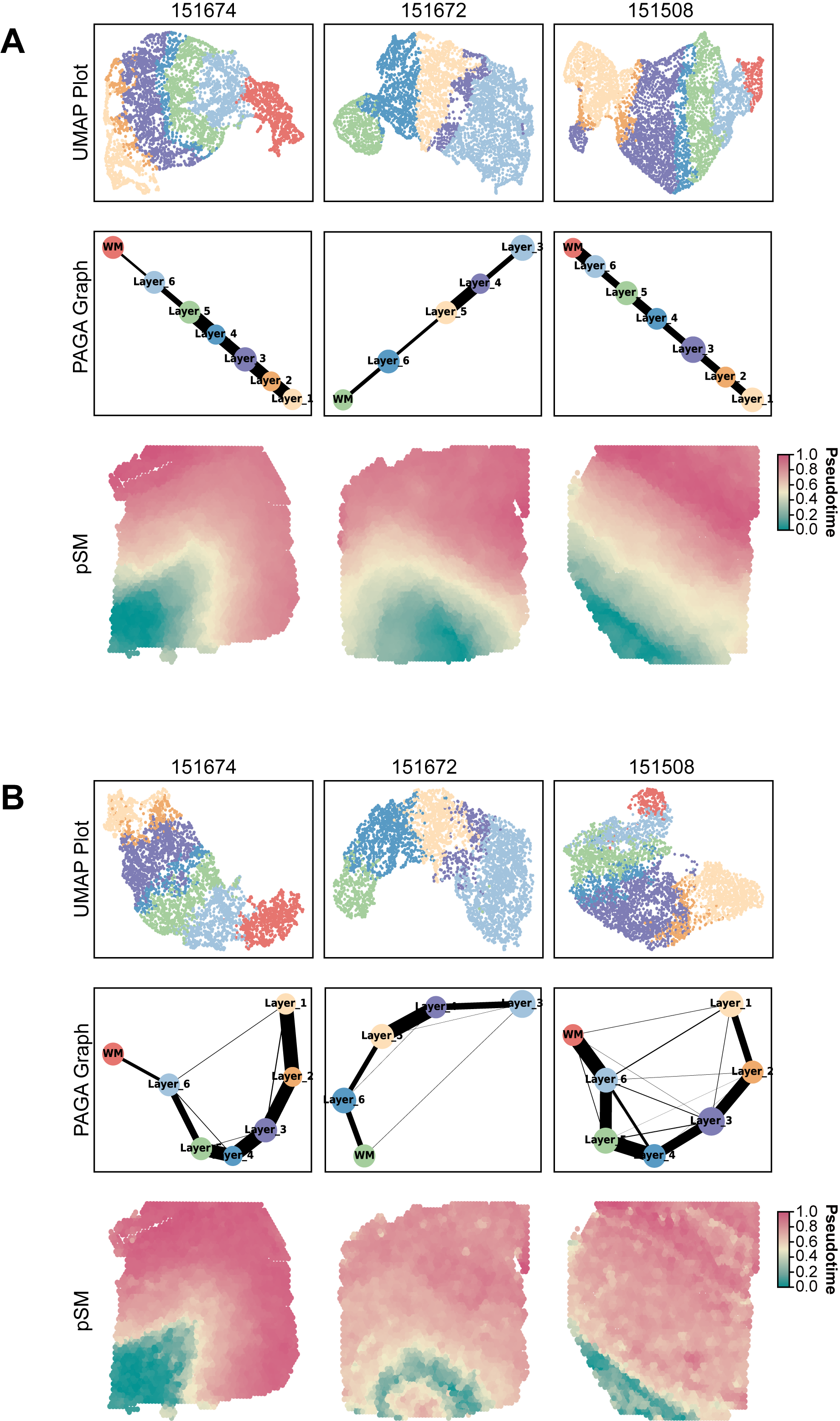

Supplement: S17 Fig — (A) The downstream analysis performances of MMSpa on slices 151674 (left), 151672 (median), and 151508 (right) are shown after removing the edge removal strategy. (B) The downstream analysis performances of MMSpa on slices 151674 (left), 151672 (median), and 151508 (right) are shown after removing the masked feature reconstruction strategy. The underlying data for this figure can be found at https://doi.org/10.5281/zenodo.17451775. (TIF) [file pbio.3003580.s017.tif]

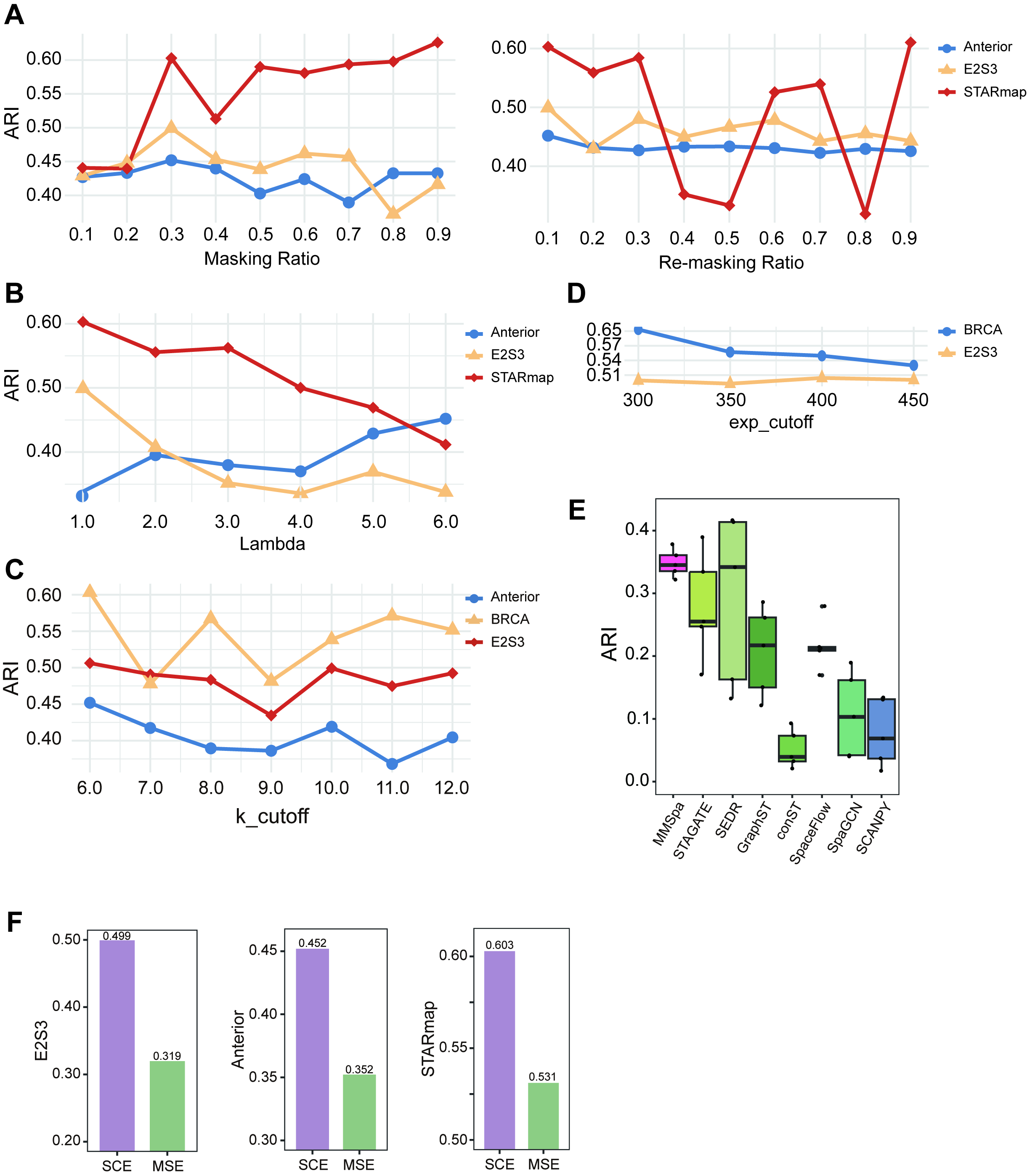

Supplement: S18 Fig — (A) Effect of masking and re-masking ratio. Line charts show the ARI values of the three datasets as the masking and re-masking ratios change from 0.1 to 0.9. (B) Effect of lambda value. Line charts show the ARI values of the three datasets as lambda values change from 1 to 6. (C) and (D) Effect of k_cutoff and exp_cutoff values. Line charts show the ARI values of the two datasets as k_cutoff values change from 6 to 12, and exp_cutoff values change from 300 to 450. (E) Performance of MMSpa with different dropout rates in the mouse somatosensory cortex osmFISH dataset. (F) Performance of MMSpa using SCE and MSE loss on the #E9.5_E2_S3 mouse embryo dataset, the mouse sagittal anterior dataset, and the mouse visual cortex STARmap dataset. The underlying data for this figure can be found at https://doi.org/10.5281/zenodo.17451775. (TIF) [file pbio.3003580.s018.tif]

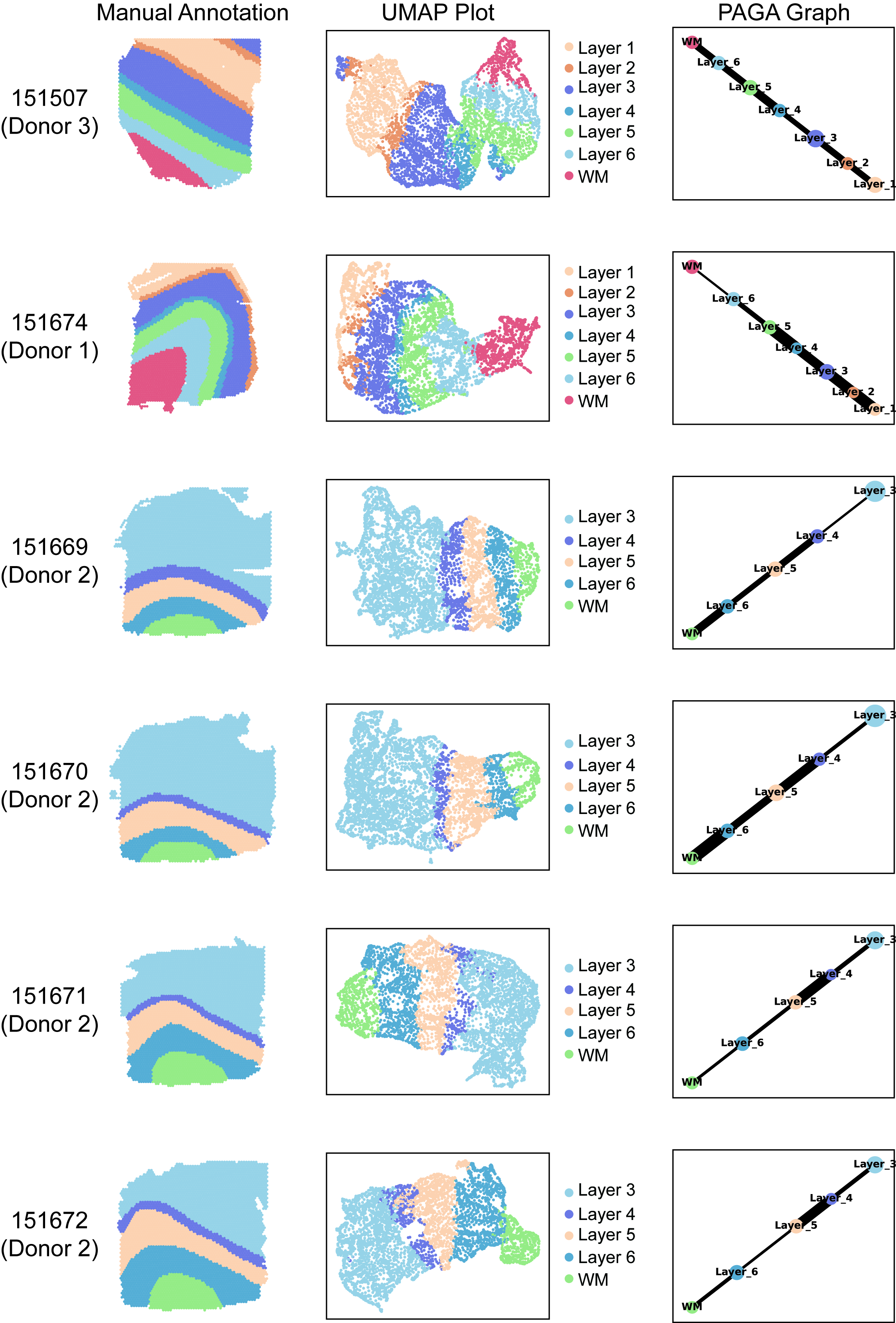

Supplement: S19 Fig — Manually annotated layer structures, UMAP visualization, and PAGA graph of ST section #151507 from Donor 3, #151674 from Donor 1, #151669 from Donor 2, #151670 from Donor 2, #151671 from Donor 2, and #151672 from Donor 2. All the sections are from the human postmortem DLPFC tissue, and #151669, #151670, #151671, and #151672 sections are biological replicates from Donor 2. The underlying data for this figure can be found at https://doi.org/10.5281/zenodo.17451775. (TIF) [file pbio.3003580.s019.tif]

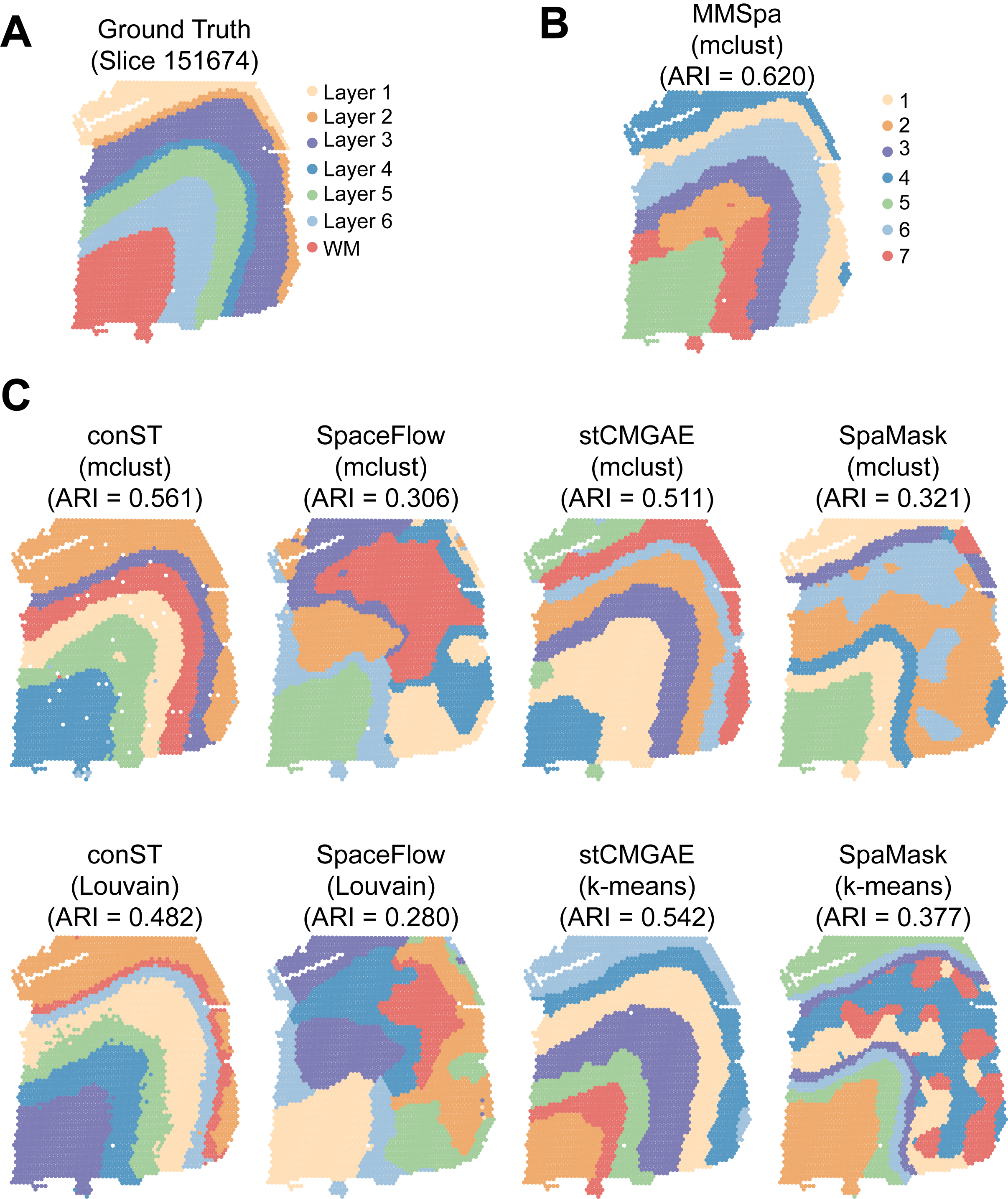

Supplement: S20 Fig — (A) Visualization of manual annotations for slice 151674. (B) Visualization of domain identification results for slice 151674 obtained by MMSpa. (C) Visualization of domain identification results for slice 151674 obtained by conST, SpaceFlow, stCMGAE, and SpaMask. The underlying data for this figure can be found at https://doi.org/10.5281/zenodo.17451775. (TIF) [file pbio.3003580.s020.tif]

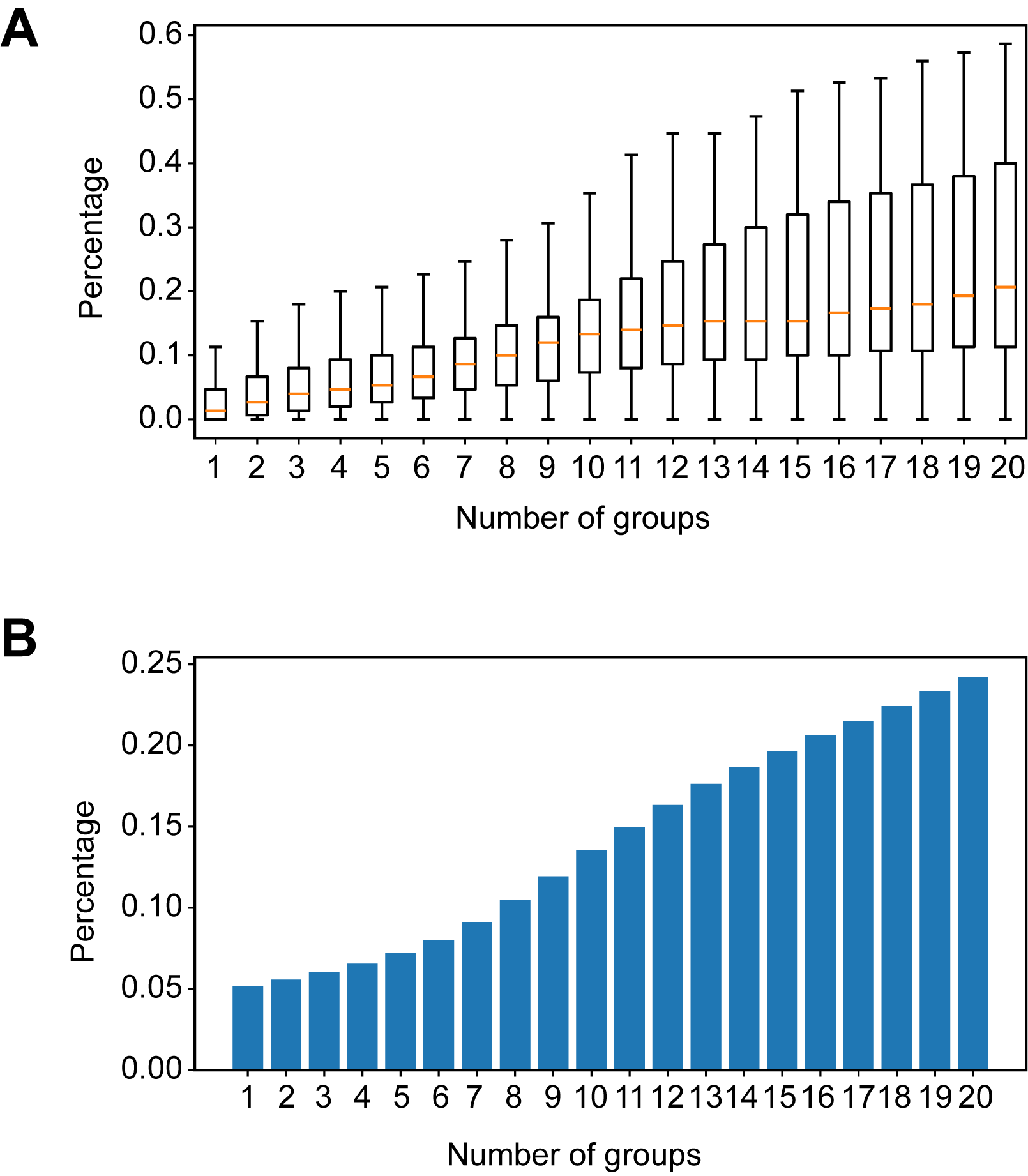

Supplement: S21 Fig — (A) The box plot illustrates the distribution of the proportions of neighboring spots within the same domain as the central spot for each group category. (B) The bar chart illustrates the average proportion of neighboring spots within the same domain as the central spot for each group category. The underlying data for this figure can be found at https://doi.org/10.5281/zenodo.17451775. (TIF) [file pbio.3003580.s021.tif]
