# Supplementary material for: MMSpa is a deep learning-based tool that enhances the identification of spatial domains in spatial transcriptomics studies
Source: PLoS Biol. 2026 Jan 5;24(1):e3003580. doi: 10.1371/journal.pbio.3003580 (PMC12768284; doi:10.1371/journal.pbio.3003580)
Supplement: S1 Note — (DOCX) [file pbio.3003580.s022.docx]

**Note S1. Detailed descriptions of the ablation experience**

*1. Ablation study of the edge removal strategy*

In this study, we construct the spatial graph by calculating only the Euclidean distance between spots based on their spatial coordinates, without constructing the opponent spatial graph. Specifically, by setting the parameter “exp_cutoff” to 0, the final spatial graph remains the initial graph, and no other model structures or parameters are altered.

*2. Ablation study of the masking strategy*

We directly input the gene expression data into the encoder, bypassing the random selection and masking of gene expression for the spots. Since the masking operation on the gene expression is removed, the reconstruction of all spots’ gene expression is incorporated into the loss function. We continue to use the SCE loss function, where the loss is calculated as the difference between the predicted and original gene expression for each spot. The total loss is then averaged across all spots. Aside from removing the masking operation before the encoder, no other changes are made to the model’s structure. In this case, the masking ratio is set to 0, and all other model parameters are preserved.

*3. Ablation study of the re-masking strategy*

Here, we feed the encoder’s output directly into the decoder without applying any masking to the encoder’s output features. In this case, the re-masking ratio is set to 0, and the model’s other structure and parameters are unchanged.
