# Supplementary material for: MMSpa is a deep learning-based tool that enhances the identification of spatial domains in spatial transcriptomics studies
Source: PLoS Biol. 2026 Jan 5;24(1):e3003580. doi: 10.1371/journal.pbio.3003580 (PMC12768284; doi:10.1371/journal.pbio.3003580)
Supplement: S2 Note — (DOCX) [file pbio.3003580.s023.docx]

**Note S2.** **Details in the parameters’ sensitivity analyses and selection**

We conducted sensitivity analysis for five parameters, where three parameters (masking ratio, re-masking ratio, and lambda for SCE loss) for model framework, and two parameters (the number of neighbor spots for initial spatial graph construction, and the number of neighbor spots for opponent spatial graph construction) for spatial graph construction.

First, we tested the sensitivity of MMSpa to the model framework parameters by using three ST datasets (the mouse sagittal forebrain dataset with 52 annotated regions, the #E9.5_E2_S3 mouse embryo dataset, and the mouse visual cortex STARmap dataset), where the masking and re-masking ratios ranged from 0.1 to 0.9 (increments of 0.1) and lambda values ranged from 1 to 6 (increments of 1). When testing one parameter, the other parameters were fixed. We calculated the ARI value for each dataset. The results were visualized using line charts to show the influence of different masking and re-masking ratios and lambda values (Fig S18A and 18B).

It can be seen that in most cases, when the masking ratio was set with a low value (e.g., 0.1 or 0.2), the domain identification accuracy was suboptimal. This could be related to the model not being challenging enough to capture useful features when the masking ratio is too low. Additionally, in most datasets, performance also decreased when the masking ratio exceeded 0.3. This might be related to the excessive information loss. However, it is worth noting that in the mouse visual cortex STARmap dataset, our proposed model still maintained good domain identification accuracy at higher masking ratios. This might be related to the clear biological hierarchy of the mouse cortex and the high resolution of the STARmap dataset. Neighboring spots are more likely to belong to the same biological layer when constructing the spatial graph. Even if most of the neighboring spots are masked, the spot can still recover its original features from the remaining few neighbors, allowing the model to perform well at higher masking ratios. Overall, we recommend setting the masking ratio to 0.3.

As for the re-masking ratio, the results for all three datasets show that the model performance is more stable when the re-masking ratio is below 0.3. The re-masking strategy can be interpreted as a form of “dropout” before the decoder or a regularization operation during the model training process, and it may cause information loss when the ratio is too large. It can be seen that the model performs best when the re-masking ratio is 0.1 in most cases. The optimal ratio varies across the dataset. In general, we recommend setting the re-masking ratio to 0.1 or below 0.3.

As for lambda, a scaling strategy to modulate the contribution of each spot based on reconstruction difficulty, it is used to balance the SCE loss to reconstruct different spots. We observed that as lambda increases, it brings benefits to the model performance on complex biological structures, such as in the case of the mouse sagittal forebrain dataset with 52 annotated regions. Furthermore, in general cases, lambda set to 1 is sufficient to ensure the reconstruction effect of SCE loss. If lambda is set too large, it may cause the model to miss the optimal solution during training. We recommend setting the lambda to 1 in general cases and lambda > 1 in complex biological cases.

Then, we tested the sensitivity of MMSpa to spatial graph construction with edge removal strategy taking the #E9.5_E2_S3 mouse embryo dataset (single-cell resolution) and the Human Breast cancer (BRCA) dataset (spot resolution) as examples, where the number of neighbor spots for initial spatial graph construction (k_cutoff) ranged from 6 to 12 (increments of 1), and the number of neighbor spots for opponent spatial graph construction (exp_cutoff) ranged from 300 to 450 (increments of 50) (Fig S18C and S18D).

The k_cutoff parameter is used to select the number of neighbors closest to the central spot coordinate. We found that for spot resolution ST data, MMSpa performs optimally when k_cutoff is set to 6, while for single-cell resolution ST data, MMSpa demonstrates greater robustness to variations in k_cutoff values (Fig S18C). This difference is likely due to the larger microsphere diameter in spot resolution sequencing. As the k_cutoff value increases, the spatial graph in spot resolution includes a broader range of physical space, which may extend beyond the spot’s domain boundary, causing slight fluctuations in model performance. In contrast, a single-cell resolution ST data with the smaller microsphere diameter ensures that even with a higher k_cutoff value, the impact on the domain boundary remains minimal, allowing for more stable model performance. We recommend setting the k_cutoff to 6 for ST datasets with spot resolution and to 6-12 that can be adjusted for other single-cell resolution ST datasets.

The exp_cutoff parameter is used to select the neighbor spot with the greatest gene expression distance from the central spot. We observed that MMSpa performs optimally when the exp_cutoff value is set to 300 at both resolutions. Furthermore, MMSpa demonstrates more robustness with single-cell resolution ST data (Fig S18D). This difference may be attributed to the characteristics of ST data at varying resolutions. At spot resolution, a spot contains multiple single cells, while at single-cell resolution, each spot contains only one single cell. As the exp_cutoff value increases, the actual number of single cells in the spot resolution data increases significantly more than in the single-cell resolution data, leading to a broader range of performance fluctuations in the model under spot resolution. We recommend setting the exp_cutoff value to 300 as a general guideline, with a range of 300-450 that can be adjusted based on specific data characteristics.

We also compared MMSpa’s performance using both SCE and MSE loss functions across three ST datasets: the #E9.5_E2_S3 mouse embryo dataset, the mouse sagittal anterior dataset, and the mouse visual cortex STARmap dataset (Fig S18F). The ARI values were calculated for each loss function on these datasets. The results indicate that the SCE loss significantly enhances MMSpa’s performance. Specifically, SCE loss improved domain identification accuracy (ARI) by 18%, 10%, and 7.2% compared to MSE loss for the three datasets, respectively.
