# Supplementary material for: MMSpa is a deep learning-based tool that enhances the identification of spatial domains in spatial transcriptomics studies
Source: PLoS Biol. 2026 Jan 5;24(1):e3003580. doi: 10.1371/journal.pbio.3003580 (PMC12768284; doi:10.1371/journal.pbio.3003580)
Supplement: S3 Note — (DOCX) [file pbio.3003580.s024.docx]

**Note S3. Robustness tests**

First, we conducted robustness tests on the mouse somatosensory cortex osmFISH dataset by performing dropout. We randomly set a fraction of non-zero expression values to zero, with dropout ratios ranging from 0.1 to 0.5 in increments of 0.1. We observed that MMSpa maintains high domain identification accuracy across the five dropout levels (Fig S18E). Compared to other methods, we found that MMSpa exhibits the smallest performance fluctuation when the dropout rate changes, with the smallest variance (0.000381) in domain identification accuracy, indicating its robustness. Although SEDR achieved higher accuracy in a few cases, its variance was significantly larger (0.014985) and may easier to be affected by the dropout rate.

Then, we conducted additional analyses to further validate the consistency and robustness of MMSpa. We utilized the human postmortem dorsolateral prefrontal cortex (DLPFC) dataset, which includes 12 spatial transcriptomics (ST) sections from the DLPFC tissue. These sections were obtained from three independent neurotypical adult donors, with four spatial replicates per donor (Donor 1: #151673, #151674, #151675, #151676; Donor 2: #151669, #151670, #151671, #151672; Donor 3: #151507, #151508, #151509, #151510) (Table S1). The original study has annotated four to six cortical layers and white matter (WM) regions, and each section showed a continuous hierarchical structure. We first conducted downstream analyses using three independent biological replicates that all from the DLPFC tissue. One section from each donor was selected (#151507 from Donor 3, #151669 from Donor 2, and #151674 from Donor 1) for UMAP visualization and pseudo-temporal trajectory inference (PAGA) (Fig S19). The results across all three replicates showed high consistency: UMAP projections revealed distinct hierarchical separation across cortical layers (L1–L6) and WM in all replicates. PAGA trajectory analysis also displayed continuous linear developmental trajectories connecting adjacent cortical layers in all replicates, aligning with the known spatial continuity of the DLPFC’s laminar architecture. Additionally, we conducted downstream analyses within a single donor’s replicates by analyzing four biological replicates that were all from Donor 2 (#151669, #151670, #151671, #151672) (Fig S19). We found that both UMAP and PAGA results remained highly consistent across these biological replicates from the same sample. These results demonstrate that MMSpa can robustly and effectively capture spatial domain characteristics and produce accurate, reproducible trajectory inferences across biological replicates. The consistency in hierarchical separation and pseudotemporal ordering further supports MMSpa’s reliability in modeling spatially continuous tissue architectures.
