# Supplementary material for: MMSpa is a deep learning-based tool that enhances the identification of spatial domains in spatial transcriptomics studies
Source: PLoS Biol. 2026 Jan 5;24(1):e3003580. doi: 10.1371/journal.pbio.3003580 (PMC12768284; doi:10.1371/journal.pbio.3003580)
Supplement: S4 Note — (DOCX) [file pbio.3003580.s025.docx]

**Note S4****. Comparison experiment using the same clustering algorithm across different methods**

We conducted a comparison experiment using the same clustering algorithm across different methods. Since MMSpa defaults to mclust clustering, we compared domain identification accuracy when all methods employed mclust. We excluded Seurat, BayesSpace, SCANPY, and SpaGCN from this comparison because they are end-to-end methods that integrate their own specific clustering approaches. Additionally, we excluded STAGATE, SEDR, and GraphST, as they also default to mclust clustering. Instead, we focused on comparing the domain identification accuracy of SpaceFlow, conST, stCMGAE, and SpaMask when their clustering methods were switched to mclust.

First, we performed mclust clustering on the embeddings generated by these four methods and compared their accuracy using the Adjusted Rand Index (ARI) on the DLPFC, MERFISH, osmFISH, and STARmap datasets (Table S3). The results showed that even when all methods used mclust clustering, MMSpa achieved the highest ARI scores. For MERFISH and osmFISH datasets, the mclust results for conST returned the error “NULLType” object is not subscriptable, which may indicate that the mclust clustering method is not suitable for its embeddings.

We further examined the DLPFC slice 151674 (Fig S20). When SpaceFlow and conST used Leiden clustering, their domain identification ARI scores were 0.280 and 0.482, respectively. SpaceFlow failed to correctly identify domain boundaries and shapes, while conST showed suboptimal performance in recognizing layer 3 thickness. After switching to mclust clustering, both methods showed improved accuracy, with SpaceFlow’s ARI increasing to 0.306 and conST’s ARI rising to 0.561. However, the slice domain visualization further showed that SpaceFlow still failed to correctly identify domain boundaries and the shape of the mouse cerebral cortex, and although conST maintained excellent domain boundary identification, with a slight improvement in layer 3 thickness identification, it was still suboptimal. When stCMGAE and SpaMask used k-means clustering, their domain identification ARI scores were 0.542 and 0.377, respectively. After switching to mclust clustering, both methods showed decreased accuracy, with stCMGAE’s ARI decreasing to 0.511 and SpaMask’s ARI decreasing to 0.321. In comparison, MMSpa achieved the best domain identification performance in separating continuous layers, providing clear boundaries with less noise, and identifying the annotated layers, with the highest accuracy (ARI=0.620).
