# Supplementary material for: MMSpa is a deep learning-based tool that enhances the identification of spatial domains in spatial transcriptomics studies
Source: PLoS Biol. 2026 Jan 5;24(1):e3003580. doi: 10.1371/journal.pbio.3003580 (PMC12768284; doi:10.1371/journal.pbio.3003580)
Supplement: S5 Note — (DOCX) [file pbio.3003580.s026.docx]

**Note S5. Edge removal strategy for spatial graph construction**

We aimed to construct a spatial graph that accurately and comprehensively captures the spatial relationships within domain information. Based on the assumption that spatially proximal spots exhibit greater similarity, and we initially constructed the spatial graph using Euclidean distance derived from spatial coordinates. This approach resulted in an adjacency matrix representing the relationships among spots in physical space (See “Methods”).

However, the initial spatial graph may lack granularity in characterizing spatial domains, particularly because spots at the boundaries of domains may not exactly belong to the same domain as their neighbors. Generally, spatial domains consist of clusters of spots that share similar gene expression patterns and spatial coherence. Consequently, spots within the same domain are more likely to exhibit similar gene expression profiles, whereas those in distinct domains may show substantial differences in gene expression. Although the degree of this similarity may not be precisely defined, we hypothesize that spots with more significant gene expression differences are more likely to not belong to the same domain.

To test this hypothesis, we analyzed the DLPFC dataset (Slide: 151508) with ground truth annotations. We first calculated the distance between spots based on their gene expression profiles (See “Methods”). For each spot, treated as a centroid, we identified the $K$ spots most distant from it, and we tested this across a total of $n$ groups (here $n = 1, ..., 20$). Specifically, for group category $n$, choose $K$ neighbors ranked between $((n-1)K+1)$-th and $(nK)$-th farthest from the central spot. Subsequently, we computed the ratio of these $K$ distant spots that reside in the same domain as their respective centroid, based on ground truth annotations (here $k=150$). Finally, calculate the average and median ratio of domain membership ratios across all central spots for each group category.

Our analysis revealed a notable increase in this ratio as $K$ increased (Fig S21). More importantly, in the case of selecting the $1-150$ most distant spots ($n=1$), the median of this ratio is nearly $0$, with a mean of $0.05$. When $n>2$, this ratio starts to increase significantly. These observations indicated that spots with greater gene expression differences are more likely to not belong to the same domain.

Based on this result, we constructed an opposed spatial graph by applying a spatial remoteness principle to remove the erroneous connections from the initial spatial graph (See “Overview” and “Methods”). This approach aims to enhance the characterization of spatial domain information at the input level.
