# Supplementary material for: MMSpa is a deep learning-based tool that enhances the identification of spatial domains in spatial transcriptomics studies
Source: PLoS Biol. 2026 Jan 5;24(1):e3003580. doi: 10.1371/journal.pbio.3003580 (PMC12768284; doi:10.1371/journal.pbio.3003580)
Supplement: S6 Note — (DOCX) [file pbio.3003580.s027.docx]

**Note S6.** **Comparison of MMSpa’s performance with recently developed methods (stCMGAE, SpaMask, MAEST, m2ST, and SpaDo)**

*a) Difference discussion between MMSpa and stCMGAE, SpaMask, MSEST, m2ST*

For MMSpa, SpaMask, stCMGAE, MAEST, and m2ST, all of which utilize the “masking” technique in graph neural networks for clustering spatial transcriptomics (ST) data, we have provided a comprehensive comparison of their technical differences, advantages, and disadvantages from a methodological perspective:

(1) m2ST: m2ST adopts a Graph Attention Network (GAT)-based framework without special data preprocessing. Initially, a shared encoder generates the preliminary embeddings, which are then re-encoded by multiple independent encoders to produce diverse representations, with corresponding decoders reconstructing the masked gene expressions. The advantage of m2ST is its ability to generate diverse representations. However, m2ST’s performance heavily relies on the configuration and number of the multiple encoders/decoders, requiring adjustments for different ST data types.

(2) MAEST: Similar to m2ST, MAEST does not involve any special preprocessing and also uses a GAT-based framework with multiple encoders. However, MAEST introduces two key innovations: ① an NLP layer for further processing the preliminary embedding to recover spatial gene expression, and ② an additional encoder for contrastive learning. While contrastive learning can improve generalization, it remains highly sensitive to data augmentation strategies, complicating the MAEST’s adaptation to different ST platforms.

(3) stCMGAE: stCMGAE applies PCA for data preprocessing and combines a GCN+NLP framework with masking for contrastive learning. Its main advantage is the integration of GCN and NLP for contrastive learning, enhancing feature extraction. However, since its spatial graph structure completely relies on KNN from spatial coordinates, and the GCN encoder suffers from over-smoothing, making it difficult to distinguish fine-grained domain boundaries, especially in heterogeneous tissues.

(4) SpaMask: SpaMask uses PCA for data preprocessing, adopts a GCN+NLP framework, and applies masking to both nodes and edges. While the dual masking strategy is comprehensive, the spatial graph is constructed purely based on spatial physical distance, without excluding false positives, which may lead to incorrect graph construction and adversely affect model performance.

(5) MMSpa:

While MMSpa also uses a masking strategy, it stands out in three key points from the methodological perspective.

① Biologically driven edge removal strategy for spatial graph enhancement

MMSpa uses a joint modeling of biological functional similarity and physical proximity to remove noisy edges from the original KNN graph, enhancing the spatial graph quality. This fundamentally reduces noisy edges in the spatial graph, significantly enhancing the clarity of domain boundary delineation in highly heterogeneous or highly complex tissues. In contrast, the other methods (stCMGAE, SpaMask, MAEST, and m2ST) construct spatial graphs based only on physical proximity, retaining noisy edges that could distort the domain boundary delineation, especially in complex tissues.

② Dynamic self-attention mechanism for fine-grained sub-domains identification

MMSpa’s use of GAT enables dynamic self-attention, overcoming the over-smoothing issue inherent in GCN-based encoders. This attention mechanism allows MMSpa to distinguish between fine-grained sub-domains, making it particularly effective in identifying subtle variations within highly heterogeneous tissues. In comparison, GCN-based models like stCMGAE and SpaMask may suffer from over-smoothing when aggregating node weights. As the number of layers increases, the learned node feature embeddings become more and more similar, resulting in inaccurate performance of fine-grained identification.

③ Streamlined but robust autoencoder frame

MMSpa avoids the complexity of multi-encoder/decoder structures and excessive reliance on contrastive learning, instead adopting a streamlined graph autoencoder. This reduces performance fluctuations and enhances robustness across different datasets and platforms. Unlike m2ST and MAEST, which depend on multiple encoders/decoders and NLP modules, MMSpa achieves superior interpretability and stability, making it more adaptable to diverse spatial transcriptomics data.

While MMSpa provides a significant advantage in handling noisy edges and enhancing domain boundary identification, its performance is still somewhat dependent on the quality of the spatial transcriptomics data. In datasets with significant noise or low-quality spatial information, the biologically-driven edge removal strategy may not be effective, potentially leading to less accurate sub-domain identification. However, we believe in high-quality ST datasets application, MMSpa’s advantages remain prominent.

*b) Difference discussion between MMSpa and SpaDo*

MMSpa and SpaDo are two fundamentally different domain identification algorithms. MMSpa is based on an unsupervised deep learning model framework, while SpaDo is a semi-supervised algorithm, relying on existing deconvolution algorithms or single-cell annotation algorithms, and then performs hierarchical clustering.

It is important to highlight the significant dependency of SpaDo on external algorithms, which brings several limitations. Firstly, in the context of spot ST dataset applications, it is necessary to obtain the deconvolution results before SpaDo, making its domain identification performance highly dependent on the deconvolution accuracy of the spot ST datasets. Additionally, this dependency brings various challenges for users, such as the need to first acquire single-cell reference data and then install an additional deconvolution algorithm package. Only after obtaining the deconvolution results can users proceed with SpaDo.

Secondly, for single-cell ST data applications, SpaDo relies on cell-type annotations. However, it is difficult to accurately label each single-cell dataset with cell type information, and tools for cell type annotation may not always be reliable.

Finally, the embedding used in SpaDo’s clustering is essentially derived from either the deconvolution results or cell-type annotations. In some extreme cases, such as when only a few cell types are present, the dimensionality of the embedding may be too low, directly affecting domain identification performance. Furthermore, SpaDo depends on Jensen-Shannon Divergence (JSD) based hierarchical clustering, leading to high memory consumption when working with data of high dimensionality.

In contrast, MMSpa not only enables user-friendly unsupervised domain identification but also achieves superior domain identification performance compared to SpaDo. We have comprehensively demonstrated this superior capability in the above (1) to (4) sections.
