# Supplementary material for: MMSpa is a deep learning-based tool that enhances the identification of spatial domains in spatial transcriptomics studies
Source: PLoS Biol. 2026 Jan 5;24(1):e3003580. doi: 10.1371/journal.pbio.3003580 (PMC12768284; doi:10.1371/journal.pbio.3003580)
Supplement: S7 Note — (DOCX) [file pbio.3003580.s028.docx]

**Note S7.** **Comparison of the existing spatial domain identification methods**

We compared SpaMask with nine existing spatial domain identification methods: SpaceFlow[1], conST[2], GraphST[3], SEDR[4], STAGATE[5], stCMGAE[6], SpaMask[7], MAEST[8], and SpaDo[9]. Each method was implemented according to the tutorial on their websites or GitHub repositories. We set the same number of clusters across methods or adjusted the resolution to ensure each method identified the same number of spatial domains. We used the default parameters for other parameters.

**(1) SpaceFlow:** We used the Python package SpaceFlow version 1.0.4, based on the tutorial at <https://github.com/hongleir/SpaceFlow/blob/master/tutorials/seqfish_mouse_embryogenesis.ipynb>.

The preprocessing steps involved filtering genes expressed in fewer than three cells and cells containing fewer than 100 gene counts. Gene expression was log-normalized, and the top 3000 HVGs were selected (If fewer than 3000 genes were available, all genes would be used for further steps). We used the default parameters as recommended in the tutorial. The clustering method was set to Leiden, with the resolution parameter manually specified. To ensure fairness, we used conST’s res_search_fixed_clus() function to automatically select the resolution for SpaceFlow.

**(2) conST:** We utilized the Python package conST, according to the tutorial at <https://github.com/ys-zong/conST/blob/main/conST_cluster.ipynb>.

For each dataset, we applied the recommended parameters from the tutorial. The clustering approach was set to the default choice of Leiden, and its resolution parameter was automatically selected via the res_search_fixed_clus() function provided by the conST package.

**(3) GraphST:** We implemented the Python package GraphST version 1.1.1, following the tutorial found at <https://deepst-tutorials.readthedocs.io/en/latest/Tutorial%201_10X%20Visium.html>.

For the 10X Visium datasets, we set the datatype parameter to “10X”, and for Stereo-Seq datasets, it was set to “Stereo”. For the STARmap, osmFISH, and MERFISH datasets, we first loaded the data using the read_h5ad() function from the SCANPY Python package. Subsequently, we used the GraphST() function to define the model and the train() function to train it. The clustering approach was set to the default choice of mclust. We used all other recommended parameters in the tutorial.

**(4) SEDR:** The Python package SEDR version 1.1.1 was used, following the tutorial from <https://sedr.readthedocs.io/en/latest/Tutorial1_Clustering.html>.

We applied the recommended parameters to filter and log-normalize the top 2000 highly variable HVGs. For datasets with fewer than 2,000 genes, all genes would be used for further steps. For osmFISH and MERFISH datasets, which contain only 33 and 155 genes, respectively, we modified the num_pcs parameter in the PCA() function from the default 200 to 15 for osmFISH and 50 for MERFISH. The clustering approach was set to the default mclust, with the number of clusters same as the number of ground truth clusters. All other parameters followed the tutorial recommendations.

**(5) STAGATE:** We utilized the Python package STAGATE_pyG version 1.0.0, guided by the <https://stagate.readthedocs.io/en/latest/index.html>.

We applied the recommended parameters to obtain 3000 log-normalized HVGs. For datasets with fewer than 3000 genes, all available genes would be used for further steps. For the 10X Visium dataset, the number of neighbors (the parameter k in the STAGATE_pyG.Cal_Spatial_Net() function) for constructing the spatial graph was set to 6 (default), while for other datasets, it was set to 8, as recommended in the tutorial to include 6–15 neighbors. The clustering approach was set to the default mclust, with the number of clusters same as the number of ground truth clusters. All other parameters followed the tutorial recommendations.

**(6) stCMGAE:** We implemented stCMGAE according to the tutorial Tutorial.ipynb in <https://github.com/donghaifang/stCMGAE>. The clustering approach was set to the default choice of kmeans. We applied the default selection to obtain 2000 highly variable genes and the first 200 PCs as input features. For osmFISH and MERFISH datasets, which contain only 33 and 155 genes, we selected the first 15 PCs and 50 PCs for them, respectively. We used the “KNN” graph model, and set the parameter k_cutoff to 12. The clustering approach was set to the default choice of kmeans. All other parameters followed the tutorial recommendations.

**(7) SpaMask:** We implemented SpaMask according to tutorials in <https://github.com/wenwenmin/SpaMask> and the parameter selection suggestions in the original paper. We applied the default selection to obtain 2000 highly variable genes and the first 200 PCs as input features. For osmFISH and MERFISH datasets, which contain only 33 and 155 genes, we selected the first 15 PCs and 50 PCs for them, respectively. We used the “KNN” graph model, and set the parameter k_cutoff to 12. The default node mask rate and edge mask rate were set to 0.3 and 0.4, respectively. For high-resolution datasets (including the #E9.5_E1S1, #E9.5_E2S3, MERFISH, osmFISH, and STARmap datasets), we followed the suggestion in the original paper to increase the edge mask rate to 0.5. The default weight factor for controlling the reconstruction loss and contrastive loss was set to 0.7. Parameters for multi-slice analyses were set to be the same as the tutorial TutorialDonor.ipynb in <https://github.com/wenwenmin/SpaMask>. The clustering approach was set to the default choice of kmeans. All other parameters followed the tutorial recommendations.

**(8) SpaDo:** We utilized the R package SpaDo version 1.2.0, guided by the tutorial provided on <https://github.com/bm2-lab/SpaDo>, and followed the recommended parameters.

Specifically, the distance parameter in DistributionDistance() function was set to the default JSD. For the single-cell resolution ST datasets, we set the parameter “user_offered” to False by using “user_offered=F” in InitialClustering() function to obtain preliminary cell type labels. For spot resolution ST datasets, we used the following single-cell reference to obtained the deconvolution results:

For the 10X DLPFC ST dataset, the corresponding single-cell reference used for spot deconvolution was acquired from <https://libd-snrnaseq-pilot.s3.us-east-2.amazonaws.com/SCE_DLPFC-n3_tran-etal.rda>. For all the 10X Mouse Brain ST datasets, the single-cell reference used for spot deconvolution was acquired from mouse whole cortex and hippocampus tissues (https://portal.brain-map.org/atlases-and-data/ rnaseq/mouse-whole-cortex-and-hippocampus-10x). For the 10X Human Breast cancer ST dataset, the single-cell reference used for spot deconvolution was acquired from the database DISCO[10]. We downloaded the preprocessed dataset from GraphST[3] (<https://zenodo.org/record/6925603#.YuM5WXZBwuU>). For the human PDAC ST dataset, the single-cell reference used for spot deconvolution was acquired from GSE111672 (PDAC-A)[11].

**(9) MAEST:** We implemented MAEST according to example code DLPFC.py in <https://github.com/clearlove2333/MAEST>. We followed the data preprocessing steps of the original paper: The clustering approach was set to mclust. The top 3000 most highly variable genes were selected as input features. In the “KNN” spatial graph construction, for each spot, its neighbors are defined as the k=3 nearest points. All other parameters followed the tutorial recommendations.

**Reference**

1. Ren H, Walker BL, Cang Z, Nie Q. Identifying multicellular spatiotemporal organization of cells with SpaceFlow. Nat Communications. 2022;13(1):4076. doi: 10.1038/s41467-022-31739-w.

2. Zong Y, Yu T, Wang X, Wang Y, Hu Z, Li Y. conST: an interpretable multi-modal contrastive learning framework for spatial transcriptomics. bioRxiv. 2022:2022.01.14.476408. doi: 10.1101/2022.01.14.476408.

3. Long Y, Ang KS, Li M, Chong KLK, Sethi R, Zhong C, et al. Spatially informed clustering, integration, and deconvolution of spatial transcriptomics with GraphST. Nat Communications. 2023;14(1):1155. doi: 10.1038/s41467-023-36796-3.

4. Xu H, Fu H, Long Y, Ang KS, Sethi R, Chong K, et al. Unsupervised spatially embedded deep representation of spatial transcriptomics. Genome Medicine. 2024;16(1):12. doi: 10.1186/s13073-024-01283-x.

5. Dong K, Zhang S. Deciphering spatial domains from spatially resolved transcriptomics with an adaptive graph attention auto-encoder. Nat Communications. 2022;13(1):1739. doi: 10.1038/s41467-022-29439-6.

6. Fang D GY, Wang Z, Zhu F, Min W. Contrastive Masked Graph Autoencoders for Spatial Transcriptomics Data Analysis. In: Peng W CZ, Skums P., editor. ISBRA; Singapore: Springer; 2024. p. 76-88.

7. Min W, Fang D, Chen J, Zhang S. SpaMask: Dual masking graph autoencoder with contrastive learning for spatial transcriptomics. PLOS Computational Biology. 2025;21(4):e1012881. doi: 10.1371/journal.pcbi.1012881.

8. Zhu P, Shu H, Wang Y, Wang X, Zhao Y, Hu J, et al. MAEST: accurately spatial domain detection in spatial transcriptomics with graph masked autoencoder. Briefings in Bioinformatics. 2025;26(2):bbaf086. doi: 10.1093/bib/bbaf086.

9. Duan B, Chen S, Cheng X, Liu Q. Multi-slice spatial transcriptome domain analysis with SpaDo. Genome Biology. 2024;25(1):73. doi: 10.1186/s13059-024-03213-x.

10. Li M, Zhang X, Ang KS, Ling J, Sethi R, Lee Nicole Yee S, et al. DISCO: a database of Deeply Integrated human Single-Cell Omics data. Nucleic acids research. 2021;50(D1):D596-D602. doi: 10.1093/nar/gkab1020 %J Nucleic Acids Research.

11. Moncada R, Barkley D, Wagner F, Chiodin M, Devlin JC, Baron M, et al. Integrating microarray-based spatial transcriptomics and single-cell RNA-seq reveals tissue architecture in pancreatic ductal adenocarcinomas. Nature Biotechnology. 2020;38(3):333-42. doi: 10.1038/s41587-019-0392-8.
